# A revision of the Oriental species of *Bolitogyrus* Chevrolat (Coleoptera, Staphylinidae, Staphylininae)

**DOI:** 10.3897/zookeys.664.11881

**Published:** 2017-03-29

**Authors:** Adam J. Brunke

**Affiliations:** 1 Canadian National Collection of Insects, Arachnids and Nematodes, Agriculture and Agri-Food Canada, Neatby Building, 960 Carling Avenue, Ottawa, Ontario, Canada, K1A 0C6 (Current address); 2 3rd Department of Zoology, Natural History Museum of Vienna, Burgring 7, Vienna, Austria, 1010-AT

**Keywords:** Staphylinini, Cyrtoquediina, boreotropics, Asia, taxonomy, primary forest

## Abstract

The Oriental species of the relictual genus *Bolitogyrus* are revised based on 200 specimens. An updated description of the genus is provided, including additional putative synapomorphies. Fifty valid Oriental species are diagnosed herein and the following nineteen are described as new to science: *B. concavus* **sp. n.**; *B. confusus* **sp. n.**; *B. himalayicus* **sp. n.**; *B. khasiensis* **sp. n.**; *B. luteus* **sp. n.**; *B. mulayitensis* **sp. n.**; *B. nanus* **sp. n.**; *B. nokrek* **sp. n.**; *B. pecki* **sp. n.**; *B. pederseni* **sp. n.**; *B. phukhieo* **sp. n.**; *B. rougemonti* **sp. n.**; *B. sepilok* **sp. n.**; *B. schillhammeri* **sp. n.**; *B. smetanai* **sp. n.**; *B. solodovnikovi* **sp. n.**; *B. temburong* **sp. n.**; *B. tigris* **sp. n.**; and *B. tumidus* **sp. n.** The following synonymies are proposed: *Cyrtothorax borneensis* Cameron, 1942, **syn. n.** = *Cyrtothorax caesareus* Bernhauer, 1915; *Cyrtothorax octomaculatus* Cameron, 1937 **syn. n.** = *Quedius ornatipennis* Wendeler, 1927. Quedius (Raphirus) ornatipennis is moved to *Bolitogyrus* as *B. ornatipennis* (Wendeler), **comb. n.** A lectotype is designated for *Cyrtothorax rufipennis* Cameron, 1937. Several species are named in recognition of conservation efforts to protect tropical primary forests in Asia that are important to the survival of many *Bolitogyrus* species. All available bionomic and distributional data for Oriental *Bolitogyrus* are summarized, and an identification key is provided.

## Introduction

Species of the genus *Bolitogyrus* are rarely collected specialists of fungusy deadwood in the humid forests of the Neotropical and Oriental regions (Brunke and Solodovnikov 2014). Specimens of most species are predictably (and often solely) collected from primary or near-primary, usually protected, forests with large fallen or standing trees (Brunke and Solodovnikov 2014, Brunke pers. obs.). Thus, many species of *Bolitogyrus* may be at high risk of extinction without adequate designation of forest preserves. A widely disjunct distribution in the New and Old World tropics, similar to several other Staphylinini, suggests that the lineage is an ancient Eocene relict, previously widespread across the northern hemisphere when global climate was far warmer and more equable (Brunke and Solodovnikov 2013). *Bolitogyrus* is now classified as a member of the relictual Staphylinini subtribe Cyrtoquediina (Brunke et al. 2016), a placement supported by both morphological and molecular data (Chani-Pose et al. 2017). Other members of the subtribe include the European *Astrapaeus ulmi* (Rossi), Oriental *Quwatanabius* Smetana and Neotropical *Cyrtoquedius* Bernhauer, with one species in the southern Nearctic (Brunke et al. 2016). These taxa can be recognized as cyrtoquediines based on a row of coarse, epipleural punctures (Brunke et al. 2016). The monophyly of *Bolitogyrus* is rigorously supported and morphological characters support reciprocal monophyly of single Oriental and Neotropical lineages (Brunke and Solodovnikov 2014, Brunke et al. 2016).

Recently, all Neotropical species of *Bolitogyrus* were revised and a key was provided for their identification (Brunke and Solodovnikov 2014). Unlike the Neotropical species, which had received very little modern taxonomic attention, Oriental species of *Bolitogyrus* have gradually been discovered until the most recent description of seven Chinese species by Cai et al. (2015). As species concepts in Oriental *Bolitogyrus* have become far more complex than their original descriptions and taxonomic treatments of the genus are becoming more regionally focused (e.g., China), a broad revision of the Oriental species is greatly needed. Therefore, the present work aims to refine the morphological definition of *Bolitogyrus*, critically review all described Oriental species, describe previously unrecognized diversity and summarize all available data on these taxa.

## Material and methods

### Specimens

This study is based on 200 specimens that are deposited in the following collections:

**BMNH** Natural History Museum, London, U.K. (R. Booth)

**cAss** Personal collection of V. Assing, Hannover, Germany

**cHay** Personal collection of Y. Hayashi, Kawanishi City, Japan

**cRou** Personal collection of G. de Rougemont, London, U.K.

**cSch** Personal collection of M. Schülke, Berlin, Germany

**cShi** Personal collection of Y. Shibata, Tokyo, Japan

**CNC** Canadian National Collection of Insects, Arachnids and Nematodes, Ontario, Canada (A. Smetana, A. Davies)

**SDEI** Senckenberg Deutsches Entomologisches Institut, Müncheberg, Germany (S. Blank)

**SEMC** Snow Entomological Collection, Biodiversity Institute, Kansas, U.S.A. (Z. Falin)

**FMNH** Field Museum of Natural History, Illinois, U.S.A. (J. Boone, M. Thayer, A. Newton)

**IRSNB** Institut royal des Sciences Naturelles de Belgique, Brussels, Belgium (Y. Gérard, T. Struyve)

**IZCAS** Chinese Academy of Sciences, Institute of Zoology, Beijing, China (X. Li, H. Zhou)

**MHNG** Muséum d’Histoire Naturelle, Geneva, Switzerland (G. Cuccodoro)

**MMUE** Manchester Museum, Manchester, U.K. (D. Logunov)

**NHMO** Natural History Museum, University of Oslo, Oslo, Norway (V. Gusarov)

**NHRS** Naturhistoriska Riksmuseet, Stockholm, Sweden (J. Bergsten)

**NMHB** Naturhistorisches Museum, Basel, Switzerland (M. Geiser)

**NMW** Naturhistorisches Museum Wien, Vienna, Austria (H. Schillhammer)

**OUMNH** Oxford Museum of Natural History, Oxford, U.K. (J. Hogan)

**RMNH** Naturalis Biodiversity Centre, Leiden, The Netherlands (Hans Huijbregts)

**SNUC** Shanghai Normal University, Shanghai, China (L. Tang, Z.-W. Yin)

**USNM** Smithsonian Institute, National Museum of Natural History, Washington D.C., U.S.A. (D. Furth, F. Shockley)

**ZIN** Zoological Institute, Russian Academy of Sciences, St. Petersburg, Russia (B. Korotyaev)

**ZMHB** Museum für Naturkunde der Humboldt-Universität, Berlin, Germany (M. Uhlig)

**ZMUC** Zoological Museum, Natural History Museum of Denmark, University of Copenhagen, Denmark (S. Selvantharan, A. Solodovnikov)

### Specimen dataset

Specimens with GIS coordinates on labels were georeferenced using Google Earth. Specimens with only country or ‘country, province/state’ information were not georeferenced. Specimen data were exported to Darwin Core Archive (DwCA) format, are available under a Creative Commons CCZero 1.0 License and were registered with GBIF.

### Microscopy, illustration, photography and mapping

All specimens were examined using a Nikon 745T stereomicroscope. To allow for the routine dissection of the terminal abdominal segments (including the aedeagus), distilled water was applied directly to the tip of the abdomen using a fine paintbrush. As a precaution against DNA degradation, specimens examined in the present study were never subjected to high ambient humidity relaxing chambers or entirely submersed in water. This was in direct contrast to the specimens dissected for Brunke and Solodovnikov (2014), which may be less suitable for future amplification of DNA. Genitalia were cleared in a 10% potassium hydroxide solution and then washed with distilled water, then with 70% alcohol and finally placed in glycerin for observation. Genitalia were placed in glycerin filled vials for long-term storage, which were pinned with their respective specimen.

Line illustrations were performed in Adobe Illustrator CS6 based on photographs. Photomontage was accomplished using a motorized Nikon SMZ25 microscope and NIS Elements BR v4.5. Photos were processed in Adobe Photoshop CS6. Distribution maps were created using QGIS 2.18 as in Brunke and Solodovnikov (2014).

### Measurements and character variability

All measurements were made using a live measurement module within NIS Elements BR v4.5. Measurements were taken as listed below, but only proportional (HW/HL, PW/PL, EW/EL, ESut/PL, PW/HW) and forebody measurements were stated directly in descriptions due to a wide variability in body size. Total body length is generally not diagnostic of *Bolitogyrus* species and was not measured due to the contractile nature of the abdomen.

**HL** Head Length, at middle, from the anterior margin of frons to the nuchal ridge.

**HW** Head Width, the greatest width, including the eyes.

**PL** Pronotum Length, at middle.

**PW** Pronotum Width, greatest width.

**EL** Elytral Length, greatest length taken from level of the anterior most large, lateral macroseta to apex of elytra (this seta can be seen in Fig. 9 B, C and F). Its length approximates the length of the elytra not covered by the pronotum and therefore contributing to the forebody length.

**EW** Elytral Width, greatest width.

**ESut** Sutural Length, length of elytral suture.

**Forebody** HL + PL + EL.

Greater intraspecific variability in female tergite X and a general lack of male-associated females made it difficult to incorporate female genitalia as fully in the Oriental species concepts compared to those of the Neotropics. Therefore, female genitalia were not illustrated though they were described where possible. Notches in the apex of female tergite X are typical of many Oriental *Bolitogyrus* but the size of this notch is subject to large intraspecific variation in some species. For example, the female paratypes of *B. lasti* Rougemont lack the notch but a small notch is present on additional female specimens examined herein. The median completion of the transverse basal line of male tergite VIII was used in previous treatments of *Bolitogyrus* (Brunke and Solodovnikov 2014, Cai et al. 2015) but significant intraspecific variability was observed in the Oriental species and so this structure is not included in descriptions.

## Data resources

A specimen level dataset was made available as a Darwin Core Archive and was deposited in GBIF at http://ipt.pensoft.net/resource?r=oriental_bolitogyrus

## Results

### Putative synapomorphies of *Bolitogyrus*

Brunke and Solodovnikov (2014) provided a redescription of *Bolitogyrus* and suggested that the following character states were putative synapomorphies for the genus: spineless lateral face of the hind tibia, antennomeres 1-5 without tomentose pubescence and elytra with scattered, asetose punctures. Three additional character states unique to *Bolitogyrus* (within Cyrtoquediina), were discovered in the course of this revision: abdominal sternite IV with basal sternal carina acutely projected at middle, single epipleural row of setae situated very close to or in contact with epipleural ridge, and epipleural ridge thickened.

### Species

**Figure 1.**
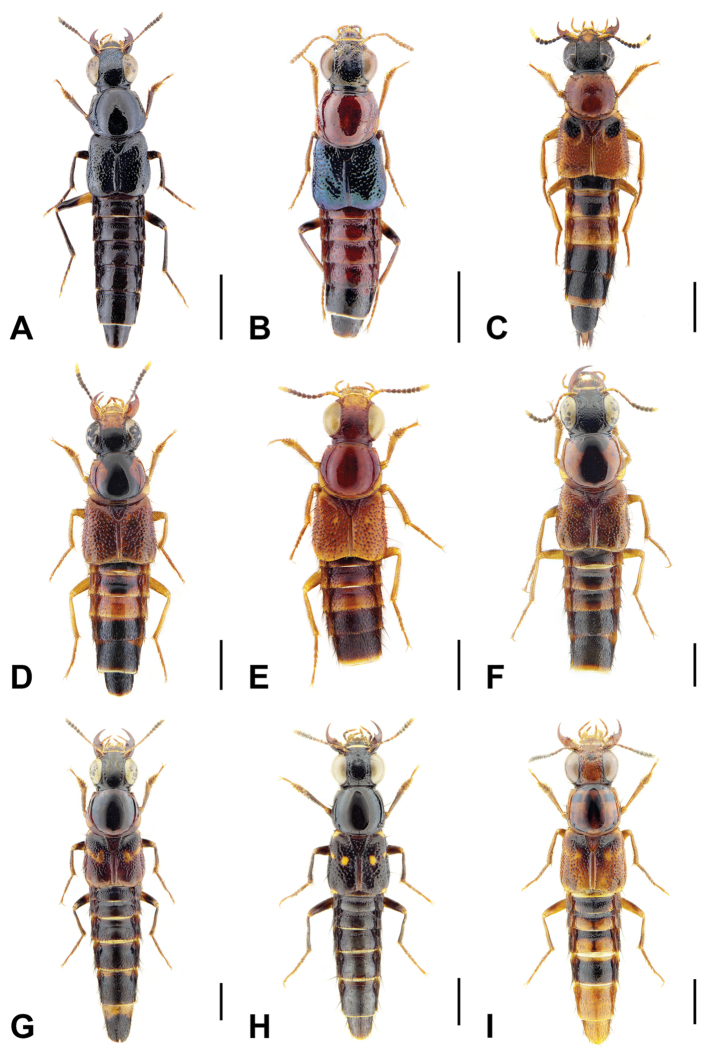
Dorsal habitus of *Bolitogyrus electus* Smetana & Zheng (**A**), *B. kitawakii* (Smetana & Zheng) (**B**), *B. caesareus* (Bernhauer) (**C**), *B. proximus* (Cameron) (**D**), *B. rufipennis* (Cameron) (**E**), *B. pederseni* Brunke (**F**), *B. nokrek* Brunke (**G**), *B. lasti* Rougemont (**H**) and *B. tigris* Brunke (**I**). Scale bars: 2 mm.

**Figure 2.**
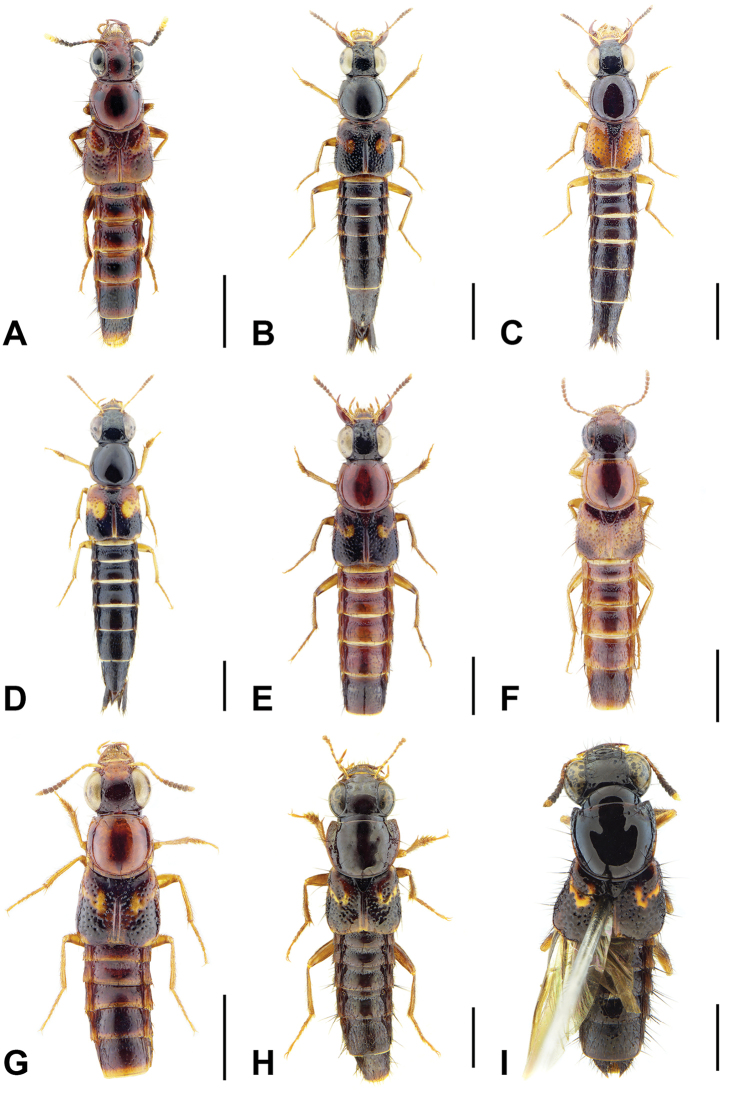
Dorsal habitus of *Bolitogyrus sepilok* Brunke (**A**), *B. schillhammeri* Brunke (**B**), *B. flavus* Yuan et al. (**C**), *B. fukiensis* (Scheerpeltz) (**D**), *B. solodovnikovi* Brunke (**E**), *B. smetanai* Brunke (**F**), *B. elegans* (Cameron) (**G**), male *B. signatus* (Cameron) (**H**) and female *B. signatus* (**I**). Scale bars: 2 mm.

A revision of the Oriental *Bolitogyrus* resulted in the discovery of 19 new species, 2 new synonyms and the transfer of *Quedius ornatipennis* Wendeler to *Bolitogyrus*, resulting in a total of 50 valid species (Figs 1–2). The number of *Bolitogyrus* species worldwide now stands at 78 but can be expected to be well over one hundred with additional collecting. Male characters continue to be unknown for *Bolitogyrus fukiensis* (Scheerpeltz), the only Oriental species still known only from female specimens. The species were organized into eight putative species groups to facilitate comparisons between species, but it is recognized that they may not reflect monophyletic groups. Four species were not placed to any of the species groups and in general, great difficulty was experienced to form useful groupings based on multiple characters. Currently the richest country is China with 15 species but every country in the Oriental range of *Bolitogyrus* is still heavily under sampled. For example, the genus is entirely unknown from Sumatra and a single but widespread species is known from mainland Malaysia. At least fifteen new species represented by only females were seen in the course of this study and many others are expected, especially with more widespread use of micro-fogging of fungusy logs (see Brunke and Solodovnikov 2014 for details).

### Checklist of Oriental *Bolitogyrus*

Electus Group

*Bolitogyrus electus* Smetana & Zheng, 2000

*Bolitogyrus uncus* Cai et al., 2015

*Bolitogyrus confusus* Brunke, sp. n.

*Bolitogyrus huanghaoi* Hu et al., 2011

*Bolitogyrus metallicus* Cai et al., 2015

*Bolitogyrus nigerrimus* Yuan et al., 2007

*Bolitogyrus nigropolitus* Smetana, 2000

*Bolitogyrus cyanipennis* (Zheng, 1988)

*Bolitogyrus kitawakii* Smetana & Zheng, 2000

Caesareus Group

*Bolitogyrus caesareus* (Bernhauer, 1915)

*Bolitogyrus proximus* (Cameron, 1942)

*Bolitogyrus temburong* Brunke, sp. n.

*Bolitogyrus rufipennis* (Cameron, 1937)

Carnifex Group

*Bolitogyrus carnifex* (Fauvel, 1878)

*Bolitogyrus pederseni* Brunke, sp. n.

*Bolitogyrus vietnamensis* (Scheerpeltz, 1974)

*Bolitogyrus elegantulus* Yuan et al., 2007

*Bolitogyrus phukhieo* Brunke, sp. n.

*Bolitogyrus magnimaculosus* Cai et al., 2015

*Bolitogyrus nokrek* Brunke, sp. n.

Lasti Group

*Bolitogyrus lasti* Rougemont, 2001

*Bolitogyrus tigris* Brunke, sp. n.

Luteus Group

*Bolitogyrus luteus* Brunke, sp. n.

*Bolitogyrus sepilok* Brunke, sp. n.

Pictus Group

*Bolitogyrus pictus* Smetana & Zheng, 2000

*Bolitogyrus rougemonti* Brunke, sp. n.

*Bolitogyrus schillhammeri* Brunke, sp. n.

*Bolitogyrus profundus* Cai et al., 2015

*Bolitogyrus concavus* Brunke, sp. n.

Vulneratus Group

*Bolitogyrus vulneratus* (Fauvel, 1878)

*Bolitogyrus flavus* Yuan et al., 2007

*Bolitogyrus rufomaculatus* (Shibata, 1979)

*Bolitogyrus depressus* Cai et al., 2015

*Bolitogyrus tumidus* Brunke, sp. n.

*Bolitogyrus taiwanensis* (Hayashi, 1991)

*Bolitogyrus fukiensis* (Scheerpeltz, 1974)

Loculus Group

*Bolitogyrus loculus* Cai et al., 2015

*Bolitogyrus hainanensis* Cai et al., 2015

*Bolitogyrus solodovnikovi* Brunke, sp. n.

*Bolitogyrus feai* Brunke, sp. n.

*Bolitogyrus mulayitensis* Brunke, sp. n.

*Bolitogyrus smetanai* Brunke, sp. n.

*Bolitogyrus khasiensis* Brunke, sp. n.

*Bolitogyrus himalayicus* Brunke, sp. n.

*Bolitogyrus nanus* Brunke, sp. n.

*Bolitogyrus pecki* Brunke, sp. n.

Species *incertae sedis*

*Bolitogyrus elegans* (Cameron, 1937)

*Bolitogyrus ornatipennis* (Wendeler, 1927), comb. n.

*Bolitogyrus doesburgi* (Scheerpeltz, 1974)

*Bolitogyrus signatus* (Cameron, 1932)

### Key to the Oriental species of *Bolitogyrus*

**Table:** 

1	Elytra without red, yellow or orange coloration (e.g., Fig. 1A–B); pronotum with two fine punctures in the dorsal row, anterior angles densely and deeply punctate, and sides of disc explanate laterally (Fig. 3B); prosternum with low, rounded, longitudinal ridge (Fig. 3D); China and northern portions of Myanmar, Laos and Thailand	**Electus Group**...**2**
–	Elytra with yellow, red or orange coloration; pronotum with one puncture (marginal) in the dorsal row, anterior angles more sparsely punctate (Fig. 3A), if sides of disc explanate, then pronotum strongly transverse (male of one Sri Lankan species, Fig. 2H); pronotal margin often expanded (as in Fig. 1E, 3A); prosternum without ridge (Fig. 3C); southern China, India, Nepal, Myanmar and Southeast Asia	**10**
2	Abdomen bicolored red and black, elytra bright metallic green to blue (Fig. 1B)	**3**
–	Abdomen entirely dark, elytra dark, with only faint metallic reflection (Fig. 1A)	**4**
3	Paramere with constricted stem, exposing median lobe in parameral view (Fig. 9E); apex of median lobe obtuse in parameral view (Fig. 9H); northeastern Sichuan , northern Chongqing and southern Shaanxi, China	***B. kitawakii* Smetana & Zheng**
–	Paramere vaguely constricted, not exposing median lobe in parameral view (Fig. 9A); apex of median lobe acute in parameral view (Fig. 9D); north-central Sichuan, China	***B. cyanipennis* (Zheng)**
4	Head with deeply impressed punctures, many punctures confluent, forming rows (Fig. 3F); Hubei and Guizhou, China	**5**
–	Head with regular, non-impressed punctures, most punctures clearly separated (Fig. 3E); Sichuan and Yunnan, China, northern Laos	**6**
5	Paramere with peg setae medially, on projected ridge (Fig. 9O); peg setae with median group extended clearly basad of marginal group (Fig. 9N); median lobe in lateral view without subapical teeth (Fig. 9L); Hubei, China	***B. metallicus* Cai et al.**
–	Paramere without projected ridge; peg setae with median group extended to no more than just behind level of marginal group (Fig. 9K); median lobe in lateral view with small subapical teeth (Fig. 9I); Guizhou, China	***B. nigerrimus* Yuan et al.**
6	Hind tibia entirely dark, as dark as darkened portion of femur (Fig. 3G); antennomere 6 as dark as more distal segments; paramere slightly to distinctly longer than median lobe; Yunnan province, south to northern Laos and Thailand	**7**
–	Hind tibia in lateral view with at least distal half distinctly lighter than darkened portion of femur (Fig. 3H); antennomere 6 paler than more distal segments; paramere shorter than median lobe; central Sichuan, China	***B. nigropolitus* Smetana**
7	Antennomeres 7–10 relatively elongate: 6 quadrate and 7 weakly transverse (Fig. 4A); paramere with attenuate apex (Fig. 8A, D, E)	**8**
–	Antennomeres 7–10 relatively transverse: 6 weakly, and 7 distinctly transverse (Fig. 4B); paramere with evenly converging sides (Fig. 8J,K)	**9**
8	Apex of median lobe in lateral view forming a more elongate triangle (Fig. 8B); paramere in lateral view with broad lateral projection (Fig. 8F); Central Yunnan, China	***B. electus* Smetana & Zheng**
–	Apex of median lobe in lateral view forming a shorter triangle (Fig. 8C); paramere in lateral view with sharp lateral projection (Fig. 8G); Western Yunnan, China	***B. uncus* Cai et al.**
9	Peg setae absent from broad oval shaped area along middle of paramere (Fig. 8O); median lobe in lateral view without expansion (Fig. 8N); Western Yunnan, China	***B. huanghaoi* Hu et al.**
–	Peg setae absent from only narrow strip along middle of paramere (Fig. 8K); median lobe in lateral view with distinct expansion (as in Fig. 8B); southeast Yunnan, China, and northern Laos (possibly northern Thailand)	***Bolitogyrus confusus* Brunke, sp. n.**
10	Head dark with frons distinctly paler, light orange (Fig. 1C, E)	**11**
–	Frons similar in color to rest of head (as in Fig. 1F)	**16**
11	Pronotal margin greatly expanded, at its widest point, more than four lateral puncture widths wide (Fig. 4C); large species, forebody length 5.2–6.5 mm	**Caesareus group**...**12**
–	Pronotal margin slightly to moderately expanded, at its widest point, no more than three lateral puncture widths wide (Fig. 4D); smaller species, forebody length 4.0–4.7 mm	**15**
12	Elytra with distinct black spot near humerus (Fig. 1C); aedeagus as in Fig. 10A–C; Borneo	***B. caesareus* (Bernhauer)**
–	Elytra without dark markings (Fig. 1D)	**13**
13	First two visible abdominal tergites without crisp, dark markings on disc; pronotum less transverse; Java (Fig. 1E)	***B. rufipennis* (Cameron)**
–	First two visible abdominal tergites with crisp, dark markings on disc; pronotum more transverse; Borneo (Fig. 1D)	**14**
14	Median lobe in parameral view evenly narrowed to apex (Fig. 10D–F); lowland (0–200 m)	***B. proximus* (Cameron)**
–	Median lobe in parameral view dilated near apex and then sharply narrowed (Fig. 10G–I); low montane (400–770m)	***B. temburong* Brunke, sp. n.**
15	Apical antennomere dark, not contrasting with previous segments (Fig. 2F); elytra more densely punctate, many punctures touching (Fig. 4F); western Thailand and southern Myanmar; aedeagus as in Fig. 17D–F	***B. smetanai* Brunke, sp. n.**
–	Apical antennomere pale, contrasting with previous segments (Fig. 2G); elytra more sparsely punctate, with wide shining spaces, about one puncture diameter wide (Fig. 4E); West Java; aedeagus as in Fig. 18A–C	***B. elegans* (Cameron)**
16	Dorsal surface of head mostly orange with some dark markings (Fig. 1I)	**17**
–	Dorsal surface of head entirely dark (Fig. 1H)	**19**
17	Elytra with yellow raised marking v-shaped (Fig. 4G); pronotal margin only minutely expanded (Fig. 4G); larger species (6.0–6.3 mm forebody length); southwestern India; aedeagus as in Fig. 12K–M	***B. tigris* Brunke, sp. n.**
–	Elytra with yellow raised marking transverse (Fig. 4H); pronotal margin moderately expanded (Figs 2A, 4H); smaller species (4.3–4.7 mm forebody length); western Thailand and Borneo	**Luteus Group**...**18**
18	Abdominal tergites IV–V each with strongly impressed and elongate punctures in basal impression (Fig. 5A); apical three antennomeres pale, contrasting with previous segments (Fig. 2A); Borneo; aedeagus as in Fig. 13E–G	***B. sepilok* Brunke, sp. n.**
–	Abdominal tergites IV–V each with typical circular punctures; only apical antennomere distinctly paler than previous segments (Fig. 4I); western Thailand; aedeagus as in Fig. 13A–D	***B. luteus* Brunke, sp. n.**
19	Pronotal margin greatly expanded, at its widest point, more than four lateral puncture widths wide (Figs 1G, 4C)	**Carnifex group**...**20**
–	Pronotal margin slightly to moderately expanded, at its widest point, no more than three lateral puncture widths wide (Figs 2B, 4D)	**26**
20	Base of elytra distinctly darker than rest of disc (Fig. 1G); Meghalaya, India; aedeagus as in Fig. 12D–G	***B. nokrek* Brunke, sp. n.**
–	Disc of elytra entirely reddish (except for pale markings) (as in Fig. 1F); southern China, Laos, Vietnam, Cambodia and Thailand	**21**
21	Peg setae arranged in one group removed from margin (Fig. 12A–C); Hainan, China	***B. magnimaculosus* Cai et al.**
–	Peg setae arranged in lateral and medial groups (Fig. 11C, F, I, L, O); mainland Oriental Region	**22**
22	Visible abdominal tergites 1–3 with relatively small dark median spots, occupying less than 1/3 of the tergal width (Fig. 5B); median lobe in parameral view (paramere removed) with basal pair of teeth placed medially (Fig. 11J, M); southern Yunnan, China, northern Laos and central Thailand	**23**
–	Visible abdominal tergites 1–3 with relatively wide dark median spots, occupying more than 1/3 of the tergal width (Fig. 5C); median lobe in parameral view (paramere removed) with basal pair of teeth placed laterally (Fig. 11A, D, G); southern Laos, Cambodia, southern Vietnam	**24**
23	Antennomeres 8–10 quadrate; paramere with apex only slightly narrowed (Fig. 11O); apex of median lobe in parameral view acuminate (Fig. 11M); central Thailand	***B. phukhieo* Brunke, sp. n**
–	Antennomeres 8–10 transverse; paramere strongly narrowed to apex (Fig. 11L); apex of median lobe in parameral view evenly converging to apex (Fig. 11J); southern Yunnan, China, northern Laos	***B. elegantulus* Yuan et al.**
24	Median lobe in lateral view with subapical tooth on distinct carina (Fig. 11E, H); northern Vietnam to southern Laos and northern Cambodia	**25**
–	Median lobe in lateral view with subapical tooth retracted from outline, not on carina (Fig. 11B); southern Vietnam and [likely southern] Cambodia	***B. carnifex* (Fauvel)**
25	Paramere with slender apical portion, peg setae arranged more in rows (Fig. 11I); median lobe in lateral view with apex more angulate (Fig. 11H); north of the Red River delta, northern Vietnam	***B. vietnamensis* (Scheerpeltz)**
–	Paramere with apical portion only weakly narrowed apicad, peg setae arranged loosely in two circular formations (Fig. 11F); median lobe in lateral view with apex knob-like (Fig. 11E); Bolaven Plateau in southern Laos and adjacent area of northern Cambodia	***B. pederseni* Brunke, sp. n.**
26	Elytra entirely reddish-orange (as in Fig. 1D); Java; aedeagus as in Fig. 18G–I	***B. doesburgi* (Scheerpeltz)**
–	Elytra at least partly dark	**27**
27	Pronotum widest at anterior angles; with distinct pronotal protuberance (Fig. 2H–I); Sri Lanka; aedeagus as in Fig. 18J–L	***B. signatus* (Cameron)**
–	Pronotum widest at middle or posterior third (Fig. 2G); without pronotal protuberance	**28**
28	Species distributed in northeastern India	**29**
–	Species distributed elsewhere	**32**
29	In lateral view, discal elytral markings not extending halfway to epipleural margin, therefore epipleuron at most pale at humerus and apex only (as in Fig. 5F); Khasi Hills, India; aedeagus as in Fig. 17G–H	***B. khasiensis* Brunke, sp. n.**
–	In lateral view, discal elytral markings extending halfway to epipleural margin, and often continuing laterad in both directions, epipleuron therefore broadly pale in at least basal half (as in Fig. 5G–H)	**30**
30	Minute species (3.5–3.7 mm forebody length); paramere without expansion in lateral view; Khasi Hills, Meghalaya and Himalaya of West Bengal, India	**31**
–	Average sized species (4.4 mm forebody length); Garo Hills, Meghalaya, India; paramere with subbasal expansion in lateral view (Fig. 15B); aedeagus as in Fig. 15A–C	***B. concavus* Brunke, sp. n.**
31	Apex of the median lobe in parameral view with single toothed carina (Fig. 17J); paramere with peg setae arranged in disorganized lateral row, apex with dense group (Fig. 17I); low elevation forest in valleys, Himalaya, West Bengal, India	**.*B. himalayicus* Brunke, sp. n.**
–	Apex of median lobe in parameral view with double-toothed carina (Fig. 17L); paramere with peg setae in sparse, single row (Fig. 17K); medium elevation forest in the Khasi Hills, Meghalaya	***B. nanus* Brunke, sp. n.**
32	In lateral view, discal elytral markings not extending halfway to epipleural margin, therefore epipleuron at most pale at humerus and apex only (Fig. 5F); West Java and southern India	**33**
–	In lateral view, discal elytral markings extending halfway to epipleural margin, and often continuing laterad in both directions, epipleuron narrowly to broadly pale at middle (Fig. 5G–H); not known from Java or southern India	**34**
33	Pale elytral marking v-shaped or with inner marking slightly triangular (Fig. 5F); disc of head with extremely shallow and sparse punctation; pronotal margin with distinct expansion (Fig. 5F); paramere entire (Fig. 18F); West Java	***B. ornatipennis* (Wendeler)**
–	Pale elytral marking always consisting of oval-shaped inner marking and small circular outer marking; disc of head with moderately impressed and dense punctation; pronotal margin without distinct expansion (Fig. 1H); paramere bilobed (Fig. 12H); southern India	***B. lasti* Rougemont**
34	Base of elytron with broad pale area extending to humerus (Figs 1C, 6A–C)	**35**
–	Medial and lateral elytral spots always distinguishable as separate markings, though usually fused to some degree (Figs 2B, 6D)	**38**
35	Pale basal part of elytra composed of both yellow to orange raised marking and slightly darker non-raised area (Fig. 6A–B)	**36**
–	Pale basal part of elytra composed only of yellow to orange raised area (Figs 1C, 6C)	**37**
36	Antennomeres less transverse, 7 weakly transverse (Fig. 6E); apex of hind femur entirely darkened (Fig. as in 2B, E); female tergite X with flattened disc; endemic to Taiwan; aedeagus as in Fig. 15G–I	***B. taiwanensis* (Hayashi)**
–	Antennomeres more transverse, 7 distinctly transverse (Fig. 6F); apex of hind femur with only ventral half darkened (Fig. 2D); female tergite X with raised disc; Fujian province, China; male unknown	***B. fukiensis* (Scheerpeltz)**
37	Punctures absent or distinctly sparse nearly in posterior half of sutural area (as in Fig. 6A); apex of hind femur entirely darkened (as in Fig. 2B, E); paramere strongly constricted apically (Fig. 16H); female tergite VIII with subparallel-sided notch (Fig. 7A); female tergite X with raised disc; low montane habitats (560–860 m)	***B. loculus* Cai et al.**
–	Sutural area with moderately dense regularly spaced punctures (Fig. 6C); apex of hind femur with only ventral half darkened; paramere not constricted apically (Fig. 13O); female tergite VIII with triangular notch (Fig. 7B); female tergite X without raised disc; lowland to low montane habitats (up to 1000 m), most common and widespread species of Oriental *Bolitogyrus*	***B. flavus* Yuan et al.**
38	Outer discal spot of elytra thinly connected to inner spot, to form a chevron or ‘v-shaped’ marking (Figs 2E, 5H, 7C)	**39**
–	Elytra with differently formed markings (as in Figs 2B, 5G, 6D)	**41**
39	Epipleuron with distinct dark area in apical 2/3 (Fig. 5H); Bolaven plateau in southern Laos; aedeagus as in Fig. 16I–K	***B. solodovnikovi* Brunke, sp. n.**
–	Epipleuron broadly pale throughout its length (Fig. 7C); Myanmar	**40**
40	Antennomeres 8 and 9 distinctly transverse (Fig. 7D); frons with distinct microsculpture; higher elevation (1800–1900 m); Mt. Mulayit, Kayin State, Myanmar; aedeagus as in Fig. 17A–C	***B. mulayitensis* Brunke, sp. n.**
–	Antennomeres 8 and 9 weakly transverse (Fig. 7E); frons with at most vague microsculpture; lower elevation (900–1100 m); Karen Hills, Kayin State, Myanmar; aedeagus as in Fig. 17D–F	***B. feai* Brunke, sp. n.**
41	Apex of hind femur entirely darkened (Fig. 2B); paramere with subbasal expansion in lateral view (Fig. 15B, arrow)	**Pictus Group** (part)...**42**
–	Apex of hind femur with, at most, only ventral half darkened (Fig. 2D); paramere without subbasal expansion in lateral view	**45**
42	Median lobe in lateral view strongly narrowed to thin apex (Fig. 14H); known from lowland rainforest (ca. 100 m) in western Thailand	***B. rougemonti* Brunke, sp. n.**
–	Median lobe in lateral view not strongly narrowed, apex distinctly broader (Fig. 14B,E,K); known from low montane to montane forests (>500 m) north of western Thailand	**43**
43	Occurring in the Shan Hills of Myanmar; aedeagus as in Fig. 14D–F	***B. schillhammeri* Brunke, sp.n.**
–	Occurring east of Myanmar: northern Thailand, Laos and southern Yunnan, China	**44**
44	Median lobe in lateral view slender and not distinctly narrowed to apex (Fig. 14K); median lobe in parameral view with acute apex (Fig. 14J)	***B. profundus* Cai et al.**
–	Median lobe in lateral view broader and strongly narrowed to hooked apex (Fig. 14B); median lobe in parameral view with obtuse apex (Fig. 14A)	***B. pictus* Smetana & Zheng**
45	Antennomere 7 distinctly transverse (Fig. 6F); female tergite X with wide triangular emargination (Fig. 7B); northern and southern Vietnam and Hainan, China	**46**
–	Antennomere 7 quadrate to weakly transverse (as in Fig. 6E); female tergite VIII with narrower, elongate emargination (Fig. 7A); northern Laos, southeastern China and Taiwan	**48**
46	Paramere narrowly bilobed (Fig. 16A); median lobe in lateral view with recurved apex (Fig. 16C); montane forests of Hainan Island, China	***B. hainanensis* Cai et al.**
–	Paramere not bilobed (Figs 13K, 17O); median lobe in lateral view hooked at apex or with small tooth but not recurved (Figs 13J, 17N); occurring in low hills of northern and southern Vietnam	**47**
47	Paramere with distinct lateral and medial rows of peg setae, slender (Fig. 17O); median lobe with pair of laterally placed basal teeth (Fig. 17M), apex in lateral view with only small tooth (Fig. 17N); low hills of northern Vietnam	***B. pecki* Brunke, sp.n.**
–	Paramere without distinct rows of peg setae (Fig. 13K); median lobe in lateral view hooked at apex (Fig. 13J); low hills of southern Vietnam	***B. vulneratus* (Fauvel)**
48	Northern Laos; median lobe in lateral view expanded near short apical portion (Fig. 15K)	***B. tumidus* Brunke, sp. n.**
–	Southeastern China and Taiwan; median lobe in lateral view expanded much more basally, apical portion longer (Fig. 15E,F)	**49**
49	Paramere exceeding apex of median lobe (Fig. 15E); median lobe with basal teeth and with weak expansion in lateral view (Fig. 15E); Taiwan	***B. rufomaculatus* (Shibata)**
–	Paramere shorter than apex of median lobe (Fig. 15F); median lobe without basal teeth and with large expansion in lateral view (Fig. 15F); northern Guangdong, China	***B. depressus* Cai et al.**

### Electus Group

**Figure 3.**
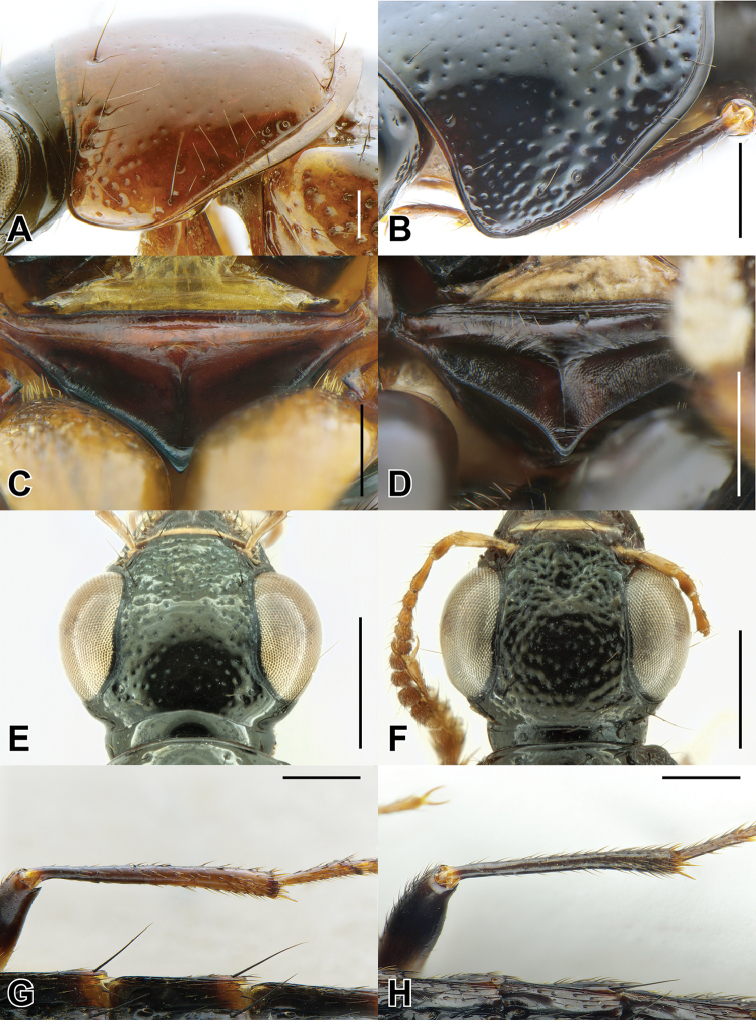
Pronotum, lateral: *Bolitogyrus caesareus* (Bernhauer) (**A**) and *B. electus* Smetana & Zheng (**B**). Prosternum: *B. proximus* (Cameron) (**C**) and *B. electus* (**D**). Dorsal head: *B. electus* (**E**) and *B. nigerrimus* Yuan et al. (**F**). Hind tibia: *B. nigerrimus* (**G**) and *B. electus* (**H**). Scale bars: 0.5 mm.

The Electus Group (*B. confusus*, *B. cyanipennis*, *B. electus*, *B. huanghaoi*, *B. kitawakii*, *B. metallicus*, *B. nigerrimus*, *B. nigropolitus* and *B. uncus*) consists of mostly dark colored species that all possess a rounded longitudinal ridge on the prosternum (Fig. 3D) and a pronotum with two punctures in the dorsal row (Fig. 1A–B), deeply punctate anterior angles, explanate lateral areas and a margin that is weakly expanded (Fig. 3B). A pair of lateral teeth, not found in any other *Bolitogyrus* species, are formed from a pair of very weakly to strongly produced carinae on the apical portion of the median lobe (Fig. 8B, H). Two reddish species have metallic green-blue elytra (Fig. 1B). As far as known, all species have an entire margin of female tergite VIII. The Electus Group was first proposed by Smetana (2000) based on the coarse punctation of the anterior angles of the pronotum. The distribution of this well-defined group is distinctly more northern than that of the others (Fig. 19A–B), and several species occur in the warm temperate montane forests of China, a transitional zone between subtropical and cold temperate forest, where they coexist with distinctly temperate beetle groups (*e.g.*, *Lathrobium* Gravenhorst (Assing 2013)).

#### Bolitogyrus electus

Taxon classificationAnimaliaColeopteraStaphylinidae

Smetana & Zheng, 2000

**Figure 4.**
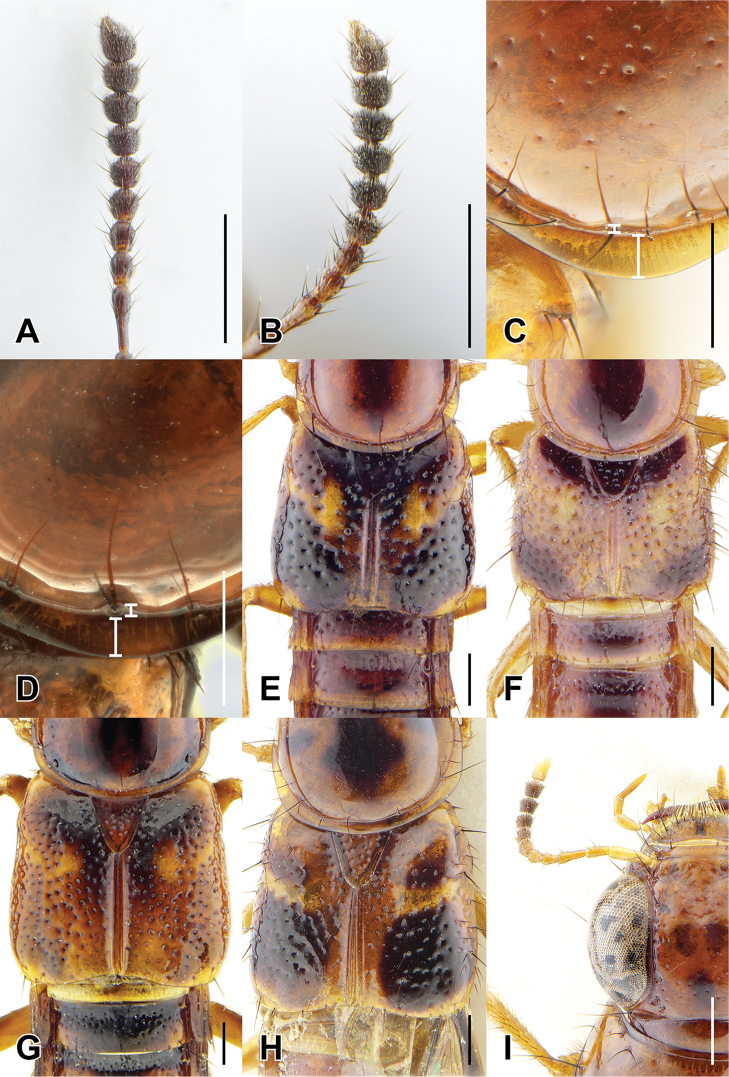
Antenna: *Bolitogyrus electus* Smetana & Zheng (**A**) and *B. confusus* Brunke (**B**). Posterior angle of pronotum: *B. caesareus* (Bernhauer) (**C**) and *B. elegans* (Cameron) (**D**). Elytra: *B. elegans* (**E**), *B. smetanai* Brunke (**F**), *B. tigris* Brunke (**G**) and *B. luteus* (**H**). Dorsal head: *B. luteus* (**I**). Scale bars: 0.5 mm (**A–C, E–I**), 1 mm (**D**).

Figs 1A , 3B, E, H , 4A , 8A, B, D, F, H , 19A (map)

Bolitogyrus elegans Smetana & Zheng, 2000a: 59.
Bolitogyrus electus Smetana & Zheng, 2000b: 465 (replacement name).
Bolitogyrus electus : Hu et al. 2011, misidentification of B. confusus.

##### Type locality.

Yulongshan, Yunnan, China.

##### Type material.

*Bolitogyrus electus* Smetana & Zheng.

**Holotype** (♂, NHMB). China, N-YUNNAN, Yulongshan mts., 2500–2800 m, GANHAIZI/LIJIANG road, lgt. D. Kral, 24-26/7/’90 [printed] / HOLOTYPE Bolitogyrus elegans A. Smetana, 1999 [red label] / AJB0000439 [identifier label].

**Paratype** (♀, NMW). CHINA: Yunnan, Gaoligongshan Nat. Res., 14–21.6.1993, E. Jendek & O. Sausa leg, AJB0000440.

##### Other material.

**CHINA**: *Yunnan*: Haba Shan, 2800–3150 m, 27.337 100.155, 8–19.VI.2004, Fouqué, R. & H, 1 ♂ 1 ♀, AJB0000462, AJB0000468 (cHay); Bichuan County, Jizu Shan, 2500–3200 m, 26–31.VII.1993, C. Holzschuh, 3 ♂, AJB0000463, AJB0000465, AJB0000469 (NMW); Jizu Shan, 2500–2700m, 25.966 100.350, 6–10.7.1994, Z. Cernin, 1 ♂ 1 ♀, AJB0000441, AJB0000464 (cHay); Weibaoshan, western slope, 25.169 100.352, 2000-2800, 25–28.VI.1992, V. Kubán, 1 ♀, AJB0000466 (NHMB).

##### Diagnosis.

Among the members of the Electus Group: head punctures clearly separated (Fig. 3E); elytra non-metallic; hind tibia entirely dark, as dark as darkened portion of femur (Fig. 3H); antennomere 6 quadrate, 7 weakly transverse (Fig. 4A); paramere slightly to distinctly longer than median lobe, with strongly attenuate apex in parameral view (Fig. 8A), in lateral view with broad lateral expansion (Fig. 8F); apex of median lobe in lateral view forming an elongate triangle (Fig. 8B); median lobe with expansion in lateral view (Fig. 8B).

##### Redescription.

Measurements ♂ (n = 5): HW/HL 1.33–1.37; PW/PL 1.28–1.32; EW/ EL 1.24–1.34; ESut/PL 0.84–0.91; PW/HW 1.09–1.13; forebody length 4.3–4.7 mm.

Measurements ♀ (n = 4): HW/HL 1.34–1.40; PW/PL 1.24–1.31; EW/ EL 1.25–1.30; ESut/PL 0.86–0.89; PW/HW 1.07–1.12; forebody length 4.6–5.1 mm.

Coloration: body black, abdominal segments sometimes slightly paler at base and apex, dorsal forebody often with faint metallic reflection; maxillary and labial palpi entirely yellowish-brown, last segment often darkened; antennomeres 1-4 or 1-5 reddish-orange with apices often darkened, 6-11 dark brown, contrasting with previous; legs bicolored: forecoxa yellow with basal fifth (males) or nearly one half dark brown (females), femur yellow and darkened apically, tibia entirely dark brown (very apex sometimes slightly paler), tarsus light brown.

Head distinctly transverse, dorsal surface with moderately dense but clearly separated and asetose punctures, frons distinctly more densely and coarsely punctate.

Pronotum distinctly transverse, posterior puncture in dorsal row occasionally doubled. Elytra weakly to distinctly transverse, shorter than pronotum at middle.

Abdomen with disc of tergites III-V distinctly, VI narrowly impunctate at middle; sternites III-V with basal line projected posteriad (IV-V weakly) at middle.

Median lobe with expansion at apical third in lateral view and narrowed to acute apex, with pair of moderately-sized lateral teeth projecting ventrad (Fig. 8B, H); apical portion of median lobe in parameral view gradually narrowed to acute apex (Fig. 8H); paramere slightly to distinctly longer than median lobe, entire but with median apical suture, distinctly dilated at apical fourth and then strongly converging to attenuate apex (Fig. 8A), in lateral view with broad lateral expansion (Fig. 8F); peg setae distributed in a marginal group and a pair of divergent and elongate median clusters that are narrowly to indistinctly separated from middle, median clusters extended only slightly basad of marginal group (Fig. 8D); apical margin of male sternites VII and VIII weakly emarginate, VIII with elongate triangular asetose area medially; male sternite IX distinctly expanded at midlength, with distinct apical emargination.

Female tergite X triangular with small rounded apex, disc not characteristically flattened.

##### Distribution.

Figure 19A. This species is distributed in the mountains of central Yunnan, China. The female paratype from Gaoligongshan may actually belong to *B. uncus* based on distribution. Until males are available from western Yunnan, the identity of this specimen must remain in doubt.

##### Bionomics.

*Bolitogyrus electus* is found at higher elevations than its closest relatives (*B. uncus*, *B. huanghaoi* and *B. confusus*) and, according to more precise labels, occurs at or above 2500 m. Specimens were collected in June and July.

##### Comments.

The identity of *Bolitogyrus electus* was until now rather confused in the literature (see discussion under *B. confusus*) and the concept herein is based on diagnostic differences consistent with the male holotype. Presently, *Bolitogyrus electus* cannot be separated from *B. uncus* unless males are dissected but it is possible that these two species are not sympatric in Yunnan Province, China.

#### Bolitogyrus uncus

Taxon classificationAnimaliaColeopteraStaphylinidae

Cai et al., 2015

Figs 8C, E, G, I , 19A (map)

Bolitogyrus uncus Cai et al., 2015: 472.

##### Type locality.

Longtanghe, Tengchong County, Yunnan, China.

##### Type material.

The holotype was not examined of this recently described species but the illustrations in the description (Cai et al. 2015) and additional photos provided by the senior author of that paper were studied.

##### Diagnosis.

Among the members of the Electus Group: head punctures clearly separated (Fig. 3E); elytra non-metallic; hind tibia entirely dark, as dark as darkened portion of femur (Fig. 3H); antennomere 6 quadrate, 7 weakly transverse (Fig. 4A); paramere slightly longer than median lobe, with weakly attenuate apex in parameral view (Fig. 8E), in lateral view with acute lateral projection (Fig. 8G); apex of median lobe in lateral view forming a relatively short triangle (Fig. 8C); median lobe with expansion in lateral view (as in Fig. 8B).

##### Distribution.

Figure 19A. Known only from one locality in western Yunnan, China.

##### Bionomics.

This species was collected at a lower elevation (ca. 2080 m) than its closest relative, *B. electus*, and may regularly occur in the different forest type. The single known specimen was collected in May.

##### Comments.

In their description of *Bolitogyrus uncus*, Cai et al. (2015) apparently compared it to the concept of *B. electus* used by Hu et al. (2011), which actually represented a different and new species (*B. confusus*). However, *B. uncus* remains a valid species based on diagnostic characters given above including the shorter apex of the median lobe and different shape of the paramere in lateral view. The differences mentioned by Cai et al. (2015) in the length of the paramere versus the median lobe (slightly longer versus approximately equal) are considered too variable based on the study of more material.

#### Bolitogyrus confusus

Taxon classificationAnimaliaColeopteraStaphylinidae

Brunke sp. n.

http://zoobank.org/3556A6A2-0615-4D7B-AB02-F0346112A8EB

Figs 8J–L , 19A (map)

Bolitogyrus electus , Hu et al. 2011 (misidentification).

##### Type locality.

Phou Pan, Hua Phan, Laos.

##### Type material.

**Holotype** (♂, NMW). NE-LAOS: prov. Hua Phan, Ban Saluei, Phou Pan, 1.-31.5.2011, 20°12'N 104°01'E, 1500–1900 m, leg. Holzschuh [printed] / Holotype ♂, *Bolitogyrus confusus* Brunke, des. A. Brunke 2017 [red printed label] / AJB0000442 [identifier label].

**Paratypes** (2 ♂ 1 ♀, NHMB, NMW, SNUC). Same data as holotype, 1 ♀, AJB0000470.

China: *Yunnan*: Pingbian County, Gejiu City, Yuping, Daweishan, 2000 m, 20.V.2005, W-X. Bi, 1 ♂, AJB0000471, SNUC.

Laos: *Xiangkhouang*: Phou Sane Mt., 30 km NE Phonsavan, 1400-1700 m, 19.630 103.335, 10-30.V.2009, D. Hauck, 1 ♂, AJB0000443, NHMB.

##### Diagnosis.

Among the members of the Electus Group: head punctures clearly separated (Fig. 3B); elytra non-metallic; hind tibia entirely dark, as dark as darkened portion of femur (Fig. 3H); antennomere 6 weakly transverse, 7 distinctly transverse (Fig. 4B); paramere slightly longer than median lobe, only moderately dilated and apex not attenuate (Fig. 8J); apex of median lobe in lateral view forming an elongate triangle (Fig. 8B); median lobe with expansion in lateral view (Fig. 8B); peg setae absent from only narrow strip along middle of paramere (Fig. 8K).

##### Description.

Measurements ♂ (n = 3): HW/HL 1.30–1.35; PW/PL 1.24–1.28; EW/ EL 1.20–1.28; ESut/PL 0.79–0.80; PW/HW 1.11–1.14; forebody length 4.0–4.6 mm.

Measurements ♀ (n = 1): HW/HL 1.38; PW/PL 1.19; EW/ EL 1.24; ESut/PL 0.79; PW/HW 1.07; forebody length 4.5 mm.

Extremely similar to *B. electus* (and *B. uncus*) and differing only in the following: antennomere 6 weakly transverse, 7 distinctly transverse (Fig. 4B), paramere much less dilated at apical third, apex not attenuate - evenly converging to apex (Fig. 8J-L).

##### Distribution.

Figure 19A. Known from southeastern Yunnan and northern Laos. A single female from northern Thailand may belong to this species.

##### Bionomics.

*Bolitogyrus confusus* has been collected from forests in May, at elevations at or less than 2000 m and has a distinctly more southern distribution than all other members of the Electus group.

##### Etymology.

The species epithet refers to the previous confusion of this species with *B. electus* and *B. uncus*.

##### Comments.

This is the taxon illustrated by Hu et al. 2011 as *B. electus*. The authors commented that the ‘apex [of the paramere] was broader’ but attributed this to intraspecific variation. Additional distinguishing characters can be found under diagnosis.

#### Bolitogyrus huanghaoi

Taxon classificationAnimaliaColeopteraStaphylinidae

Hu et al., 2011

Figs 8M–O , 19A (map)

Bolitogyrus huanghaoi Hu et al., 2011: 60.

##### Type locality.

Datang Village, Baoshan City, Tengchong County, Yunnan, China.

##### Type material.

*Bolitogyrus huanghaoi* Hu et al., 2011.

**Holotype** (♂, SNUC). CHINA: Yunnan Prov., Baoshan City, Tengchong County, Datang Village, 14-VI-2005, HUANG Hao leg. [printed] / “[Holotype]” Bolitogyrus huanghaoi, HU, LIU & LI, 2011 [red label] / AJB0000458.

##### Tentative identification.

MYANMAR: *Kachin State*: 3 km NW of 3 River Junction, 26.368 98.682, 2450 m, 3.X.2010, Michael Langer, S. Naumann and S. Löffler, 1 sex unknown [missing terminal segments], AJB0000459 (NMW).

##### Diagnosis.

Among the members of the Electus Group: head punctures clearly separated (Fig. 3E); elytra non-metallic; hind tibia entirely dark, as dark as darkened portion of femur (Fig. 3H); antennomere 6 weakly transverse, 7 distinctly transverse (4B); paramere slightly longer than median lobe, only slightly dilated and apex not attenuate (Fig. 8M); apex of median lobe in lateral view forming a more elongate triangle (Fig. 8N); median lobe without expansion in lateral view (Fig. 8N); peg setae absent from broad oval-shaped area along middle of paramere (Fig. 8O).

##### Redescription.

Measurements ♂ (n = 1): HW/HL 1.35; PW/PL 1.34; EW/ EL 1.23; ESut/PL 0.85; PW/HW 1.08; forebody length 4.9 mm.

Extremely similar to *B. electus* and differing only in the following: palpomeres and antennomeres 2-5 darker, reddish brown, antennomere 6 weakly transverse, 7 distinctly transverse (Fig. 4B), paramere much less dilated at apical third, apex not attenuate - evenly converging to apex (Fig. 8M), median lobe in lateral view without expansion (Fig. 8N), peg setae absent from oval-shaped area along middle of paramere, not appreciably grouped into marginal and medial fields (Fig. 8O).

##### Distribution.

Figure 19A. Known from a single locality in western Yunnan, China. A specimen from nearby in Myanmar is tentatively identified as this species but is missing the terminal segments of the abdomen.

##### Bionomics.

The holotype was collected at about 1600m in June.

##### Comments.

*Bolitogyrus huanghaoi* is most similar to *B. confusus* but they are distinctly allopatric and differ in the arrangement of peg setae of the paramere.

#### Bolitogyrus nigropolitus

Taxon classificationAnimaliaColeopteraStaphylinidae

Smetana, 2000

Figs 8P–Q , 19B (map)

Bolitogyrus nigropolitus Smetana, 2000: 327.
Bolitogyrus nigropolitus : Cai et al. 2015.

##### Type locality.

Jianjin Shan, Tianquan County, Sichuan, China.

##### Type material.

*Bolitogyrus nigropolitus* Smetana, 2000.

**Holotype** (♂, cSch). CHINA: W-Sichuan, 1999, Ya’an Prefecture, Tianquan Co., Jiajin Shan, Tal oberh., Labahe N.R. St., 57 km W Ya’an, 30°06'N 102°25E, Streu, Rinde, Pilze, 1800 m, 12.VII., leg. M. Schülke [printed] / Sammlung M. Schulke Berlin [green label] / HOLOTYPE Bolitogyrus nigropolitus, A. Smetana, 2000 [red label] / AJB0000455 [identifier label].

##### Other material.

**CHINA**: *Sichuan*: Baoxing County, Pujigou, 2100 m, 30.6032 102.5627, 14.VIII.2003, J. Wu, 1 ♂, AJB0000456 (IZCAS); same except Fengtongzhai, 1730 m, 30.592 102.879, 30.VI.2003, H. Zhou, 1 ♀, AJB0000457 (IZCAS).

##### Diagnosis.

Among the members of the Electus Group: head punctures clearly separated (Fig. 8E); elytra non-metallic; hind tibia with at least distal half distinctly lighter than apex of femur (Fig. 8G); median lobe in lateral view with large expansion (Fig. 8P, arrow); paramere distinctly shorter than median lobe, distinctly dilated in apical third (Fig. 8Q); peg setae absent from narrow median area of paramere, pair of median clusters extended distinctly basad of marginal group (Fig. 8Q).

##### Redescription.

Measurements ♂ (n = 2): HW/HL 1.28–1.31; PW/PL 1.25–1.31; EW/ EL 1.25–1.29; ESut/PL 0.86–0.88; PW/HW 1.11–1.15; forebody length 4.5–4.6 mm.

Measurements ♀ (n = 1): HW/HL 1.26; PW/PL 1.23; EW/ EL 1.26; ESut/PL 0.83; PW/HW 1.16; forebody length 4.8 mm.

Extremely similar to *B. electus* and differing only in the following: antennomeres I-VI paler than following segments; hind tibia brownish to brownish-yellow, paler than apical darkened area of femur, especially lateral face (Fig. 8G); head slightly less transverse; sternites III-V with basal line projected sharply posteriad at middle; median lobe in lateral view with much larger expansion at apical third (Fig. 8P), with lateral teeth smaller; paramere distinctly shorter than median lobe, slightly less dilated, apex slightly attenuate (Fig. 8Q); peg setae with pair of median clusters extended distinctly basad of marginal group (Fig. 8Q); female tergite X with acute, pointed apex.

##### Distribution.

Figure 19B. Known from three localities within a small area of central Sichuan, China.

##### Bionomics.

Collected in June-August at elevations ranging from 1730-2100 m.

##### Comments.

*Bolitogyrus nigropolitus* is easily distinguished from its closest relatives by the paler antennae and legs.

#### Bolitogyrus metallicus

Taxon classificationAnimaliaColeopteraStaphylinidae

Cai et al., 2015

Figs 9L–O , 19B (map)

Bolitogyrus metallicus Cai et al., 2015: 466.

##### Type locality.

Shennongjia Nature Reserve, Pingqian, Hubei, China.

##### Type material.

The type series of this recently well illustrated (Cai et al. 2015) species was not examined but the illustrations in the description (Cai et al. 2015) and additional photos provided by the senior author of that paper were studied.

##### Diagnosis.

Among the members of the Electus Group: many head punctures confluent (Fig. 8F); elytra not metallic blue-green; median lobe in lateral view with only minute lateral teeth, with only slight subapical expansion (Fig. 9L, arrows); paramere distinctly longer than median lobe, weakly constricted in apical fourth (Fig. 9M), with peg setae at middle, on broad, medial ridge (Fig. 9O) and extended basad of marginal group (Fig. 9N).

##### Distribution.

Figure 19B. Known from a single locality in northwestern Hubei, China and is the easternmost described species of the Electus group.

##### Bionomics.

The holotype was collected in September but nothing more is known.

##### Comments.

*Bolitogyrus metallicus* is most similar to *B. nigerrimus* from Guizhou, China and can only be distinguished from it by characters on the aedeagus including the basally extended peg setae of the paramere, median ridge of the paramere and narrower apex of the median lobe in lateral view.

#### Bolitogyrus nigerrimus

Taxon classificationAnimaliaColeopteraStaphylinidae

Yuan et al., 2007

Figs 3F–G , 9I–K , 19B (map)

Bolitogyrus nigerrimus Yuan et al., 2007: 150.

##### Type locality.

Lianhuaping, Leigong Shan, Guizhou, China.

##### Type material.

*Bolitogyrus nigerrimus* Yuan et al., 2007.

**Holotype** (♂, SNUC). Lianhuaping, Leigong Mt., Guizhou Prov., alt 1450-1500 m, 15-IX-2005, Zhu li-long leg. [printed] / “[HOLOTYPE]” Bolitogyrus nigerrimus, Yuan, Zhou, Li, & Hayashi, 2007, SHNU Collections [red label] / AJB0000460 [identifier label].

**Paratype** (♀, SNUC). Same data as holotype, AJB0000461.

##### Diagnosis.

Among the members of the Electus Group: many head punctures confluent (Fig. 3F); elytra not metallic blue-green; median lobe in lateral view with distinct but small lateral teeth, with small subapical expansion (Fig. 9I); paramere distinctly longer than median lobe, weakly constricted in apical fourth (Fig. 9J), with peg setae at middle, not elevated on ridge, medial group not extended basad of marginal group (Fig. 9K).

##### Redescription.

Measurements ♂ (n = 1): HW/HL 1.27; PW/PL 1.25; EW/ EL 1.23; ESut/PL 0.89; PW/HW 1.10; forebody length 4.5 mm.

Measurements ♀ (n = 1): HW/HL 1.29; PW/PL 1.28; EW/ EL 1.23; ESut/PL 0.89; PW/HW 1.13; forebody length 5.0 mm.

Similar to *B. electus* and differing only in the following: palpi entirely pale; tibia with distal half yellowish-brown, paler than dark apical portion of femur (Fig. 3G); head slightly less transverse, dorsal surface with punctures more deeply impressed and often confluent (Fig. 3F); pronotum with sides more strongly explanate; sternite V with basal line weakly (male) or not projected posteriad (female) at middle; median lobe in lateral view with small expansion in apical fourth, with small pair of lateral teeth (Fig. 9I); paramere distinctly longer than median lobe, slightly constricted in apical fourth, apex not attenuate (Fig. 9J); peg setae in medial group not extended basad of marginal group, both groups becoming indistinguishable at apex, medial groups not divergent and situated on midline (Fig. 9K); male sternite IX not distinctly widened at midlength, markedly more elongate; female tergite X shorter, with slightly broader apex.

##### Distribution.

Figure 19B. Known from a single locality in Guizhou, China.

##### Bionomics.

The type series was collected in September at an elevation of 1450–1500 m.

##### Comments.

*Bolitogyrus nigerrimus* is most similar to *B. metallicus* and can only be distinguished by characters on the aedeagus including the median peg setae, which are not on a ridge or extended basad, the larger lateral teeth of the paramere and larger subapical expansion of the median lobe in lateral view. The paramere was drawn in the original description (Yuan et al. 2007) with more than two macrosetae on the lateral margin when, in fact, there are only the usual two.

#### Bolitogyrus cyanipennis

Taxon classificationAnimaliaColeopteraStaphylinidae

(Zheng, 1988)

Figs 9A–D , 19B (map)

Cyrtothorax cyanipennis Zheng, 1988: 306.
Bolitogyrus cyanipennis : Cai et al. 2015.

##### Type locality.

Wolong National Nature Reserve, Sichuan, China.

##### Type material.

The type series of this recently well-illustrated (Cai et al. 2015) species was not examined.

##### Diagnosis.

Among the members of the Electus Group: many head punctures confluent (Fig. 3F); elytra bright metallic blue-green; paramere only weakly constricted in basal third (9A), median lobe not or barely visible in parameral view (Fig. 9A); peg setae with median group not extended basad of marginal group (Fig. 9C); apex of median lobe in lateral view forming an elongate triangle (Fig. 9B), in parameral view forming an approximately 90-degree angle (Fig. 9D).

##### Distribution.

Figure 19B. Known only from the type locality in central Sichuan, China.

##### Bionomics.

A recently collected specimen was found in August at an unknown elevation.

##### Comments.

*Bolitogyrus cyanipennis* cannot be externally distinguished from the allopatric *B. kitawakii* but differs by the differently shaped paramere, arrangement of peg setae and longer apex of the median lobe.

#### Bolitogyrus kitawakii

Taxon classificationAnimaliaColeopteraStaphylinidae

Smetana & Zheng, 2000

Figs 1B , 3E–H , 19B (map)

Cyrtothorax kitawakii Smetana & Zheng, 2000a: 56.
Bolitogyrus kitawakii : Hu et al. 2011.
Bolitogyrus kitawakii : Cai et al. 2015.

##### Type locality.

Bashan, Dabashan, Chengkou Xian, Chongqing, China.

##### Type material.

The type series of this recently well illustrated (Cai et al. 2015) species was not examined.

##### Other material.

**CHINA**: *Shaanxi*: Qinling mountains, Xunyangba env., 1200 m, 20.V.-10.VI.2000, 4 ♂, 6 ♀ (cHay), AJB0000444, AJB0000445, AJB0000446, AJB0000447, AJB0000448, AJB0000449, AJB0000450, AJB0000451, AJB0000452, AJB0000453. *Sichuan*: Nanjiang County, Micang Shan, Bazhong City, 1798 m, 32.664 107.029, 27-28.IV.2008, H. Huang & W. Xu, 1 ♂, AJB0000454 (SNUC).

##### Diagnosis.

Among the members of the Electus Group: many head punctures confluent (Fig. 3F); elytra bright metallic blue-green; paramere distinctly constricted in basal third, median lobe clearly visible in parameral view (Fig. 9E); peg setae with median group extended basad of marginal group (Fig. 9G); apex of median lobe in lateral view forming a shorter triangle (Fig. 9F), in parameral view forming an obtuse angle (Fig. 9H).

##### Redescription.

Measurements ♂ (n = 5): HW/HL 1.28–1.31; PW/PL 1.25–1.32; EW/ EL 1.21–1.34; ESut/PL 0.91–0.94; PW/HW 1.08–1.12; forebody length 4.4–4.8 mm.

Measurements ♀ (n = 5): HW/HL 1.28–1.33; PW/PL 1.27–1.33; EW/ EL 1.29–1.36; ESut/PL 0.88–0.92; PW/HW 1.08–1.13; forebody length 4.7–4.8 mm.

Similar to *B. electus* and differing only in the following: body bicolored, head dark with moderate metallic greenish-bronze reflection, pronotum entirely pale, reddish-orange, elytra dark, with bright metallic green to blue reflection, abdominal segments III-V entirely reddish-orange, segment VI reddish-orange with narrow part of apex dark, segments VII-VIII entirely dark; palpi entirely pale; antennomeres slightly but successively darker; forecoxae and tibia entirely yellowish-brown; head slightly less transverse; elytra very slightly longer than pronotum at middle; dorsal surface with punctures more deeply impressed and often confluent (Fig. 3F); pronotum with sides more strongly explanate; sternites III-V with basal line sharply (III-IV) or weakly (V) projected posteriad at middle; median lobe in lateral view without expansion, with slight expansion, with moderately-sized pair of lateral teeth (Fig. 9F); median lobe in parameral view with obtuse apex (Fig. 9H); paramere distinctly longer than median lobe, slightly more constricted in basal third (Fig. 9E); male sternite IX not distinctly widened at midlength, markedly more elongate; female tergite X shorter, with slightly broader apex.

##### Distribution.

Figure 19B. Known from Chongqing, Sichuan, and Shaanxi provinces of China.

##### Bionomics.

Specimens have been collected at elevations ranging from 1200-1900 m during April-June and once in August. *Bolitogyrus kitawakii* (*B. cyanipennis* probably similar) is unique within the genus for its occurrence in warm-temperate forests that experience coldest monthly mean temperatures of below freezing (-1°C) (Brunke et al. *in prep*).

##### Comments.

The Shaanxi locality reported here is slightly north of the northernmost record of the genus (also for this species) (Cai et al. 2015). *Bolitogyrus kitawakii* cannot be externally distinguished from the allopatric *B. cyanipennis* but differs by the differently shaped paramere, arrangement of peg setae and shorter apex of the median lobe.

### Undescribed species of the Electus Group

One female similar to *B. nigerrimus* was examined from Huangganshan (1800–2050 m), in the Wuyi mountains of Jiangxi province, China (FMNH). This is the only record of the Electus Group from this far east and undoubtedly represents a new species.

### Caesareus Group

The Caesareus Group (*B. caesareus*, *B. temburong*, *B. proximus*, *B. rufipennis*) consists of relatively large species that occur in the Sundaland region of southeast Asia and possess the following combination of characters: prosternum without longitudinal ridge; pronotal margin greatly expanded, at its widest point, more than four lateral puncture widths wide; median lobe of aedeagus usually with a single or pair of median teeth (Fig. 10E) but always without pairs of subapical or basal teeth that occur in members of the similar Carnifex Group; female tergite VIII lacking median notch. All members of this group, at present, also have an orange marking on the frons, which serves to distinguished them externally from members of the Carnifex Group.

#### Bolitogyrus caesareus

Taxon classificationAnimaliaColeopteraStaphylinidae

(Bernhauer, 1915)

Figs 1C , 3A , 4C , 10A–C , 19C (map)

Cyrtothorax caesareus Bernhaeur, 1915: 146.
Cyrtothorax borneensis Cameron, 1942: 138, **syn. n.**

##### Type locality.

Mt. Matang, Sarawak, Borneo, Malaysia.

##### Type material.

*Cyrtothorax caesareus* Bernhauer, 1915.

**Holotype** (♂, FMNH) [dermestid damage]. Borneo. Matang, 26.XII.1913, Moulton, Sarawak Museum [handwritten] / 18 [handwritten] / Cyrtothorax caesareus Brnh, Typus unic [handwritten] / Chicago NHMus, M. Bernhauer Collection [printed] / Holotype ♂, *Cyrtothorax caesareus* Bernhauer, 1915, det. A. Brunke 2017 [red printed label] / AJB0000396 [identifier label].

##### Type material.

*Cyrtothorax borneensis* Cameron, 1942, syn. n.

**Type locality.** Martapura, South Kalimantan, Borneo, Indonesia.

**Holotype** (♂, BMNH): Type [circle label with red border] / Martapura, S.E. Borneo, Doherty, 1891 [typed label] / C. borneensis TYPE Cam. [handwritten] / M. Cameron Bequest. B.M. 1955–147. [printed] / Holotype ♂, *Cyrtothorax borneensis* Cameron, 1942, det. A. Brunke 2017 [red printed label] / *Bolitogyrus caesareus* Bernhauer, det. A. Brunke 2017 [white printed label] / AJB0000398 [identifier label].

Cameron (1942) stated that his specimen was a female but it is actually a male with an aedeagus not appreciably different from that of the holotype of *B. caesareus*, and well within the range of variation seen by the present author for this taxon. Diagnostic characters given by Cameron included antennal and abdominal coloration but these are variable within *B. caesareus* and both extremes were observed at the same collection site (*e.g.*, Danum valley).

##### Other material.

**BRUNEI**: *Temburong*: Kuala Belalong FSC, 260 m, 4.539 115.156, Dipterocarp forest, flight intercept trap, 8.II.1992, N. Mawdsley, 1 ♀, AJB0000481, (BMNH); same except II-III.1992, 1 ♀, AJB0000482 (BMNH).

**MALAYSIA**: *Johor*: Endau-Rompin National Park, Pulau Jasin, 50–400 m, 2.516 103.349, 19.III.1998, Dembicky & Pacholatko, 1 ♂, AJB0000480 (NHMB); *Pahang*: Gunung Benom, Lata Jarum (20 km NE Raub), 350–550 m, 19–22.II.1995, M. Strba & R. Hergovits, 1 ♀, AJB0000483 (NMW); Lata Lembik, 30 km NE Raub, 200–400 m, 22.IV-V.2002, E. Jendek & O. Sausa, 1 ♀, AJB0000486 (cSHI); Taman Negara, Tahan tr. [trail], 90–130 m, primary forest, 11.III.1993, Löbl & Calame, 1 ♂ 1♀, AJB0000397, AJB0000485 (MHNG). *Sabah*: Danum Valley, B.R.I., flight intercept trap, 14–16.II.2007, G. de Rougemont, 2 ♂, AJB0000473, AJB0000478 (cRou); Lahad Datu, Ulu Segama, Forest Reserve, Danum Valley F.C., 4°57.9'N 117°48.1'E, 200 m, 1° forest, flight intercept trap, 1.XI.2005, Mann, Slade and Villaneuva, 1 ♂ 3 ♀, AJB0000474, AJB0000475, AJB0000477, AJB0000479 (OUMNH); Ranau, Poring Hot Spring and Nature Reserve, 26.X.1990, G. de Rougemont, 1 ♀, AJB0000484 (cRou); Sandakan Division, Maliau Basin Conservation Area, trail to OG2, 287 m, old growth forest, flight intercept trap, 4.745 116.969, 1 ♀, AJB0000476, Mann (OUMNH).

##### Diagnosis.

Among the members of the Caesareus Group, the yellow-ringed black spot near the humerus of each elytron is unique to *B. caesareus* (Fig. 1C). At present, it cannot be confused with any other species of *Bolitogyrus*.

##### Redescription.

Measurements ♂ (n = 5): HW/HL 1.38–1.44; PW/PL 1.29–1.46; EW/ EL 1.15–1.28; ESut/PL 0.89–0.95; PW/HW 0.96–1.07; forebody length 5.5–6.0 mm.

Measurements ♀ (n = 5): HW/HL 1.36–1.41; PW/PL 1.25–1.36; EW/ EL 1.17–1.25; ESut/PL 0.93–0.96; PW/HW 0.97–1.02; forebody length 5.2–6.4 mm.

Coloration: body yellowish-red; head black except middle third of frons; elytra with distinct black spot margined with yellow, spot about one third the length of elytra; abdominal tergites III (entirely), IV (basally and medioapically), VI (entirely except very base), VII (entirely except for pale apical one fifth) and VIII (entirely) black; antennomere 1 yellowish except for occasionally darkened apex, 2 reddish with dark apical half, 3-7 dark brown, apical four or apical three (most common) pale yellow to almost white, apical segment asymmetrically dark on one specimen, a few specimens seen with an antennomere half dark and half pale yellow; palpi entirely yellow to dark orange, apices sometimes darkened.

Head distinctly transverse, dorsal surface with moderately dense but clearly separated, asetose punctures, frons nearly impunctate. Antennomeres 6-10 transverse and asymmetrical.

Pronotum variable in shape but always distinctly transverse, convex, pronotal margin greatly expanded to variable degree. Elytra weakly to moderately transverse depending on the degree of lateral dilation, slightly shorter than pronotum at middle, in addition to usual macrosetal rows on disc, scattered punctures bearing setae, nearly all punctures setose on epipleuron of elytron; elytral disc bearing yellow margin of humeral spot raised and impunctate.

Abdomen with disc of tergites III-V distinctly, VI narrowly or not impunctate at middle; sternites III-IV with basal line distinctly, V slightly projected posteriad at middle.

Median lobe in lateral view gradually narrowed toward distinctly to slightly hooked apex, slightly constricted at apical third, without median tooth (Fig. 10B); median lobe in parameral view with apical half spoon shaped, apex slightly acute and pointed (Fig. 10A); paramere slightly to very slightly shorter than median lobe, constricted in basal third, variably dilated at about midlength, peg setae distributed in marginal group, about 2-4 peg setae wide, sometimes forming a median group in basal half (Fig. 10C); apical margin of male sternite VII with very slight emargination, male sternite VIII with shallow but distinct emargination and broad triangular, flattened, glabrous area medially; male sternite IX distinctly expanded at midlength, with distinct emargination, disc mostly glabrous except for conspicuous, divergent pair of rows of long setae.

Female tergite X elongate triangular, constricted at about midlength, with elongate raised discal area that is strongly impressed longitudinally.

##### Distribution.

Figure 19C. Distributed on both Borneo and mainland Malaysia. No males were available from Brunei and these are only tentatively identified as this species.

##### Bionomics.

*Bolitogyrus caesareus* is an inhabitant of lowland, often primary, rainforest, up to an elevation of about 550 m. Specimens were collected February to April and September-October.

##### Comments.

No consistent differences could be found between specimens from mainland Asia and Borneo. Bernhauer (1915) speculated that this species might be termitophilous as it was sent to him along with many other specimens of species known to be termite guests. Similarly, one recent specimen was seen with a highly modified, likely inquiline, aleocharine rove beetle attached to its midleg. As in other staphylinines that are confirmed to be associated with termites (e.g., *Taxiplagus*), the apical antennomeres of *B. caesareus* and its closest relatives are asymmetrical and slightly expanded, possibly to find social insects via airborne pheromones. While this species is unlikely to be a truly integrated ‘guest’ of termites, it is quite possibly a predator of wood-nesting termites and may become attracted to nests that have been physically compromised.

#### Bolitogyrus proximus

Taxon classificationAnimaliaColeopteraStaphylinidae

(Cameron, 1942)

Fig. 1D , 3B , 10D–G , 19D (map)

Cyrtothorax proximus Cameron, 1942: 138

##### Type locality.

Martapura, South Kalimantan, Borneo, Indonesia.

##### Type material.

*Cyrtothorax proximus* Cameron, 1942.

**Holotype** (♂, BMNH). Type [circle label with red border] / Martapura, S.E.

Borneo, Doherty, 1891 [printed] / C. proximus TYPE Cam. [handwritten] / M. Cameron. Bequest., B.M. 1955–147 [printed] / Holotype ♂, *Cyrtothorax proximus* Cameron, 1942, det. A. Brunke 2017 [red printed label] / AJB0000399 [identifier label].

Cameron (1942) stated that his specimen was a female, though it is a male.

##### Other material.

**BRUNEI**: *Temburong*: Kuala Belalong FSC, 4°34'N 115°7'E, malaise GM3, 18.V.1991, N. Mawdsley, 1 ♀, AJB0000498 (BMNH).

**MALAYSIA**: *Sabah*: Batu Punggul Resort, environs of, vegetation and forest floor litter around large trees near river, 24.VI-1.VII.1996, 1 ♀, AJB0000493 (MHNG); same except, intercept trap, 23-V-2001, J. Kociam, 1 ♀, AJB0000497 (NMW); Danum Valley, B.R.I., flight intercept trap, 14-16.II.2007, G. de Rougemont, 1♂, AJB0000488 (cRou); same except, 1.VI.1999, G. Mendel, 1 ♀, AJB0000492 (BMNH); Lahad Datu, Ulu Segama, Forest Reserve, Danum Valley F.C., 4°57.9'N 117°48.1'E, 200 m, 1° forest, flight intercept trap, 4.IV.2005, E. Slade and Villaneuva, 1 ♂, AJB0000489 (OUMNH); same except 1.XI.2005, 1 ♂, AJB0000490 (OUMNH); Penanpang, flight intercept trap, 11-15.I.2008, Y. Shibata, 1 ♀, AJB000091 (cShi); Sepilok Nature Resort, in fungi on log, 13.II.2007, G. de Rougemont, 1 ♀, AJB0000487 (cRou); Tawau Hills Park, 7-9.VI-1998, P. Hlavac, 1 ♂, AJB0000400 (NMW); *Sarawak*: Gunung Mulu National Park, near base camp, 50-100 m, alluvial forest, malaise trap, V-VIII.1978, P. Hammond and J. E. Marshall, 1 ♀, AJB0000496 (BMNH).

**INDONESIA**: *Central Kalimantan*: confluence of Busang and Rekut rivers, flight intercept trap, VIII.2001, Brendell and Mendel, 1 ♂ 1 ♀, AJB0000494, AJB0000495 (BMNH); *East Kalimantan*: Paiau River, Mjöberg, 1 ♂, NHRS-JLKB-000021956 (NHRS).

##### Diagnosis.

Among the members of the Caesareus Group: pronotum not entirely pale; elytra without dark spot; first two visible abdominal segments with distinct darkened area (Fig. 1D); median lobe in parameral view converging to acute apex (Fig. 10D).

##### Redescription.

Measurements ♂ (n = 5): HW/HL 1.34–1.42; PW/PL 1.39–1.46; EW/ EL 1.21–1.25; ESut/PL 0.86–0.90; PW/HW 1.10–1.13; forebody length 6.1–6.5 mm.

##### Measurements

♀ (n = 5). HW/HL 1.39–1.48; PW/PL 1.32–1.41; EW/ EL 1.22–1.24; ESut/PL 0.88–0.94; PW/HW 1.06–1.09; forebody length 5.9–6.5 mm.

Similar to *B. caesareus* but differing only in the following: antennae with apical 1 or 2 segments distinctly paler, first segment entirely yellow; orange area on frons distinctly larger, reaching up to half the length of eyes; scutellum varying from entirely reddish to basal two-thirds dark brown; palpi with last segment darkened; pronotum not entirely pale and always dark medially with pale expanded margin: sometimes with a spot in each orange lateral area, sometimes pronotum with only anterior angles paler, or pronotum entirely dark; antennomeres 7-10 transverse and asymmetrical; elytra slightly shorter relative to pronotum at middle; elytral disc with small, variably-shaped, elevated and impunctate yellow spot; pronotum wider relative to head; pronotal margin distinctly more expanded; elytral disc with setose punctures only in usual rows; sternites III-V with basal line distinctly projected posteriad at middle; median lobe in lateral view slightly constricted just before apex, this part slightly deflected dorsad, with tooth basad of constriction, this tooth arising from middle of subapex, not apical carina (Fig. 10F); median lobe in parameral view sometimes with slight ridge connecting apex with tooth (Fig. 10E); paramere slightly longer than median lobe, much more strongly constricted at basal third and more strongly expanded about midlength, peg setae similar but more clearly separated into marginal and medial groups, sometimes connecting to form a pair of ovoid shapes (Fig. 10G); male sternite VII additionally with glabrous triangular area medioapically; male sternite IX with regular setation, without conspicuous rows of long setae; female tergite X triangular with acute apex, with raised, flat discal area of approximately same shape.

##### Distribution.

Figure 19D. Endemic to the island of Borneo. The single specimen from Brunei is a female but is tentatively assigned to this species as it was collected at a lower elevation than those of the externally indistinguishable *B. temburong*.

##### Bionomics.

Like the often co-collected *B. caesareus*, *B. proximus* is a species of lowland rainforests in Borneo. Several specimens indicate large trees or primary rainforest on the labels. Specimens have been collected during the months of January-February, April-August, and November, in flight intercept traps and from litter at elevations ranging from 0-200 m. *Bolitogyrus proximus* has been frequently collected along larger rivers, while its cryptic sister species, *B. temburong* occurs in lower montane forests (>400 m).

##### Comments.

Although externally indistinguishable from *B. temburong*, *B. proximus* differs dramatically in the shape of the paramere and the median lobe in lateral view; it may also be micro-allopatric with it at a lower elevation.

#### Bolitogyrus temburong

Taxon classificationAnimaliaColeopteraStaphylinidae

Brunke sp. n.

http://zoobank.org/D28FBB73-6436-4F61-91BF-B300A901764C

Figs 10H–J , 20A (map)

##### Type material.

**Holotype** (♂, BMNH): BRUNEI, E115°7’ N4°34’, Kuala Belalong FSC, Dipterocarp forest, BM(NH) 1991-173 [printed] / Ground Malaise, 20A, 770m alt, 7.ii.92, N. Mawdaley NM302 [printed] / Holotype ♂, *Cyrtothorax temburong* Brunke, des. A. Brunke 2017 [red printed label] / AJB0000401 [identifier label].

**Paratypes** (1 ♂ 1 ♀, BMNH): same as holotype except, 440 m, ground malaise #13, 8.II.1992, AJB0000499; same as holotype except, 610 m, ground malaise #18, AJB0000500.

##### Diagnosis.

Among the members of the Caesareus Group: pronotum not entirely pale; elytra without dark spot (Fig. 1D); first two visible abdominal segments with distinct darkened area (Fig. 1D); median lobe in parameral view dilated to blunt apex (Fig. 10H).

##### Description.

Measurements ♂ (n = 2): HW/HL 1.37–1.44; PW/PL 1.43–1.52; EW/ EL 1.29–1.31; ESut/PL 0.93–0.94; PW/HW 1.13–1.13; forebody length 5.7–6.2 mm.

Measurements ♀ (n = 1): HW/HL 1.33; PW/PL 1.64; EW/ EL 1.30; ESut/PL 0.95; PW/HW 1.16; forebody length 6.0 mm.

Almost identical to *Bolitogyrus proximus* except: pronotum entirely black or with anterior angles orange, though variation similar to *B. proximus* may occur with more specimens; elytra slightly wider; elytra slightly longer proportionally than pronotum; median lobe in lateral view with apical portion thicker and flexed ventrad, median tooth situated more distally (Fig. 10I); median lobe in parameral view widened to a rounded, obtuse apex with outline bearing median tooth (Fig. 10H); paramere with more elongate and blunt apex (Fig. 10J).

##### Distribution.

Figure 20A. Currently known only from Brunei but probably more widely distributed at medium-low elevations in Borneo.

##### Bionomics.

*Bolitogyrus temburong* has been collected in February from lower montane forests at elevations ranging from 440-770m.

##### Etymology.

The species epithet recognizes Ulu Temburong National Park in Brunei and its conservation achievements.

##### Comments.

*Bolitogyrus temburong* likely represents the first of many undescribed *Bolitogyrus* species at medium elevations in Borneo. Based on the samples seen by the author, very limited micro-fogging of fungusy logs has been conducted on Borneo.

#### Bolitogyrus rufipennis

Taxon classificationAnimaliaColeopteraStaphylinidae

(Cameron, 1937)

Figs 1E , 10K–M , 19D (map)

Cyrtothorax rufipennis Cameron, 1937: 27

##### Type locality.

Baturaden (“Batoerraden”), Mt. Slamet, Central Java.

##### Type material.

*Cyrtothorax rufipennis* Cameron, 1937.

**Lectotype** (♂, BMNH, here designated): Batoerraden, G. Slamat [=Mount Slamet], Java, F.C. Drescher, VII.1928 [printed] / M. Cameron., Bequest, B.M. 1955-147 [printed] / Lectotype ♂, *Cyrtothorax rufipennis* Cameron, 1937, des. A. Brunke 2017 [red printed label] / AJB0000402 [identifier label].

**Paralectotypes** (2 ♂, 1 ♀, BMNH, FMNH): same as lectotype, AJB0000403, AJB0000502; same as lectotype except, 18.I.1929, AJB0000501.

In his description of *B. rufipennis*, Cameron (1937) stated ambiguously that the ‘Type’ was in his collection. However, label data was given for ‘Batoerraden’ and ‘Pengalengan’, two different, distant Javanese volcanoes. Complicating matters, the specimen bearing a circular BMNH type label (the only one collected by Fruhstorfer) does not correspond with Cameron’s morphological description (black frons, only apical antennomere paler) and is instead a female closely related to *B. doesburgi* (Scheerpeltz). In order to stabilize the taxonomic concept of *B. rufipennis*, a different, male, specimen (from “Batoerraden”) that matches the original description (orange frons, apical two antennomeres paler) is here designated as a lectotype. The female from Pengalengan is excluded from the paralectotypes.

##### Diagnosis.

Among the members of the Caesareus Group: pronotum entirely pale; elytra without dark spot; first two visible abdominal segments without distinct darkened area (Fig. 1E); median lobe with a pair of median teeth at subapex.

##### Redescription.

Measurements ♂ (n = 3): HW/HL 1.35–1.35; PW/PL 1.31–1.35; EW/ EL 1.18–1.20; ESut/PL 0.76–0.79; PW/HW 1.01–1.04; forebody length 6.4–6.7 mm.

Measurements ♀ (n = 1): HW/HL 1.31; PW/PL 1.20; EW/ EL 1.21; ESut/PL 0.76; PW/HW 1.0; forebody length 6.4 mm.

Similar to *B. caesareus* but differing only in the following: antennae with apical 2 segments distinctly paler, first segment entirely yellow; palpi with last segment darkened; abdominal tergites III-V without clear dark markings; antennomeres 7-10 transverse and asymmetrical; head slightly less transverse; pronotum slightly less transverse; elytra distinctly shorter proportional to pronotum at middle; forebody slightly longer; elytral disc with small, variably-shaped, elevated and impunctate yellow spot; elytral disc with setose punctures only in usual rows; median lobe in lateral view angularly truncate with sharp apex, with pair of median teeth basad of truncate face (one visible in lateral view), this tooth arising from middle of subapex, not apical carina (Fig. 10L); median lobe in parameral view more strongly dilated subapically (Fig. 10K); paramere slightly longer than median lobe, peg setae similar but more restricted to marginal area and leaving a wider median space empty (Fig. 10 M); male sternite IX with regular setation, without conspicuous rows of long setae; female unknown.

##### Distribution.

Figure 19D. Endemic to Java and possibly to Mt. Slamet.

##### Bionomics.

No additional data accompanies the specimens.

##### Comments.

Several female specimens examined are similar to *B. rufipennis* but may represent additional species (see ‘Undescribed Species’, below).

### Undescribed species of the Caesareus Group.

The true diversity of the Caesareus Group appears to be very poorly known based on the number of female specimens that likely represent additional species. Three females from Borneo, consisting of 2–3 species (NMW, BMNH, cShi), are similar to but much larger than *B. proximus*, possess differently shaped yellow spots on the elytral disc and differ in general coloration. One female specimen, from peninsular Malaysia (600 m) and nearly identical to *B. proximus*, may represent another undescribed species as it differs in the shape of female tergite X. An additional female specimen from peninsular Malaysia was examined that closely resembles *B. rufipennis* (known only from Java) yet has an entirely dark pronotal disc. Another single female bearing only the label data ‘Java’ (BMNH) closely resembles the previous specimen.

### Carnifex Group

The Carnifex Group (*B. carnifex*, *B. elegantulus*, *B. magnimaculosus*, *B. nokrek*, *B. pederseni*, *B. phukhieo*, *B. vietnamensis*) includes the largest species of the genus. The group can be recognized by the following combination of characters: pronotal margin greatly expanded, at its widest point, more than four lateral puncture widths wide (Fig. 4C); frons not contrasting in color with rest of head (Fig. 1G); median lobe with pair of basal teeth (Fig. 11A), sometimes with pair of similar, subapical teeth (Fig. 11B); female tergite 8 always with emargination.

#### Bolitogyrus carnifex

Taxon classificationAnimaliaColeopteraStaphylinidae

(Fauvel, 1878)

**Figure 5.**
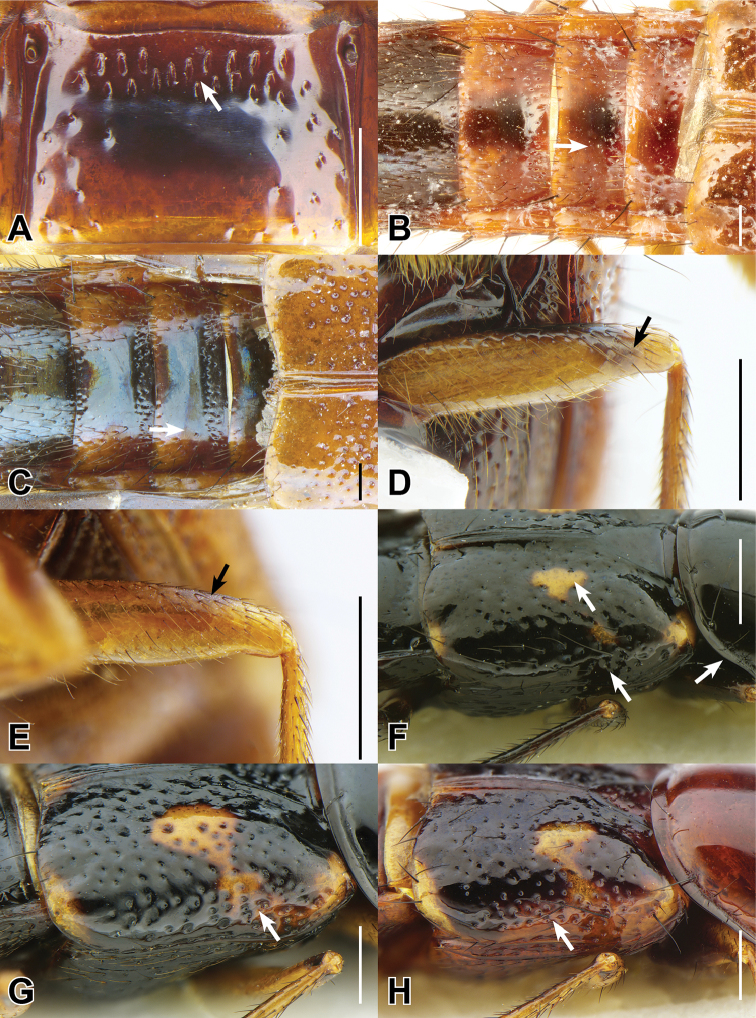
Abdominal tergite III: *Bolitogyrus sepilok* Brunke (**A**). Dorsal abdomen: *B. elegantulus* Yuan et al. (**B**) and *B. carnifex* (Fauvel) (**C**). Anterior face of mid-femur: *B. elegantulus* (**D**) and *B. phukhieo* Brunke (**E**). Lateral view of elytron: *B. ornatipennis* (Wendeler) (**F**), *B. pictus* Yuan et al. (**G**) and *B. solodovnikovi* Brunke (**H**). Scale bars: 0.25 mm (**A**), 0.5 mm (**B–H**).

Figs 5C , 11A–C , 20B (map)

Cyrtothorax carnifex Fauvel, 1878: 166.

##### Type locality.

‘Cambodia’

##### Type material.

*Cyrtothorax carnifex* Fauvel, 1878.

**Holotype** (♂, BMNH): ♂, Cyrtothorax carnifex. Type., Cambodia, Mouhot [written on specimen card] / Type [circular type label with red border] / Sharp Coll, 1905-313 [printed] / Cyrtothorax carnifex Fvl. [handwritten] / Holotype ♂, *Cyrtothorax carnifex* Fauvel, 1878, det. A. Brunke 2017 [red printed label] / AJB0000387 [identifier label].

This specimen in the BMNH matches all information provided by Fauvel (1878), who indicated that a single male was studied from Sharp’s collection in London, and is therefore interpreted as the holotype. Another BMNH specimen, here identified as *B. elegantulus*, and bearing no labels, was studied. According to G. Rougemont (*pers. comm*.), its original labels corresponded exactly to those of the type of *B. carnifex.* Based on the fact that Fauvel studied a single specimen from Sharp’s collection in London, and the known distribution of *B. elegantulus*, the true locality data for this specimen must be considered doubtful or simply, lost. However, it is possible that Mouhot did, in fact, collect this specimen during his travels (he also explored Laos) and that Sharp received it later, and prepared it in his stereotypical way.

##### Other material.

**VIETNAM**: *Dong Nai*: Cat Tien N.P., 11.433 107.433, 6-16.VII.2012, malaise trap, leg. J. Constant and J. Bresseel, 1 ♂, AJB0000588 (IRSNB).

##### Diagnosis.

Within the Carnifex Group: elytral disc entirely reddish (Fig. 1F); abdominal tergites III-V with relatively wide dark markings at middle (Fig. 5C); peg setae arranged in both marginal and medial groups (Fig. 11C); subapical teeth present but not on a carina (Fig. 11B); antennomeres 8-10 transverse; in parameral view, paired basal teeth appearing near margins of median lobe (Fig. 11A).

##### Redescription.

Measurements ♂ (n = 2): HW/HL 1.39-1.40; PW/PL 1.37-1.39; EW/ EL 1.25-1.26; ESut/PL 0.83-0.84; PW/HW 1.04-1.05; forebody length 6.4-6.6 mm.

Coloration: head entirely dark; pronotum reddish with median dark, irregular spot; elytra and scutellum reddish, disc with slightly raised yellow v-shaped marking; abdominal tergites III-V reddish with central dark marking slightly more than one-third to two-thirds the tergal width, VI dark, VII-VIII dark with paler base and apex; antennomere 1 yellow, 2-5 reddish, 6-10 dark brown, 11 yellow-brown; palpi brownish orange, apical segment darkened; legs yellowish brown, dorsal surface but not lateral face of mid-femur with darker brown (Fig. 5E) (on holotype), one nontype male with slight ventral darkening, hind femur with subapical band of dark brown; outer faces of tibia paler.

Head distinctly transverse, dorsal surface with moderately dense, clearly separated asetose punctures, frons with only scattered punctures and deep Y-shaped impression. Antennomeres 8-10 slightly transverse and asymmetrical.

Pronotum distinctly transverse, about as wide as head, convex and with very few shallow micropunctures scattered on disc, becoming more distinct on anterior angles. Elytra slightly transverse, suture distinctly shorter than pronotum at middle.

Abdomen with disc of tergites III-V distinctly impunctate; sternites III-IV with basal line distinctly, V weakly projected posteriad at middle.

Median lobe in lateral view strongly constricted in apical fourth, apical fourth deflexed ventrad, apex knob-like without distinct median tooth, with weakly formed pair of subapical teeth (Fig. 11B); median lobe with pair of basal teeth, in lateral view appearing removed from expanded ventral face (Fig. 11B), in parameral view appearing at lateral margin (Fig. 11A); median lobe in parameral view weakly expanded to apical fourth, at this level forming triangular, extremely slightly acuminate apical portion, apex acute but rounded (Fig. 11A); paramere slightly constricted in basal third, weakly dilated in apical third and narrowed evenly to apex (Fig. 11C); peg setae with marginal group, median group disorganized and connected in several places to marginal group (Fig. 11C); male sternite VIII with shallow emargination and triangular glabrous area medially; male sternite IX moderately expanded at midlength, with distinct, deep emargination.

Female unknown.

##### Distribution.

Figure 20B. Known from southern Vietnam and an unknown locality in Cambodia.

##### Bionomics.

One specimen has been collected in July.

##### Comments.

*Bolitogyrus carnifex* is probably most closely related to allopatric *B. pederseni* and *B. vietnamensis* based on the wide abdominal markings and the laterally placed basal teeth of the median lobe. It can be distinguished from these two species by the weakly formed subapical teeth of the median lobe that do not form carinae.

#### Bolitogyrus pederseni

Taxon classificationAnimaliaColeopteraStaphylinidae

Brunke sp. n.

http://zoobank.org/03576AA6-8905-40D5-86B0-7235BF62287C

Figs 1F , 11D–F , 20B (map)

##### Type locality.

Ban Thongvay, Muang Paxong, Bolaven Plateau, Champasak, Laos.

##### Type material.

**Holotype** (♂, ZMUC): DNA voucher [blue label] / LAOS: Champasak prov., Bolaven plat., 1000 m, 8.VI.2008, Solodovnikov and Pedersen / zmuc00046170 [identifier label] / Bolitogyrus n.sp. nr. carnifex Det. A. Brunke / HOLOTYPE *Bolitogyrus pederseni* Brunke, des. A. Brunke 2017 [red label].

**Paratype** (1 ♂ IRSNB): Coll. I.R.Sc.N.B., Cambodia, Ratanakiri prov., Veunsai, 13°59N 106°49E, 10-15.x.2007, leg. S. DeGreef and P. Naskreki / AJB0000587 [identifier label] / PARATYPE *Bolitogyrus pederseni* Brunke, des. A. Brunke 2017 [yellow label].

##### Diagnosis.

Within the Carnifex Group: elytral disc entirely reddish (Fig. 1F); abdominal tergites III-V with relatively wide dark markings at middle (Fig. 5C); peg setae arranged in both marginal and medial groups (Fig. 11F); apex of median lobe knob-like in lateral view, with prominent pair of subapical teeth each situated on longitudinal ridge (Fig. 11E); basal teeth of median lobe situated laterally (Fig. 11D).

##### Description.

Measurements ♂ (n = 2): HW/HL 1.38–1.39; PW/PL 1.40–1.42; EW/ EL 1.23–1.24; ESut/PL 0.82–0.84; PW/HW 1.13–1.14; forebody length 6.3–6.4 mm.

Extremely similar to *B. carnifex* and differing only in the following: lateral face of mid-femur with at least a faint dark band (Fig. 5D); median lobe in lateral view with prominent pair of subapical teeth each situated on longitudinal ridge forming lateral outline (Fig. 11E); median lobe in parameral view more slender, constricted weakly about midway, slightly dilated in apical third and narrowed to acuminate apex (Fig. 11D); median lobe in lateral view narrower in apical half (Fig. 11E); basal-most peg setae removed from margin (Fig. 11F).

##### Distribution.

Figure 20B. Known from the Bolaven Plateau in southern Laos and an adjacent region of northern Cambodia.

##### Bionomics.

The holotype was collected by pyrethrum fogging of fungusy logs in a disturbed primary rainforest (selective logging) at 1000 m during June. The paratype was collected in a malaise trap during October.

##### Etymology.

This species is named after Jan Pedersen (ZMUC), a friend and Coleopterist, collector of the holotype, and one who shares an interest in this genus.

##### Comments.

*Bolitogyrus pederseni* is most similar to allopatric *B. vietnamensis* from northern Vietnam but can be distinguished by the more rounded apex of the median lobe and broader paramere.

#### Bolitogyrus vietnamensis

Taxon classificationAnimaliaColeopteraStaphylinidae

(Scheerpeltz, 1974)

Figs 11G–I , 20B (map)

Cyrtothorax vietnamensis Scheerpeltz, 1974: 185

##### Type locality.

Bao Lac (town, likely in vicinity of), Cao Bang, Vietnam..

##### Type material.

*Cyrtothorax vietnamensis* Scheerpeltz, 1974.

**Holotype** (♀, NMW): ♀ [printed] / TONKIN, Bao Lac [printed] / Le Moult vend via Reinbak [printed] / Cyrtothorax vietnamensis n. spec. [handwritten] / ex. coll. Scheerpeltz [blue printed label] / ALLOTYPUS [red handwritten label] / TYPUS Cyrtothorax vietnamensis O. Scheerpeltz [red label] / vietnamensis Scheerp. [handwritten] / AJB0000404 [identifier label].

##### Other material.

**VIETNAM**: *Tuyen Quang*: NaHang Reserve, rainforest, FIT, 16-20.V.1997, S. Peck, 1 ♂, CNC655573 (CNC).

##### Diagnosis.

Within the Carnifex Group: elytral disc entirely reddish (Fig. 1F); abdominal tergites III-V with relatively wide dark markings at middle, more than 1/3 of tergal width (Fig. 5C); median lobe with basal teeth placed laterally in parameral view (Fig. 11G); paramere with slender apical portion and rows of peg setae (Fig. 11I).

##### Redescription.

Measurements ♂ (n = 1): HW/HL 1.50; PW/PL 1.37; EW/ EL 1.22; ESut/PL 0.80; PW/HW 1.12; forebody length 7.1 mm

Measurements ♀ (n = 1): HW/HL 1.48; PW/PL 1.36; EW/ EL 1.22; ESut/PL 0.79; PW/HW 1.11; forebody length 6.9 mm.

Extremely similar to *B. carnifex* and differing only in the following: head slightly more transverse; pronotum slightly wider relative to head; lateral face of midfemur with distinct dark band on holotype female (as in Fig. 5D) and only vague darkening on non-type male (as in Fig. 5E); median lobe in parameral view with narrower apical portion (Fig. 11G), in lateral view with subapical teeth on carina connected to apex, apex more angulate, less expanded near level of basal teeth (Fig. 11H); paramere much more slender apically, with peg setae arranged into rows (Fig. 11I).

##### Distribution.

Figure 20B. Currently known from two localities in northern Vietnam.

##### Bionomics.

One specimen was collected in an FIT in a rainforest during May, at a relatively low (but unspecified elevation).

##### Comments.

Scheerpeltz (1974) stated that the type of *B. vietnamensis* was completely dark on the pronotum and abdomen, while the specimen is actually bicolored on both. The reddish areas of the specimen have become darkened, likely by a killing agent. *Bolitogyrus vietnamensis* is most similar to allopatric *B. pederseni* from southern Laos and northern Cambodia but can be distinguished by the differently shaped apex of the median lobe and narrower paramere.

#### Bolitogyrus elegantulus

Taxon classificationAnimaliaColeopteraStaphylinidae

Yuan et al., 2007

Figs 5B,D , 11J–L , 20B (map)

Bolitogyrus elegantulus Yuan et al., 2007: 152.
Bolitogyrus elegantulus : Cai et al. 2015.

##### Type locality.

Manfei, Nabanhe Nature Reserve, Xishuangbanna, Yunnan, China.

##### Type material.

*Cyrtothorax elegantulus* Yuan et al., 2007.

**Holotype** (♂, SNUC): “[HOLOTYPE], Bolitogyrus elegantulus, Yuan, Zhao, Li & Hayashi, 2007, SHNU Collections [red written label] / Manfei, Nabanhe N. R., Jinghong City, Yunnan Prov., 10.I.2004, Li-Zhen Li & Liang Tang leg. [white, printed] / AJB0000389 [identifier label].

**Paratype** (♀, SNUC): same data as holotype except: 9.I.2004, AJB0000405.

##### Other material.

**LAOS**: *Oudomxay*: Ban Nam Mo, 3.IV.1918, R.V. de Salvaza, 1 ♂, AJB0000504 (BMNH); *Phongsali*: Phongsali env., 1300-1500 m, 1-15.V.2004, Lao, 1 ♂, AJB0000390 (cShi). Country unknown: 1 ♂ (BMNH) [labels lost].

##### Diagnosis.

Within the Carnifex Group: elytral disc entirely reddish (Fig. 1F); abdominal tergites III-V with relatively narrow dark markings at middle (Fig. 5B); peg setae arranged in both marginal and medial groups (Fig. 11L); basal teeth of median lobe placed medially in parameral view (Fig. 11J); apex of median lobe in parameral view evenly narrowed to apex (Fig. 11J).

##### Redescription.

Measurements ♂ (n = 4): HW/HL 1.38–1.45; PW/PL 1.31–1.41; EW/ EL 1.18–1.27; ESut/PL 0.77–0.80; PW/HW 1.08–1.14; forebody length 6.3–7.9 mm.

Measurements ♀ (n = 1): HW/HL 1.38; PW/PL 1.44; EW/ EL 1.25; ESut/PL 0.83; PW/HW 1.14; forebody length 7.3 mm.

Extremely similar to *B. carnifex* and differing only in the following: dark medial area on pronotum varying from similar to *B. carnifex* to distinctly larger; lateral face of midfemur with distinct dark band (Fig. 5D); pronotum slightly wider relative to head; median lobe in parameral view almost evenly converging to smaller apex (Fig. 11J), in lateral view, slightly more strongly constricted after basal tooth, which is distinct and appearing at ventral face (Fig. 11K); apex of median lobe in lateral view with median tooth arising from carina, apical portion only slightly deflexed ventrad (Fig. 11K); median lobe in parameral view with apical portion evenly converging to narrower apex, without pair of subapical teeth, basal teeth appearing medially (Fig. 11J); paramere with narrower and more elongate apical portion, basal constriction narrower (Fig. 11L); peg setae with marginal and medial groups more approximate and linear (Fig. 11L); female tergite VIII with narrow and deep emargination; female tergite X with obtuse but pointed apex, disc with pair of raised carinae in apical half, carinae not forming lateral margins of disc.

##### Distribution.

Figure 20B. Distributed in southern Yunnan, China and northern Laos.

##### Bionomics.

Specimens have been collected in January, April and May at elevations ranging from 810-1500 m.

##### Comments.

*Bolitogyrus elegantulus* is most similar to allopatric *B. phukhieo* from central Thailand but can be distinguished by the evenly converging apex of the median lobe in parameral view and the differently shaped paramere.

#### Bolitogyrus phukhieo

Taxon classificationAnimaliaColeopteraStaphylinidae

Brunke sp. n.

http://zoobank.org/2C4BBAD0-9F6E-4B1D-BA3B-4901BC30B464

Figs 5E , 11M–O , 20B (map)

##### Type locality.

Mon Lake, Phu Khieo Wildlife Sanctuary, Chaiyaphum, Thailand

##### Type material.

**Holotype** (♂, ZMUC): THAILAND, Chaiyaphun [sic], Phu Khieo-Bung Mon, 1000 m, pitfall trap, 25 January 1989, M. Andersen & H. Read [printed] / HOLOTYPE *Bolitogyrus phukhieo* Brunke, des. A. Brunke 2017 [red label] / AJB0000392 [identifier label].

##### Diagnosis.

Within the Carnifex Group: elytral disc entirely reddish (Fig. 1F); abdominal tergites III-V with relatively narrow dark markings at middle (Fig. 5B); peg setae arranged in both marginal and medial groups (Fig. 11O); antennomeres 8-10 quadrate; median lobe in parameral view with basal pair of teeth placed medially (Fig. 11M); apex of median lobe in parameral view acuminate (Fig. 11M).

##### Description.

Measurements ♂ (n = 1): HW/HL 1.39; PW/PL 1.47; EW/ EL 1.21; ESut/PL 0.82; PW/HW 1.15; forebody length 6.1 mm.

Extremely similar to *B. carnifex* and differing only in the following: body with paler areas lighter, more orange-yellow; pronotum with smaller spot medially, with two lateral spots; abdominal tergites with narrower central dark markings, about one quarter of the tergal width; antennomere 10 slightly paler than 9; antennomeres 8-10 quadrate; pronotum wider than head; body smaller (based on single specimen); median lobe in lateral view evenly narrowed to smaller apex, apical portion only weakly flexed ventrad, apex with distinct median tooth arising from carina, basal teeth prominent and appearing near ventral face (Fig. 11N); median lobe in parameral view with apical portion more strongly acuminate, apex narrower and pointed, basal teeth located medially (Fig. 11M); paramere less strongly dilated at apical third, apical portion less strongly converging to truncate apex (Fig. 11O); peg setae in medial group larger than those of marginal group, medial group mostly at expanded area of paramere (Fig. 11O).

##### Distribution.

Figure 20B. *Bolitogyrus phukhieo* is probably endemic to central Thailand.

##### Bionomics.

The holotype was collected in a pitfall trap in January at an elevation of 1000 m.

##### Etymology.

In recognition of Phu Khieo Wildlife Sanctuary, which encompasses the type locality: a remarkable bowl-like plateau raised out of the surrounding lowland landscape to approximately 800-1000 m. The sustainable conservation of Phu Khieo is the subject of a collaborative project between Thailand and the European Union.

##### Comments.

*Bolitogyrus phukhieo* is most similar to allopatric *B. elegantulus* from northern Laos and southern Yunnan, China but can be distinguished by the acuminate apex of the median lobe in parameral view and the differently shaped paramere.

#### Bolitogyrus magnimaculosus

Taxon classificationAnimaliaColeopteraStaphylinidae

Cai et al., 2015

Figs 12A–C , 20B (map)

Bolitogyrus magnimaculosus Cai et al., 2015: 463.

##### Type locality.

Fifth District, Ledong County, Hainan, China

##### Type material.

The type series of this recently described, and well-illustrated species was not examined (Cai et al. 2015).

##### Diagnosis.

Within the Carnifex Group: elytral disc entirely reddish (Fig. 1F); abdominal tergites III-V with relatively narrow dark markings at middle (Fig. 5B); peg setae arranged in single arcuate group, removed basally from margin (Fig. 12C). *Bolitogyrus magnimaculosus* is the only species of the Carnifex Group known from Hainan Island, China.

##### Distribution.

Figure 20B. Likely endemic to Hainan Island, China.

##### Bionomics.

This species has been collected at elevations ranging from 525-978 m, in November and December. One specimen was collected by ‘beating the shrubs’ (Cai et al. 2015) and was probably dispersing to a suitable microhabitat.

##### Comments.

*Bolitogyrus magnimaculosus* is easily identified by geography alone but is also the only species known with both subapical teeth and proximally placed basal teeth on the median lobe.

#### Bolitogyrus nokrek

Taxon classificationAnimaliaColeopteraStaphylinidae

Brunke sp. n.

http://zoobank.org/56FBD748-0551-4AF4-93CC-350E4F791131

Figs 1G , 12D–G , 20C (map)

##### Type locality.

Nokrek Biosphere Reserve, Garo Hills, Meghalaya, India.

##### Type material.

**Holotype** (♂, cHay): INDIA-NE, Meghalaya, 25°27'N 90°19'E, NOKREK N.P., 1400m, Garo Hills, 26.iv.1999, Z. Koštál leg. [printed] / *Bolitogyrus* sp. det. Y. Hayashi 2012 [printed] / HOLOTYPE *Bolitogyrus nokrek* Brunke, des. A. Brunke 2017 [red label] / AJB0000395 [identifier label].

**Paratypes** (3 ♀, cHay, NMW): same as holotype, AJB0000505; same as holotype except: 9–17.V.1996, E. Jendek and O. Sausa, AJB0000506, AJB0000507.

##### Diagnosis.

Within the Carnifex Group, *Bolitogyrus nokrek* can easily be distinguished by the darkened base of the elytra (Fig. 1G).

##### Description.

Measurements ♂ (n = 1): HW/HL 1.33; PW/PL 1.35; EW/ EL 1.19; ESut/PL 0.76; PW/HW 1.12; forebody length 7.1 mm.

Measurements ♀ (n = 3): HW/HL 1.30–1.38; PW/PL 1.20–1.31; EW/ EL 1.17–1.23; ESut/PL 0.74–0.77; PW/HW 1.12–1.13; forebody length 6.9–7.8 mm.

Extremely similar to *B. carnifex* and differing only in the following: apical antennomere only slightly paler than previous; pronotum almost entirely covered by dark marking; elytral darkened basally; abdominal segments III-IV almost entirely dark; dorsal face of tibia darkened; midfemur with distinct dark subapical marking; median lobe in lateral view with small, acute apex, slightly deflexed dorsal with flat ventral face instead of tooth, basal teeth present and appearing removed from ventral face, subapical teeth absent (Fig. 12E); median lobe in parameral view slightly constricted at midlength, apical portion strongly acuminate to acute and narrow apex bearing median carina (Fig. 12D); paramere in lateral view strongly projecting beyond and deflexed over apex of median lobe (Fig. 12G); paramere with distinctly narrower apical third, median group of peg setae extending basad of thin marginal group (Fig. 12F); female tergite VIII with narrow and deep emargination; female tergite X with dome-like expansion in apical half, without distinct carinae.

##### Distribution.

Figure 20C. Likely endemic to the Garo Hills, Meghalaya, India. Presently the westernmost species of the Carnifex Group.

##### Bionomics.

*Bolitogyrus nokrek* has been collected at 1400 m in April and May.

##### Etymology.

This species is named in recognition of the Nokrek UNESCO Biosphere Reserve in the Western Garo Hills of Meghalaya, India, where all known specimens were collected.

##### Comments.

Two females, (Khasi Hills Meghalaya (IRSNB)), and northern Kachin State, Myanmar (SEMC)) may belong to this or additional new species. They are excluded from the type series.

### Lasti Group

The members of the Lasti Group share the following character states: a deeply bilobed paramere, unique among Oriental *Bolitogyrus*; expansion of pronotal margin reduced; apex of median lobe deflexed dorsad. Thus far, the group is restricted to southern India.

#### Bolitogyrus lasti

Taxon classificationAnimaliaColeopteraStaphylinidae

Rougemont, 2001

Figs 1H , 12H–J , 20D (map)

Bolitogyrus lasti Rougemont, 2001: 111.

##### Type locality.

Cinchona [=Cinchona Rd. between Makkanduru and Hwy 88?], Karnataka, India.

##### Type material.

*Bolitogyrus lasti* Rougemont, 2001.

**Holotype** (♂, MMUE): Manchester Museum, HOLOTYPE [pink printed label] / F3008.11058 [printed] / HOLOTYPE Bolitogyrus lasti det. G. de Rougemont [red printed label] / AJB0000410 [identifier label].

**Paratype** (♀, MMUE): Manchester Museum, PARATYPE [printed label] / F3008.11058 [printed] / PARATYPE Bolitogyrus lasti det. G. de Rougemont [yellow printed label] / AJB0000521 [identifier label].

##### Other material.

**INDIA**: *Kerala*: 10 km WSW Munnar, Kallar Valley, 10.05 76.97, 1100–1200 m, 7–8.I.1999, D. Boukal, 1 ♂ 1♀, AJB0000411, AJB0000523 (NMW); same except, 15 km SW Munnar, Kallar Valley, 1000 m, 6-18.XII.1993, Boukal and Kejval, 1 ♀, AJB0000524 (NMW).

*Tamil Nadu*: Kadamparai [hydroelectric dam], V.1963, P.S. Nathan, 1 ♀, AJB0000522 (CNC).

##### Diagnosis.

This species can be distinguished by the following character states: head entirely dark; elytral disc dark with pale markings not extending onto epipleuron (Fig. 5F); pronotal margin narrow, almost without expansion (Fig. 4G).

##### Redescription.

Measurements ♂ (n = 3): HW/HL 1.34–1.42; PW/PL 1.26–1.29; EW/ EL 1.15–1.16; ESut/PL 0.83–0.85; PW/HW 0.98–1.0; forebody length 5.1–5.4 mm.

Measurements ♀ (n = 3): HW/HL 1.34–1.38; PW/PL 1.11–1.15; EW/ EL 1.12–1.17; ESut/PL 0.75–0.80; PW/HW 0.95–1.03; forebody length 5.3–5.8 mm.

Coloration: head, pronotum and abdomen entirely dark; elytra with pale yellow, raised markings, inner marking oval shaped and larger than outer circular marking; antennomeres 1-3 dark brown, 4-5 reddish, 6-10 dark brown, 11 distinctly paler, light brown to yellowish; palpi dark brown, apical segment paler; legs yellow, forecoxae dark brown, femur with dark band in apical half, tibia entirely dark with ventral face sometimes paler.

Head distinctly transverse; dorsal surface with moderately dense, clearly separated asetose punctures, frons with only scattered punctures and poorly impressed. Antennomere 6 slightly, 7-10 distinctly transverse and more or less symmetrical.

Pronotum distinctly (males) to slightly transverse, about as wide as head, with shallow micropunctures scattered on disc, becoming more distinct on anterior angles. Elytra slightly transverse, suture slightly to distinctly shorter than pronotum at middle.

Abdomen with disc of tergites III-VI distinctly and broadly impunctate; sternites III-IV with basal line distinctly projected posteriad at middle.

Median lobe in lateral view with apical portion projected ventrad, apex deflexed dorsad (Fig. 12I); median lobe in parameral view with basal teeth appearing at lateral margins (Fig. 12H); paramere shorter than median lobe, bilobed, lobes narrowly separated and slightly convergent (Fig. 12J); peg setae occurring in long group on each lobe from apex to just apicad of emargination (Fig. 12J); male sternite VIII with extremely shallow emargination and triangular glabrous area medially; male sternite IX moderately expanded at midlength, with distinct U-shaped emargination.

Female with tergite VIII entire (female paratype) to emarginate, emargination minute to small; tergite X elongate shield-shaped, with broadly rounded apex, disc slightly raised and without depressions or strong ridges.

##### Distribution.

Figure 20D. Endemic to the Western Ghats of India.

##### Bionomics.

*Bolitogyrus lasti* has been collected at 1000-1200 m during January, May, September and December.

##### Comments.

*Bolitogyrus lasti* is most similar externally to *B. ornatipennis* from Java but can be distinguished by the narrow pronotal expansion and more circular inner pale elytral spot.

#### Bolitogyrus tigris

Taxon classificationAnimaliaColeopteraStaphylinidae

Brunke sp. n.

http://zoobank.org/ADF07C91-BC0D-4638-91C2-0AF5AEFB0F7F

Figs 1I , 4G , 12K–M , 20D (map)

##### Type locality.

10 km SW Munnar, Idukki District, Kerala, India.

##### Type material.

**Holotype** (♂, NMW): S. INDIEN, Kerala, Cardamom hills, 1000m, 10km SW Munnar Vattiar [=Vattiyar?] (8) [printed] / 77°01'E 10°02'N, 5–17.12.1993, leg. Boukal & Kejval [printed] / AJB0000394 [identifier label] / HOLOTYPE *Bolitogyrus tigris* Brunke, des. A. Brunke 2017 [red label].

**Paratype** (♀, BMNH): India: *Tamil Nadu*: Coimbatore, Valparai, 1000 m, 15.X.1937, P.S. Nathan, AJB0000503.

##### Diagnosis.

*Bolitogyrus tigris* is easily recognized by a combination of the minutely expanded pronotal margin (Fig. 4G), orange head, the bicolored abdominal tergites IV-V, and orange and black marked elytra (Fig. 1I).

##### Description.

Measurements ♂ (n = 1): HW/HL 1.41; PW/PL 1.25; EW/ EL 1.17; ESut/PL 0.85; PW/HW 0.98; forebody length 6.0 mm.

##### Measurements

♀ (n = 1). HW/HL 1.28; PW/PL 1.22; EW/ EL 1.18; ESut/PL 0.83; PW/HW 1.0; forebody length 6.3 mm.

Coloration: body orange to reddish-orange (darkened paratype); head orange with central darkened area larger in female; disc of pronotum orange with central darkened area, a pair of dark spots laterally, a darkened areas along apex and base; elytra orange to reddish-orange (darkened paratype), with darkened area around scutellum; abdominal tergite III mostly dark with lateroapical areas orange, IV-V orange, dark basally and in narrow median stripe, VI entirely dark on disc, VII-VIII orange; antennomeres 1-5 brownish orange, 6-10 dark brown, 11 slightly paler than previous, lighter brown;

palpi brownish orange; legs brownish orange, with dorsal and lateral surfaces of mid and hind femur darkened, outer faces of tibia darker.

Head distinctly transverse; dorsal surface with moderately dense, clearly separated asetose punctures, frons with only scattered punctures. Antennomeres 8-10 transverse and asymmetrical.

Pronotum distinctly transverse, about as wide as head, convex and with shallow micropunctures scattered on disc, becoming more distinct on anterior angles. Elytra slightly transverse, suture slightly shorter than pronotum at middle; disc of elytron with slightly raised yellow v-shaped marking.

Abdomen with disc of tergites III-V distinctly impunctate; sternites III-IV with basal line distinctly projected posteriad at middle.

Median lobe in lateral view with apical portion projected ventrad, apex deflexed dorsad, with pair of basal teeth at level of apical fourth (Fig. 12L); median lobe in parameral view slightly dilated to apical fourth, spoon-shaped (Fig. 12K); paramere distinctly shorter than median lobe, distinctly bilobed, each lobe with pointed apex, constricted just basad of v-shaped median emargination (Fig. 12M); peg setae of each lobe arranged in marginal group and medial group, groups joined basally to form a ring (Fig. 12M); male sternite VIII with extremely shallow emargination and triangular glabrous area medially; male sternite IX moderately expanded at midlength, with distinct emargination.

Female with tergite VIII entire in single specimen studied, tergite X elongate triangular, with acute apex, raised disc of similar shape with broad median depression.

##### Distribution.

Figure 20D. Known only from the Cardamom Hills in the Western Ghats of India.

##### Bionomics.

*Bolitogyrus tigris* has been collected at 1000 m during October and December.

##### Etymology.

This taxon shares its specific epithet with the Bengal Tiger (*Panthera tigris tigris* (L.)) in recognition of its orange and black appearance, shared distribution, and India’s network of preserved forest habitats in the Anaimalai, Palni and Cardamom Hills regions of the Western Ghats. Habitat-focused conservation preserves populations of popular megafauna but also predaceous beetles like *Bolitogyrus tigris*, a ‘tiger’ in its own right

##### Comments.

*Bolitogyrus tigris* is easily recognized by coloration alone.

### Luteus Group

The members of the Luteus Group share the following character states: head and pronotum mostly orange to reddish-orange; disc of elytra with yellow, raised marking elongate and transverse (Fig. 4H); median lobe in lateral view with distinct apical portion that is expanded (Fig. 13C, 13F). Both members are lowland rainforest species and known only from the holotypes.

#### Bolitogyrus luteus

Taxon classificationAnimaliaColeopteraStaphylinidae

Brunke sp. n.

http://zoobank.org/91224AB7-6AC4-47AD-A256-E63DE73D3AFD

Figs 4H–I , 13A-D , 21A (map)

##### Type locality.

Banks of Khwae Noi River, near Sai Yok, Kanchanaburi, Thailand.

##### Type material.

**Holotype** (♂, cRou): THAILAND r. Kwae Noi, Ban Sai Yok, III.1987, G. de Rougemont [printed] / HOLOTYPE *Bolitogyrus luteus* Brunke, des. A. Brunke 2017 [red label] / AJB0000435 [identifier label].

##### Diagnosis.

This species is easily distinguished by the combination of the orange head and pronotum, transverse and raised yellow marking on the elytra (Fig. 4H-I) and the distinctly paler antennomere XI.

##### Description.

Measurements ♂ (n = 1): HW/HL 1.34; PW/PL 1.35; EW/ EL 1.23; ESut/PL 0.84; PW/HW 1.05; forebody length 4.7 mm.

Coloration: body orange, head with small medial darkened spot, pronotum with single median and pair of lateral dark spots, elytra each with large lateral and triangular marking that extends onto epipleuron only apically, elytra each with transverse and raised yellow marking (Fig. 4H); abdominal tergites VI with median dark spot, VII almost entirely dark with apex and base paler; antennomere 1 yellow, 2 -5 reddish, 6-10 dark brown, 11 paler, yellow; palpi yellow with apical segment darker; legs yellow with small ventroapical part of femur darker.

Head distinctly transverse; dorsal surface with moderately dense, clearly separated asetose punctures, frons with only scattered punctures. Antennomeres 6-7 slightly, 8-10 distinctly transverse, 10 slightly asymmetrical.

Pronotum distinctly transverse, about as wide as head, with very few shallow micropunctures scattered on disc, becoming more distinct on anterior angles. Elytra slightly transverse, suture slightly shorter than pronotum at middle; disc of elytron with slightly raised yellow transverse marking.

Abdomen with disc of tergites III-IV distinctly, V narrowly impunctate.

Median lobe in lateral view with apical portion sinuate due to expansion, projected ventrally, with large median tooth arising from median carina (Fig. 13A–B); median lobe in parameral view subparallel-sided, apical portion slightly acuminate, without basal teeth (Fig. 13C); paramere entire with median suture, distinctly longer than median lobe, with long and narrow apical portion, peg setae arranged in thin marginal row that extends basad to level of suture, thin median row extending basad of marginal row and slightly thicker at base (Fig. 13D); male sternite VIII with extremely shallow emargination and triangular glabrous area medially; male sternite IX moderately expanded at midlength, with distinct emargination.

Female unknown.

##### Distribution.

Figure 21A. Known only from the type locality in western Thailand.

##### Etymology.

The specific epithet refers to the warm, reddish-orange body coloration.

##### Bionomics.

The holotype was collected in March along the banks of a large river with several other species of *Bolitogyrus* and many other staphylinids (G. Rougemont, *pers. comm.*), which may have been flooded out from the forest with heavy rains.

##### Comments.

*Bolitogyrus luteus* can be recognized by coloration alone.

#### Bolitogyrus sepilok

Taxon classificationAnimaliaColeopteraStaphylinidae

Brunke sp. n.

http://zoobank.org/F9C45748-40AA-4A11-8559-74EE0E1B196F

Figs 2A , 5A , 13E–G , 19C (map)

##### Type locality.

Sepilok Nature Resort, Sabah, Borneo, Indonesia.

##### Type material.

**Holotype** (♂, cRou): SABAH, Sepilok N.R., in fungi on log, 13.II.2007, G. de Rougemont leg. [printed] / Bolitogyrus sp. Det. 2009, G. de Rougemont [printed] / HOLOTYPE *Bolitogyrus sepilok* Brunke, des. A. Brunke 2017 [red label] / AJB0000437 [identifier label].

##### Diagnosis.

This species is easily distinguished by the combination of the orange head and pronotum, transverse and raised yellow marking on the elytra (as in Figs 2A, 4H) and the abdominal tergites with deeply impressed and elongate punctures (Fig. 5A).

##### Description.

Measurements ♂ (n = 1): HW/HL 1.30; PW/PL 1.24; EW/ EL 1.24; ESut/PL 0.86; PW/HW 0.96; forebody length 4.3 mm.

Similar to *B. luteus* and differing only in the following: body with pale areas darker, dark reddish; abdominal tergites I-III with broad medial dark markings; palpi slightly darker; forecoxa with small dark marking at midlength; femur with dark subapical band, apex of hind tibia dark; antennomeres 1-3 orange with slightly darkened apices, 4-8 dark brown, 9 light brown, 10-11 light yellow; pronotum distinctly more elongate and strongly convex; pronotum relatively narrower than head; forebody length smaller; abdominal tergites III-VI with deeply impressed and elongate punctures in basal impressions; median lobe in lateral view with much larger expansion

in lateral view, with pair of broad basal teeth (Fig. 13F); median lobe in parameral view with basal teeth appearing medially, apical portion shorter and only slightly acuminate (Fig. 13E); paramere shorter than median lobe, narrower with smaller expansion, peg setae in broad single row reaching midlength, distant from midline (Fig. 13G).

##### Distribution.

Figure 19C. Known only from the type locality in northeastern Borneo but probably more widely distributed in remnant lowland forests.

##### Bionomics.

*Bolitogyrus sepilok* was collected from fungi on a log in February at a lowland elevation.

##### Etymology.

In recognition of the Kabili-Sepilok Forest Preserve, which adjoins the type locality and preserves a significant remnant of virgin lowland Dipterocarp forest in northern Borneo.

##### Comments.

*Bolitogyrus sepilok* can be recognized by its distinct habitus alone.

### Pictus Group

The members of the Pictus Group (*B. concavus*, *B. pictus*, *B. profundus*, *B. rougemonti*, *B. schillhammeri*) share a characteristic subbasal expansion of the paramere in lateral view (Fig. 15B, arrow). All species also possess a basal pair of teeth on the median lobe that are placed laterally (minute in *B. concavus*). The species are identical externally, though nearly all are allopatric. The three species that are known from multiple collecting events were found to exhibit color dimorphism, with bicolored and dark morphs. The presence of distinct bicolored and dark morphs is unique within Oriental *Bolitogyrus* but occurs in the Neotropical Buphthalmus Group (e.g., *B. salvini* (Sharp), *B. pulchrus* Brunke) (Brunke and Solodovnikov 2014).

#### Bolitogyrus pictus

Taxon classificationAnimaliaColeopteraStaphylinidae

Smetana & Zheng, 2000

Figs 5G , 14A–C , 21A (map)

Bolitogyrus pictus Smetana & Zheng, 2000a: 62.

##### Type locality.

Puwen, Xishuangbanna, Yunnan, China.

##### Type material.

*Bolitogyrus pictus* Smetana & Zheng, 2000.

**Holotype** (♀, ZIN): “Yun’an’ okr. Puzenya 900m 28-III-1957 D. Panphilov” [translation from Cyrillic as in original description] / “Yunnan near Puwen 900 1957-III-28 D. Bonfilofe” [translation from Chinese as in original description] / HOLOTYPE Bolitogyrus pictus A. Smetana. 1999 [red label] / AJB0000412 [identifier label].

The holotype of this species is a female and this initially caused doubt about the identity of *B. pictus* and the externally identical and sympatric *B. profundus*. Yuan et al. (2007) associated male characters with *B. pictus* based on a single male collected from near the type locality. However, it was unknown at the time that externally identical species of the Pictus Group exist in sympatry, which is rare in *Bolitogyrus*. To complicate matters further, Cai et al. (2015) described *B. profundus* based on a single male from the same locality as the single male identified as *B. pictus* by Yuan et al. (2007). With the dissection of females co-collected with both *B. pictus* and *B. profundus*, it became possible to recognize the two taxa based on the apex of female tergite X. Luckily, these taxa are not each other’s closest relatives, as the shape of female tergite X in *B. schillhammeri* is indistinguishable from *B. pictus*. Based on these associations, the male characters described by Yuan et al. (2007) happen to be correctly associated with *B. pictus* and do not refer to yet another species.

##### Other material.

**CHINA**: *Yunnan*: Xishuangbanna, ‘Menla’ [Mengla] county, 600-700 m, 9.VII.2003, Jiao-Yao Hu & Liang Tang, 1 ♂, AJB0000525 (SNUC).

**LAOS**: *Phongsali*: Phongsali env. [environs of], approx. 1500 m, 21.68 102.10, 6-17.V.2004, V. Kuban, 1 ♂, AJB0000416 (NHMB); Phongsali env. [environs of], 1300-1500 m, 1-15.V.2004, Lao Collector, 2 ♂, AJB0000527, AJB0000528 (cShi);

*Xiangkhouang*: Mt. Phou Sane, 30 km NE Phonsavan, 1300-1700 m, 19.630 103.334, 10-30.V.2009, M. Geiser, 1 ♂, AJB0000526 (NHMB); same except, 1420 m, Z. Kraus, 1 ♀, AJB0000582 (NHMB).

##### Diagnosis.

This species may be recognized by the following: head entirely dark; pronotum widest in posterior third; elytra partly dark, discal markings of elytra extending onto epipleuron, not forming v-shape (Fig. 2B, 5G), medial and lateral markings of different sizes and never entirely fused (Fig. 5G); apex of median lobe hooked in lateral view, broad and short in parameral view (Fig. 14B); paramere with short apical portion and at most slightly longer than median lobe (Fig. 14A).

##### Redescription.

Measurements ♂ (n = 5): HW/HL 1.40–1.47; PW/PL 1.30–1.36; EW/ EL 1.21–1.28; ESut/PL 0.77–0.84; PW/HW 1.01–1.06; forebody length 3.9–4.4 mm.

Measurements ♀ (n = 2): HW/HL 1.38–1.43; PW/PL 1.29–1.29; EW/EL 1.24–1.30; ESut/PL 0.79–0.81; PW/HW 1.00–1.03; forebody length 4.4–4.5 mm.

Coloration: color dimorphic, dark morph with dark pronotum and dark abdomen; bicolored morph with red pronotum and abdomen with tergites III-V entirely and base of VI reddish. Head entirely dark; elytra with raised median marking oval-shaped and larger than raised lateral marking, markings always connected, pale area extended onto epipleuron, epipleuron varying from pale at midlength to nearly entirely pale; scutellum dark; antennomere 1 yellow with darkened apex, 2-5 reddish, 6-10 dark brown, 11 either dark brown or vaguely paler; palpi yellow with apical segment slightly darker; legs yellow, fore and midfemur with small ventroapical dark marking, hind femur with small apical band.

Head distinctly transverse, dorsal surface with dense, clearly separated asetose punctures, frons with very few scattered punctures and deep, coarse Y-shaped impression. Antennomeres 6-7 quadrate to slightly transverse, 8-10 distinctly transverse and slightly asymmetrical, 10 longer than 9.

Pronotum distinctly transverse, about as wide as head, with a few shallow micropunctures on disc, becoming more distinct on anterior angles. Elytra slightly transverse, suture slightly shorter than pronotum at middle.

Abdomen with disc of tergites III-VI distinctly impunctate medially, some specimens with VI only narrowly impunctate; sternites III-IV with basal line distinctly projected posteriad at middle.

Median lobe in lateral view sinuate, apex pointed and sharply deflexed ventrad, basal teeth appearing at ventral face (Fig. 14B); median lobe in parameral view slightly dilated to wide and short apex, basal teeth appearing at lateral margins (Fig. 14A); paramere as long as or slightly longer than median lobe, entire but with median suture that extends to about widest point, with apical portion deflected slightly dorsad (Fig. 14A); peg setae arranged in marginal and medial groups, marginal group extending to basal third of median suture, medial group as loose row that extends basad of marginal group (Fig. 14C); male sternite VII with slight emargination and broad impunctate area medially; male sternite VIII with distinct emargination and triangular glabrous area medially; male sternite IX moderately expanded at midlength, with distinct emargination.

Female tergite VIII with median emargination deep and elongate; female tergite X shield-shaped, disc with distinct and entirely rounded dorsal expansion in apical half, apex truncate with narrow and rounded median projection.

##### Distribution.

Figure 21A. *Bolitogyrus pictus* is known from southern Yunnan, China, and northern Laos. It probably also occurs in northern Thailand.

##### Bionomics.

This species has been collected in March, May and July, at elevations ranging from 600-1500 m.

##### Comments.

*Bolitogyrus pictus* is most similar to allopatric *B. schillhammeri* (Myanmar) but differs by the broader apex of the paramere and hooked apex of the median lobe in lateral view.

#### Bolitogyrus schillhammeri

Taxon classificationAnimaliaColeopteraStaphylinidae

Brunke sp. n.

http://zoobank.org/056740B2-DB32-43E1-A6F2-84F94F4A8F5B

Figs 2B , 7A , 14D–F , 21B (map)

##### Type locality.

Mintaingbin Forest Camp, ca. 35 km north of Aungban, Shan State, Myanmar.

##### Type material.

**Holotype** (♂, NMW): MYANMAR: Shan State (MBS 146a), ca 35 km N Aungban, Mintaingbin Forest Camp, FIT, 20°55.20'N 96°33.60E, 11-23.6.2004, ca. 1320m [printed] / HOLOTYPE *Bolitogyrus schillhammeri* Brunke, des. A. Brunke 2017 [red label] / AJB0000413 [identifier label].

**Paratypes** (6 ♂, 3 ♀, NMW, cRou, IRSNB): same as holotype, AJB0000529, AJB0000530; same as holotype except, (MBS 81A), 31.V–8.VI.2002, AJB0000531, AJB0000532; same as holotype except, 81D, sifted, 31.V–8.VI.2002, H. Schillhammer & M. Hlaing, AJB0000533. Myanmar: *Kayin State*: ‘Carin Chebà’, 900–1100 m, V-XII.1888, L. Fea, 1 ♂ AJB0000535 (IRSNB), 1 ♀ AJB0000536 (IRSNB). *Shan State*: Kalaw, in fungi under decaying log, 19.III.1982, G. de Rougemont, 1 ♂, AJB0000534 (cRou); Taunggyi, decaying wood in waterfall pool, 6.VI.1980, G. de Rougemont, 1 ♀, AJB0000581 (cRou).

##### Diagnosis.

This species may be recognized by the following: head entirely dark; pronotum widest in posterior third; elytra partly dark, discal markings of elytra extending onto epipleuron, not forming v-shape, medial and lateral markings of different sizes and never entirely fused (Fig. 2B, 5G; apex of median lobe not hooked or flattened in lateral view, apical portion with flat ventral face in lateral view (Fig. 14E), broad and short in parameral view (Fig. 14D); paramere with long and elongate apical portion, slightly longer than median lobe (Fig. 14D).

##### Description.

Measurements ♂ (n = 5): HW/HL 1.38–1.45; PW/PL 1.26–1.33; EW/ EL 1.19–1.26; ESut/PL 0.77–0.82; PW/HW 0.99–1.04; forebody length 4.0–4.5 mm.

Measurements ♀ (n = 2): HW/HL 1.39–1.43; PW/PL 1.24–1.30; EW/ EL 1.22–1.24; ESut/PL 0.76–0.80; PW/HW 1.05–1.08; forebody length 4.6–4.9 mm.

Extremely similar to *B. pictus* and differing only in the following: median lobe in lateral view with apex not hooked, apical portion with ventral face straight (Fig. 14E); median lobe in parameral view with apical portion slightly narrower (Fig. 14D); apical portion of paramere distinctly more elongate and narrower (Fig. 14F).

##### Distribution.

Figure 21B. *Bolitogyrus schillhammeri* is known from three localities in the Shan Hills (incl. Karen hills) of Myanmar.

##### Bionomics.

This species has been collected in March, May and June at 900–1320 m. Specimens have been collected from rotten wood and from within fungi.

##### Etymology.

The species is named in honor of Harald (Harry) Schillhammer (NMW), the collector of many specimens of the type series, and a wonderful friend and field companion. Harry’s adventurous spirit and broad knowledge have led to many shared ‘eureka’ moments for staphylinid systematics.

##### Comments.

*Bolitogyrus schillhammeri* is most similar to *B. pictus* but can be distinguished by the narrower apex of the paramere and the non-hooked apex of the median lobe in lateral view.

#### Bolitogyrus rougemonti

Taxon classificationAnimaliaColeopteraStaphylinidae

Brunke sp. n.

http://zoobank.org/96E3DD61-B109-40C4-9150-4D7F4ED2ACEF

**Figure 6.**
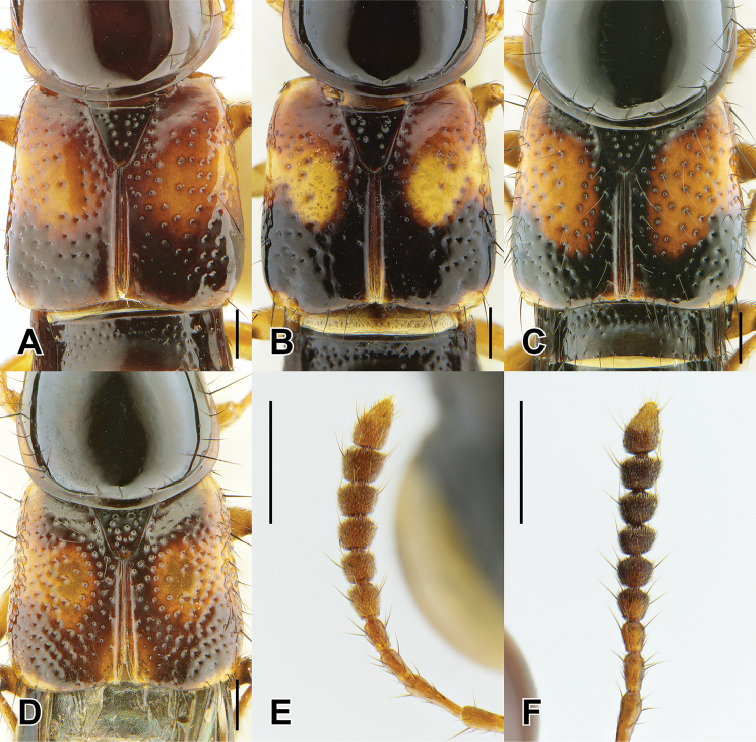
Elytra: *Bolitogyrus taiwanensis* (Hayashi) (**A**), *B. fukiensis* (Scheerpeltz) (**B**), *B. flavus* Yuan et al. (**C**) and *B. rougemonti* Brunke (**D**). Antenna: *B. taiwanensis* (**E**) and *B. flavus* (**F**). Scale bars: 0.5 mm.

Figs 6D , 14G–I , 21B (map)

##### Type locality.

Banks of Khwae Noi River, near Sai Yok, Kanchanaburi, Thailand.

##### Type material.

**Holotype** (♂, cRou): THAILAND r. Kwae Noi, Ban Sai Yok, III.1987, G. de Rougemont [printed] / HOLOTYPE *Bolitogyrus rougemonti* Brunke, des. A. Brunke 2017 [red label] / AJB0000414 [identifier label].

**Paratype** (♂, cRou): same data as holotype except: AJB0000415.

##### Diagnosis.

This species may be recognized by the following: head entirely dark; pronotum widest in posterior third; elytra partly dark, discal markings of elytra extending onto epipleuron, not forming v-shape, medial and lateral markings of different sizes and never entirely fused (Fig. 6C); apex of median lobe flattened in lateral view (Fig. 14H), broad and short in parameral view (Fig. 14G).

##### Description.

Measurements ♂ (n = 2): HW/HL 1.40–1.41; PW/PL 1.28–1.31; EW/ EL 1.23–1.29; ESut/PL 0.77–0.80; PW/HW 1.02–1.04; forebody length 4.3–4.5 mm.

Extremely similar to *B. pictus* and differing only in the following: only dark specimens known; head, pronotum and abdomen entirely dark; antennomere dark brown; median lobe in lateral view strongly flattened in apical portion, dilated subapically, basal teeth appearing removed from lateral margin (Fig. 14H); median lobe in parameral view with apical portion slightly narrower (Fig. 14G); paramere with shorter and distinctly acuminate apical portion (Fig. 14I), in lateral view strongly deflexed dorsad over apex of median lobe; female unknown.

##### Distribution.

Figure 21B. Known only from the type locality in western Thailand.

##### Bionomics.

The type series was collected in March along the banks of a large river, with many other beetles including *Bolitogyrus* (G. Rougemont, *pers. comm*.) that may have washed out from preferred deadwood microhabitats.

##### Etymology.

This species is named in honor of Mr. Guillaume de Rougemont (United Kingdom), the sole collector of this and many other Oriental *Bolitogyrus* species. Material from his personal collection has greatly increased the comprehensiveness of this monograph.

##### Comments.

At present, *B. rougemonti* is the only lowland member of the Pictus Group. This species is distinctive for its expansion of the median lobe in lateral view and shortened apex of the paramere.

#### Bolitogyrus profundus

Taxon classificationAnimaliaColeopteraStaphylinidae

Cai et al., 2015

Figs 14J–L , 21B (map)

Bolitogyrus profundus Cai et al., 2015: 469.

##### Type locality.

Menglun Nature Reserve, Xishuangbanna, Mengla County, Yunnan, China.

##### Type material.

The type specimen of this recently described, and well-illustrated species was not examined (Cai et al. 2015) but the illustrations in the description (Cai et al. 2015) and additional photos provided by the senior author of that paper were studied.

##### Other material.

**LAOS**: *Louangphabang*: Thong Khan [=Khang], 750 m, 19.578 101.966, 11-21.V.2002, J. Chalupek, 1 ♂, AJB0000538 (cShi); same except V. Kuban, 1 ♀, AJB0000586 (NHMB). *Phongsaly*: Phongsali env. [environs of], 1300-1500 m, 1-15.V.2004, Lao Collector, 1 ♂, AJB0000537 (cShi).

**THAILAND**: *Chiang Mai*: Doi Suthep, B. Degerbøl, 4.X.1958, 1 ♂, AJB0000422 (ZMUC); *Phetchabun*, Thung Salaeng Luang NP, Gang Wang Nam Yen, 16.610 100.890, 753 m, Malaise trap, T2083, 24-31.V.2007, Pongpitak Pranee & Sathit leg., 1 ♂, 1134 [identifier] (NHMO).

##### Diagnosis.

This species may be recognized by the following: head entirely dark; pronotum widest in posterior third; elytra partly dark, discal markings of elytra extending onto epipleuron, not forming v-shape, medial and lateral markings of different sizes and never entirely fused (Fig. 5G); apical portion of median lobe in lateral view not projecting ventrad or dorsad, not flattened, apex angulate and not hooked (Fig. 14K); apical portion in parameral view narrow and acuminate (Fig. 14J).

##### Redescription.

Measurements ♂ (n = 3): HW/HL 1.39–1.41; PW/PL 1.24–1.29; EW/ EL 1.25–1.26; ESut/PL 0.71–0.74; PW/HW 1.02–1.04; forebody length 4.1–4.3 mm.

Measurements ♀ (n = 1): HW/HL 1.39; PW/PL 1.24; EW/ EL 1.24; ESut/PL 0.73; PW/HW 1.02; forebody length 4.4 mm.

Extremely similar to *B. pictus* (including color dimorphism) and differing only in the following: median lobe in lateral view with apical portion more or less straight, apex angulate, basal teeth appearing removed from ventral face (Fig. 14K); median lobe in parameral view with apical portion distinctly longer and acuminate, basal teeth appearing laterally (Fig. 14J); paramere slightly shorter than median lobe, with apical portion more strongly acuminate, apex much narrower (Fig. 14L); female tergite X with apex not projecting at middle, apex widely acute and not truncate.

##### Distribution.

Figure 21B. *Bolitogyrus profundus* is known from southern Yunnan, China, northern Laos, and central and northern Thailand.

##### Bionomics.

*Bolitogyrus profundus* has been collected in May and October at elevations ranging from 750-1500 m.

##### Comments.

The acute apex of the median lobe was observed to be longer in specimens south of the type locality in Yunnan, China. This is attributed to intraspecific variation. *Bolitogyrus profundus* is similar to allopatric *B. concavus* (Meghalaya, India) but can be distinguished by the straight apex of the median lobe and the distinct rows of peg setae on the paramere.

#### Bolitogyrus concavus

Taxon classificationAnimaliaColeopteraStaphylinidae

Brunke sp. n.

Fig. 15A–C , 21C (map)

##### Type locality.

Nokrek National Park, West Garo Hills, Meghalaya, India.

##### Type material.

**Holotype** (♂, NMW): NE-INDIA, Meghalaya, W. Garo Hills, Nokrek NP., ca 1100 m [printed] / 25°29.6'N, 90°19.5E, 9.-17.5.1996, leg. Jendek & Sausa [printed] / Bolitogyrus vulneratus Fv., Det. A. Smetana, 2000 [written] / HOLOTYPE *Bolitogyrus concavus* Brunke, des. A. Brunke 2017 [red label] / AJB0000430 [identifier label].

##### Diagnosis.

This species may be recognized by the following: head entirely dark; pronotum widest in posterior third; elytra partly dark, discal markings of elytra extending onto epipleuron, not forming v-shape, medial and lateral markings of different sizes and never entirely fused (Fig. 5G); apical portion of median lobe in lateral view flattened and concave ventrad (Fig. 15B), in parameral view narrow and acuminate (Fig. 15A).

##### Description.

Measurements ♂ (n = 1): HW/HL 1.34; PW/PL 1.29; EW/ EL 1.20; ESut/PL 0.75; PW/HW 1.05; forebody length 4.4 mm.

Extremely similar to *B. pictus* and differing only in the following: only dark specimen known; median lobe in lateral view with apical portion thin, and concave ventrad, apex deflexed dorsad, basal teeth extremely weakly formed (Fig. 15B); median lobe in parameral view with apical portion distinctly longer and acuminate (Fig. 15A); paramere only slightly expanded about midway (Fig. 15A); peg setae arranged in marginal group and as several scattered setae medially (Fig. 15C). Female unknown.

##### Distribution.

Figure 21C. Known only from the type locality in the Garo Hills, Meghalaya, India.

##### Bionomics.

The holotype was collected at approximately 1100 m in May.

##### Etymology.

The species epithet refers to the apex of the median lobe, which is uniquely thin and concave in lateral view.

##### Comments.

*Bolitogyrus concavus* is most similar to allopatric *B. profundus* (China, Laos, Thailand) but is easily distinguished by the shape of the median lobe and the peg setae of the paramere, which are arranged mostly along the margin.

### Vulneratus Group

In species of the Vulneratus Group (*B. vulneratus*, *B. flavus*, *B. rufomaculatus*, *B. depressus*, *B. fukiensis*, *B. tumidus* and *B. taiwanensis*), the median lobe in lateral view is projected ventrad and hooked apically. Unlike species of the Loculus Group (Fig. 16F), the apex of the median lobe is not carinate in the Vulneratus Group (Fig. 13I, M). Most species (except lowland species *B. flavus* and *B. vulneratus*) also have a distinctive elongate projection at the middle of the apex of female tergite X.

#### Bolitogyrus vulneratus

Taxon classificationAnimaliaColeopteraStaphylinidae

(Fauvel, 1878)

Figs 13H–K , 22A (map)

Cyrtothorax vulneratus Fauvel, 1878: 165.
Bolitogyrus vulneratus : Smetana 1988, misidentification of B. himalayicus Brunke.

##### Type locality.

Cochinchina [=Southeast and Mekong River Delta regions], Vietnam.

##### Type material.

*Cyrtothorax vulneratus* Fauvel, 1878.

**Holotype** (♀, IRSNB): Cochinchine [written] / Tonkin museum [written] / vulneratus FvL. [written] / TYPE [pink, printed] / R. I. Sc. N. B., 17.479, Cyrtothorax, Coll. et det A. Fauvel [printed] / HOLOTYPE *Cyrtothorax vulneratus* Fauvel, 1878 Det. A. Brunke 2017 [red label] / AJB0000420 [identifier label].

##### Other material.

**VIETNAM**: *Quang Binh*: ‘mountains SW of Dong Hoi’ [translated from Cyrillic], 100m, 20-III-1963, Kabakov, AJB0000421, 1 ♂ (ZIN).

##### Diagnosis.

This species may be recognized by the following: head entirely dark; pronotum widest in posterior third; elytra partly dark, discal markings of elytra extending onto epipleuron, not forming v-shape, medial and lateral markings not entirely fused (Fig. 5G); apex of hind femur with only ventral half darkened (as in Fig. 2C); antennomere 7 distinctly transverse (Fig. 6F); median lobe in lateral view ventrally produced and hooked, without basal teeth (Fig. 13J).

##### Redescription.

Measurements ♂ (n = 1): HW/HL 1.50; PW/PL 1.23; EW/ EL 1.21; ESut/PL 0.74; PW/HW 1.05; forebody length 4.2 mm.

Measurements ♀ (n = 1): HW/HL 1.46; PW/PL 1.30; EW/EL 1.25; ESut/PL 0.78; PW/HW 1.08; forebody length 4.3 mm.

Coloration: head, pronotum and abdomen entirely dark; elytra with raised median marking oval-shaped and larger than raised lateral marking, markings connected, pale area extended onto epipleuron, epipleuron pale, pale area varying from near humerus to basal half; scutellum dark; antennomere 1 yellow with darkened apex, 2-5 reddish, 6-10 dark brown, 11 either dark brown or vaguely paler; palpi yellow with apical segment slightly darker; legs yellow, hind femur with small ventroapical dark marking.

Head strongly transverse, dorsal surface with dense, clearly separated asetose punctures, frons with few scattered punctures and deep, coarse Y-shaped impression. Antennomeres 6-10 distinctly transverse and 8-10 slightly asymmetrical.

Pronotum distinctly transverse, slightly wider than head, center of disc almost entirely without micropunctures, micropunctures becoming more distinct on anterior angles. Elytra slightly transverse, suture distinctly shorter than pronotum at middle.

Abdomen with disc of tergites III-VI distinctly impunctate medially, VI slightly more narrowly than others.

Median lobe in lateral view projected ventrad, with hooked apex, without basal teeth (Fig. 13J); median lobe in parameral view slender, with very slight expansion at about apical two-thirds, apical portion evenly converging to acute apex (Fig. 13I); paramere about as long as median lobe, slender, moderately expanded in apical two-thirds, apical portion broad and truncate (Fig. 13H), peg setae arranged in marginal group with some scattered setae mediad (Fig. 13K); male sternite VIII with distinct but shallow emargination and triangular glabrous area medially; male sternite IX moderately expanded at midlength, with distinct emargination.

Female tergite VIII with median emargination broad triangular, moderately deep; female tergite X elongate shield-shaped, with the disc evenly convex and lacking distinct raised areas, apex acuminate with apex projected.

##### Distribution.

Figure 22A. *Bolitogyrus vulneratus* is known only from low hills of central and southern Vietnam.

##### Bionomics.

One specimen was collected in March at 100 m.

##### Comments.

*Bolitogyrus vulneratus* has been confused with many small species in previous literature and in collections because, until now, only the female holotype was available. Study of the male genitalia reveals that this species is related to another lowland species, *B. flavus*, but can be distinguished by the presence of medial and lateral pale areas of the elytra. *Bolitogyrus vulneratus* is also similar externally to *B. hainanensis* but can be distinguished based on the simply hooked apex of the median lobe, absence of a clear median group of peg setae and a non-bilobed paramere. Two females (cShu) from southern Vietnam lowlands (60 km NE of Hoa Chi Min city) were studied that may represent an additional, undescribed species close to *B. vulneratus*. The elytral markings are longer and female tergite X has a distinct, raised area on the disc.

#### Bolitogyrus flavus

Taxon classificationAnimaliaColeopteraStaphylinidae

Yuan et al., 2007

**Figure 7.**
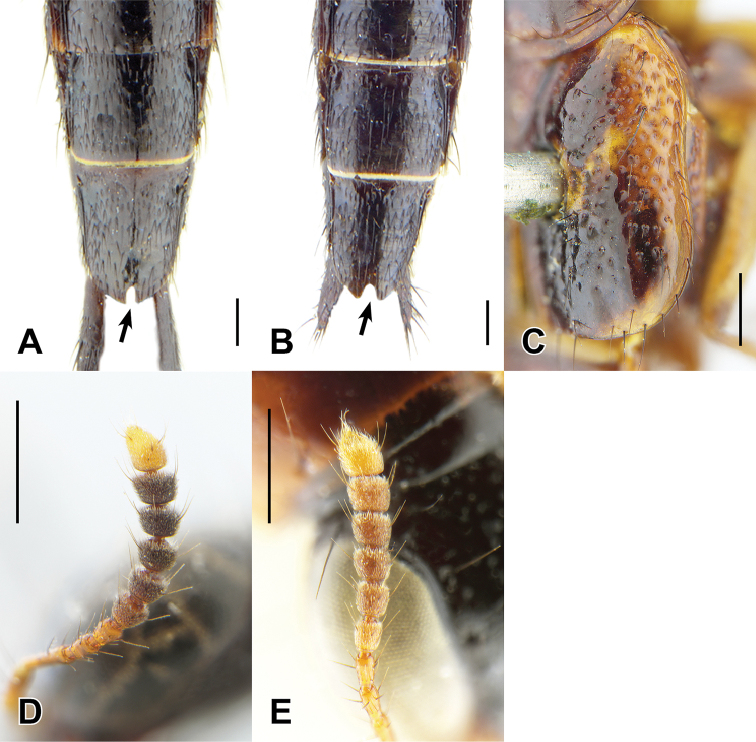
Female abdominal apex: *B. schillhammeri* Brunke (**A**) and *B. flavus* (**B**). Lateral view of elytron: *B. mulayitensis* Brunke (**C**). Antenna: *Bolitogyrus mulayitensis* Brunke (**D**) and *B. feai* Brunke (**E**). Scale bars: 0.5 mm.

Figs 2C , 6C , 6F , 7B , 13L–O , 22A

Bolitogyrus flavus Yuan et al., 2007: 148.

##### Type locality.

Manfei, Nabanhe Reserve, Xishuangbanna, Yunnan, China.

##### Type material.

*Bolitogyrus flavus* Yuan et al., 2007.

**Holotype** (♂, SNUC): Manfei, Nabanhe N.R., Jinghong City, Yunnan Prov., 9.I.2004, Li-Zhen Li & Liang Tang leg. [printed] / “[HOLOTYPE] Bolitogyrus flavus, Yuan, Zhao, Li & Hayashi, 2007, SHNU Collections” [red label] / AJB0000417 [identifier label].

##### Other material.

**CAMBODIA**: *Siem Reap*: Angkor, Preah Khan temple, Malaise trap, 2.V.2006, leg. Oul Yothin, 1 ♂, AJB0000589 (IRSNB). **LAOS**: *Bolikhamsai*: Lak Sao, Rd. no. 8, 500 m, 25–27.X.2003, S. Kurbatov, 1 ♂, AJB0000418 (NHMB); *Khammouane*: Ban Khounkham (Khun Kham) (Nahin), 18.217 104.514, 300 m, on tree fungi, disturbed primary rainforest, 3–5.VI.2008, A. Solodovnikov and J. Pedersen, 1 ♀, AJB0000554 (ZMUC); Ban Khoun Ngeun, 18.117 104.481, 200 m, 24–29.IV.2001, C.L. Pesa, 1 ♂, AJB0000555 (cShi), 1 ♀, AJB0000564 (NHMB); Nakai (environs of), route no. 8, 17.569 105.139, 560 m, 4–8.V.1995, E. Jendek & O. Sausa, 1 ♀, AJB0000565 (cShi). *Louangphabang*: Thong Khang, 750 m [should be 650 m], 19.585 101.966, 11–21.V.2002, J. Chalupek, 1 ♀, AJB0000566 (cShi).

**MYANMAR**: *Kayin State*: Kawkareik, V.1887, Fea, 1 ♂, AJB0000560 (IRSNB); *Shan State*: Shweudaung Wildlife Sanct., 23.085 96.225, ca. 360 m, FIT, 1-15.VIII.2002, M. Hlaing & A. Moe, 1 ♂, AJB0000563 (NMW).

**THAILAND**: *Chiang Mai*: Doi Suthep, 1000 m, on waterlogged wood in stream, 1979, G. de Rougemont, 1 ♂, AJB0000550 (cRou); Chiang Dao, 5-10.VII.1997, M. Klicha, 1 ♂, AJB0000558 (NMW); Chiang Dao, 26.X.2010, G. de Rougemont, 1 ♂, AJB0000562; Mae Rim, 3-4.VII.1997, M. Klicha, 1 ♂, AJB0000557 (NMW); *Kanchanaburi* Thong Pha Phum district, 30.VI-2.VII.1999, M. Klicha, 1 ♀, AJB0000556 (NMW); *Ranong*: Ranong, Doherty, 1 ♀, AJB0000552 (BMNH); *Yala*: Gunung Cang dun [=village], Betong, 25.III-22.IV.1993, Horak and Strnad, 1 ♂, AJB0000559 (NMW).

**VIETNAM**: *Hoa Bihn*: Hoa Binh, 1980, Clermont, 1 ♀, AJB0000553 (FMNH); *Nghe An*: Phuc Son, Fruhstorfer, N.-Dec., 1 ♀, AJB0000561 (FMNH); *Tuyen Quang*: Son Duong [translated from Cyrillic], 300 m, 23.III.1962, Kabakov, 1 ♀, AJB0000551 (ZIN).

##### Diagnosis.

This species may be recognized by the following: head entirely dark; pronotum widest in posterior third; elytra partly dark, base of elytra with broad pale area composed entirely of pale, raised yellow marking extending onto epipleuron (Fig. 2C, 6C); elytra with punctures along sutural area (Fig. 6C); paramere not constricted apically (Fig. 13L); female tergite VIII with wide triangular emargination (Fig. 7B).

##### Redescription.

Measurements ♂ (n = 5): HW/HL 1.31–1.38; PW/PL 1.31–1.39; EW/ EL 1.19–1.28; ESut/PL 0.75–0.81; PW/HW 1.04–1.08; forebody length 4.6–4.7 mm.

Measurements ♀ (n = 5): HW/HL 1.33–1.39; PW/PL 1.29–1.38; EW/ EL 1.18–1.25; ESut/PL 0.74–0.80; PW/HW 1.06–1.07; forebody length 4.2–4.9 mm.

Similar to *B. vulneratus* and differing only in the following: elytra with broad basal area composed entirely of raised yellow marking, pale area variable, as large spot extending to apical two-thirds (Fig. 6C) to extended to almost entire disc and leaving only apical angles dark (Fig. 2C); dark markings on femoral apices slightly larger on some specimens but never including dorsal face; head less transverse; antennomeres 7-10 distinctly transverse (Fig. 6F); pronotum more transverse; abdominal tergite VI not or only narrowly impunctate at middle; median lobe in lateral view with basal teeth formed as wide, toothed ridge (Fig. 13N); median lobe in parameral view with basal teeth barely visible, apical portion elongate and expanded, then suddenly constricted to acuminate and narrow apex (Fig. 13M); paramere elongate spoon-shaped (Fig. 13L), peg setae arranged in wide marginal group, convergent basally (Fig. 13O).

##### Distribution.

Figure 22A. *Bolitogyrus flavus* is widely distributed over the mainland of the southeast Oriental region.

##### Bionomics.

As far as known, *B. flavus* is a lowland to lower montane species and does not occur above 1000 m. Specimens have been collected in all months except February and September. *Bolitogyrus flavus* has been collected from fungi and rotten wood.

##### Comments.

*Bolitogyrus flavus* is the most commonly collected species of the genus in the Oriental region. Several single females could extend its range even further but may actually represent other related species.

#### Bolitogyrus rufomaculatus

Taxon classificationAnimaliaColeopteraStaphylinidae

(Shibata, 1979)

Figs 15D–E , 15G , 21D (map)

Cyrtothorax rufomaculatus Shibata, 1979: 26.
Bolitogyrus rufomaculatus : Smetana 1995.

##### Type locality.

Near Lushan, Nantou County, Taiwan.

##### Type material.

*Cyrtothorax rufomaculatus* Shibata, 1979.

**Paratypes** (♂, ♀, cShi): Taiwan: Kaohsiung Hsien [now Kaohsiung City County], near Luikuei [=Liugui], July 31^st^ 1976, Y. Shibata legt., 1 ♂ AJB0000432, 1 ♀ AJB0000433.

##### Other material.

**TAIWAN**: ‘Formosa’, S. Sauter, 1 ♀, AJB0000580 (ZMHB); *Chiayi*: Alishan National Scenic Area, road 18, to Youth Activity Centre, 2000 m, fern litter, 8.I.2009, S. Vit, 1 ♀, AJB0000549 (BMNH); Chashan, County road 129, km 33.5, 400 m, forest litter, 13.IV.2009, Viter, 1 ♂, AJB0000539 (cAss); *Kaohsiung City*: Hozan [=Fengshan], III.1910, S. Sauter, 1 ♂, AJB0000548 (ZMHB); *Nantou*: Huisun Forest Area, 24.075 120.998, 6.IV.2014, D. Huang (photo record); Lianhuachi, 16.VII.1973, Y. Kiyoyama, 1 ♀, AJB0000546 (cHay). Nanshanchi, 9.IV.1974, S. Takeda, 1 ♀, AJB0000541 (cHay); same except, 17.IV.1974, 2 ♀, AJB0000542 (cHay), AJB0000543 (SNUC); same except, 8.V.1978, K. Ando, 1 ♂, AJB0000544 (SNUC); same except, 3.V.1971, Y. Hayashi, 1 ♀, AJB0000545 (cHay); same except, 28.VII.1970, T. Kobayashi, 1 ♂, AJB0000547 (cHay); *New Taipei City*: Fuhosho [=Wucheng], Sauter, 1 ♀, AJB0000540 (NMW).

##### Diagnosis.

This species may be recognized by the following: head entirely dark; pronotum widest in posterior third; elytra partly dark, discal markings of elytra extending onto epipleuron, not forming v-shape, medial and lateral markings of different sizes and never entirely fused (Fig. 5G); median lobe in lateral view projected ventrad, with hooked apex and without distinct expansion, basal teeth appearing at or near ventral face (Fig. 15E); median lobe in parameral view with apex acuminate (Fig. 15D); paramere slightly to distinctly longer than median lobe and at most, as wide as median lobe in parameral view (Fig. 15D–E).

##### Redescription.

Measurements ♂ (n = 5): HW/HL 1.39–1.44; PW/PL 1.33–1.37; EW/ EL 1.19–1.26; ESut/PL 0.85–0.89; PW/HW 1.04–1.07; forebody length 4.3–4.7 mm.

Measurements ♀ (n = 5): HW/HL 1.34–1.44; PW/PL 1.35–1.45; EW/ EL 1.19–1.24; ESut/PL 0.82–0.89; PW/HW 1.04–1.09; forebody length 4.5–5.0 mm.

Similar to *B. vulneratus* and differing only in the following: females sometimes with small basal area of forecoxae dark; head slightly less transverse, frontal impression more weakly impressed; antennomeres less transverse, 7 slightly, 8-10 distinctly transverse, 10 asymmetrical and longer than 9; pronotum more transverse; suture longer relative to pronotum at middle; forebody slightly longer; pronotal disc slightly more micropunctate; median lobe in lateral view with basal teeth appearing at ventral face (Fig. 15E); median lobe in parameral view widened in apical fourth and then strongly narrowed to acuminate apex, basal teeth appearing mediad of lateral margin (Fig. 15D); paramere at widest point, no wider than median lobe, slightly to distinctly longer than median lobe, with longer, slightly to distinctly acuminate apical portion (Fig. 15D); peg setae arranged in clear medial and marginal groups, medial group extended basad of marginal group (Fig. 15G); male sternite VIII emargination very slight; female tergite VIII with narrower emargination, varying from moderately deep to deep U-shaped; female tergite X with apical projection raised and distinctly longer than wide.

##### Distribution.

Figure 21D. *Bolitogyrus rufomaculatus* is distributed broadly in Taiwan, in mountainous areas on the western half of the island.

##### Bionomics.

Specimens have been collected in January, March-May and July, at elevations ranging from 400-2000 m. Some specimens have been sifted from litter.

##### Comments.

*Bolitogyrus rufomaculatus* may be allopatric with *B. taiwanensis*, the only other species of the genus on Taiwan. These two species are easily distinguished using the color of the elytra and are not each other’s closest relatives. *Bolitogyrus rufomaculatus* is most similar to allopatric *B. depressus* (mainland China) but lacks the expansion of the median lobe in lateral view, has a basal tooth on the median lobe, and the paramere is longer than the median lobe.

#### Bolitogyrus depressus

Taxon classificationAnimaliaColeopteraStaphylinidae

Cai et al., 2015

Figs 15F , 21D (map)

Bolitogyrus depressus Cai et al., 2015: 454.

##### Type locality.

Nanling National Forest Park, Ruyuan County, Guangdong, China.

##### Type material.

The type series of this recently described, and well-illustrated species was not examined (Cai et al. 2015).

##### Diagnosis.

This species may be recognized by the following: head entirely dark; pronotum widest in posterior third; elytra partly dark, discal markings of elytra extending onto epipleuron, not forming v-shape, medial and lateral markings of different sizes and never entirely fused (Fig. 5G); median lobe in lateral view projected ventrad, with hooked apex, basal teeth absent (Fig. 15F); median lobe in parameral view with apex acuminate (Fig. 15D); paramere shorter than median lobe (Fig. 15F) and at widest point slightly wider than median lobe in parameral view.

##### Distribution.

Figure 21D. Known only from the type locality in Guangdong, China.

##### Bionomics.

The holotype was collected in July.

##### Comments.

Although Cai et al. 2015 did not directly compare *B. depressus* to *B. rufomaculatus*, they are extremely similar. Unlike *B. rufomaculatus*, *Bolitogyrus depressus* does not bear basal teeth on the median lobe, has a large expansion on the median lobe in lateral view, and the paramere is shorter than the median lobe. One female specimen from low elevation (600 m) from Guizhou, China was examined (cShi) that could be this species.

#### Bolitogyrus tumidus

Taxon classificationAnimaliaColeopteraStaphylinidae

Brunke sp. n.

http://zoobank.org/E9FE93D1-4A1B-4FC2-84FB-73AFE5085E81

Figs 15J–L , 21D (map)

##### Type locality.

Mt. Phu Phan, Hua Phan, Laos.

##### Type material.

**Holotype** (♂, NMW): NE-LAOS, prov. Hua Phan, Ban Saluei, Phou Pan, 1. -31.5.2011, 20°12'N 104°01E, 1500-1900 m, leg. Holzschuh [printed] / HOLOTYPE *Bolitogyrus tumidus* Brunke, des. A. Brunke 2017 [red label] / AJB0000423 [identifier label].

**Paratypes** (2 ♂, NHMB): Laos: *Hua Phan*: Mt. Phu Phan, ca. 1750 m, 20.205 104.010, 17.V.-3.VI.2007, Vit. Kuban, AJB0000672, AJB0000673.

##### Diagnosis.

This species may be recognized by the following: head entirely dark; pronotum widest in posterior third; elytra partly dark, discal markings of elytra extending onto epipleuron, not forming v-shape, medial and lateral markings of different sizes and never entirely fused (Fig. 5G); median lobe in lateral view projected ventrad, with hooked apex, basal teeth appearing distant from strongly expanded ventral face (Fig. 15K); median lobe in parameral view with apex acuminate (Fig. 15J).

##### Description.

Measurements ♂ (n = 3): HW/HL 1.34–1.39; PW/PL 1.21–1.27; EW/ EL 1.20–1.25; ESut/PL 0.73–0.79; PW/HW 1.08–1.08; forebody length 4.8–5.1 mm.

Similar to *B. vulneratus* and differing only in the following: elytral markings only weakly connected or separate in specimens available; head less transverse; antennomeres overall less transverse, antennomere 6 slightly, 7-10 distinctly transverse, 8-10 asymmetrical; forebody slightly longer; median lobe in lateral view with ventral face expanded subapically, apical portion projected ventrad, strongly constricted and hooked apically, basal teeth appearing distant from ventral face (Fig. 15K); median lobe in parameral view with apical portion expanded and subparallel, constricted to short, acuminate and narrow apex, basal teeth appearing mediad of lateral margins (Fig. 15J); paramere about as long as median lobe, narrower with longer apical portion (Fig. 15J); peg setae more numerous and densely arranged into discernable marginal and medial groups, marginal group broader (Fig. 15L); male sternite VIII emargination very slight. Female unknown.

##### Distribution.

Figure 21D. Known only from the type locality on Mt. Phu Pan in northern Laos.

##### Bionomics.

Specimens were collected at an elevational range of 1500-1900 m in May and June.

##### Etymology.

The specific epithet refers to the swollen, preapical portion of the median lobe.

##### Comments.

This species is most similar to *B. rufomaculatus* and *B. depressus* but can easily be distinguished by the distinctively expanded median lobe and its distribution in Laos.

#### Bolitogyrus taiwanensis

Taxon classificationAnimaliaColeopteraStaphylinidae

(Hayashi, 1991)

Figs 6A, E , 15G–I , 21D (map)

Cyrtothorax taiwanensis Hayashi, 1991: 45.
Bolitogyrus taiwanensis : Smetana 1995.

##### Type locality.

Taitung, Taitung County, Taiwan.

##### Type material.

*Cyrtothorax taiwanensis* Hayashi, 1991.

**Paratype** (♀, cHay): Formosa [=Taiwan], Taitung [Taitung County], Taitung city [from description], 19.VI.1972, Y. Kiyoyama, AJB0000434.

##### Other material.

Taiwan: *Pingtung*: Tahanshan Logging Road, 22.410 120.737, 24.X.2015, D. Huang (photo record); same except, 7.IV.2013, L. Wei-Ren (photo record, *in copula*).

##### Diagnosis.

This species may be recognized by the following: head entirely dark; pronotum widest in posterior third; elytra partly dark, with broadly pale basal area composed of yellow raised marking and slightly darker non-raised area (Fig. 6A); antennomere 7 only slightly transverse (Fig. 6E); female tergite X with disc flattened.

##### Redescription.

Measurements ♀ (n = 1): HW/HL 1.34; PW/PL 1.36; EW/ EL 1.22; ESut/PL 0.84; PW/HW 1.06; forebody length 5.0 mm.

Similar to *B. vulneratus* and differing only in the following: elytra with broad basal area composed of raised yellow marking and non-raised, slightly darker area; hind femur with dark apical marking larger, extending onto the very base of hind tibia; head less transverse; pronotum more transverse, with more numerous micropunctures; antennomere 6 quadrate, 7 slightly and 8-10 transverse; suture of elytra relatively longer than pronotum at middle; forebody slightly longer; median lobe and paramere in lateral view each with small expansion (Fig. 15H); [male characters based on illustrations in the literature] median lobe in parameral view widened in apical fourth and then strongly narrowed to acuminate apex (Fig. 15G); paramere at widest point, slightly wider than median lobe, slightly to distinctly longer than median lobe, with longer, distinctly acuminate apical portion (Fig. 15G); peg setae arranged in clear medial and marginal groups, medial group extended basad of marginal group (Fig. 15I); male sternite VIII emargination very slight; female tergite VIII with narrower emargination, moderately deep U-shaped; female tergite X with disc entirely flattened, apical projection distinctly longer than wide.

##### Distribution.

Figure 21D. Distributed in the southern part of Taiwan, on the eastern side of the major mountain system. More records are needed to discern how widespread this species is; currently it is not sympatric with *B. rufomaculatus*.

##### Bionomics.

This species has been observed in April-July and October at an approximate elevation of 1200 m. One pair was found *in copula* in October.

##### Comments.

The photo records of this species by D. Huang and L. Wei-Ren represent the only detailed data available as the type series is only reported from the general area of Taitung city. *Bolitogyrus taiwanensis* is most similar to allopatric *B. fukiensis* (eastern mainland China) but can be distinguished by the dark band on the hind femur and by the less transverse antennae. Females can be easily be distinguished by their flattened disc of tergite X (versus raised in *B. fukiensis*).

#### Bolitogyrus fukiensis

Taxon classificationAnimaliaColeopteraStaphylinidae

(Scheerpeltz, 1974)

Figs 2D , 6B , 21D (map)

Cyrtothorax fukiensis Scheerpeltz, 1974: 183.
Bolitogyrus fukiensis : Smetana and Zheng 2000a.

##### Type locality.

Kuatun [=Gua Dun], Fujian, China.

##### Type material.

*Cyrtothorax fukiensis* Scheerpeltz 1974.

**Holotype** (♀, NMW): ♂ [printed] / KUATUN, FUKIEN, China, 15.V.1946 (TSCHUNG SEN.) [printed] / Cyrtothorax fukiensis Scheerp. [written] / ex. coll. Scheerpeltz [printed] / HOLOTYPUS [red label] / TYPUS Cyrtothorax fukiensis, O. Scheerpeltz [red label] / fukiensis Scheerp. [orange label, written] / AJB0000419 [identifier label].

**Paratype** (♀, NMW): same data as holotype except, 15.IV.1946, AJB0000567.

##### Other material.

**CHINA**: *Fujian*: Gua Dun village, Wuyi Shan, 19.VI.1946, 1 ♀, AJB0000568 (NMW); Ziyungdongshan, NW-slopes, 900-1100 m, 25.766 117.333, 13-14.VII.2007, J. Turna, 3 ♀, AJB0000569, AJB0000570, AJB0000571 (NMW).

##### Diagnosis.

This species may be recognized by the following: head entirely dark; pronotum widest in posterior third; elytra partly dark, with broadly pale basal area composed of yellow raised marking and slightly darker non-raised area; antennomere 7 distinctly transverse; female tergite X with disc raised medioapically.

##### Redescription.

Measurements ♀ (n = 5): HW/HL 1.32–1.38; PW/PL 1.28–1.33; EW/ EL 1.15–1.19; ESut/PL 0.83–0.85; PW/HW 1.06–1.12; forebody length 4.9–5.2 mm.

Similar to *B. vulneratus* and differing only in the following: elytra with broad basal area composed of raised yellow marking and non-raised, slightly darker area (Fig. 6B); middle femur with small ventroapical area darkened; hind femur with dark apical marking slightly larger; head less transverse; elytral suture relatively longer than pronotum at middle; forebody slightly longer; male unknown; female with tergite VIII with emargination narrower, varying from moderately deep U-shape to minute triangular; female tergite X with apical projection distinctly longer than wide, disc distinctly raised at middle, top of raised area slightly depressed to flattened.

##### Distribution.

Figure 21D. Known only from a small area in Fujian, China.

##### Bionomics.

Specimens have been collected in April-July at elevations ranging from 900–1100 m.

##### Comments.

There have been concerns that this species was conspecific with *B. taiwanensis* due to the lack of a described male specimen and similarities in coloration (Smetana and Zheng 2000a). These species are both treated as valid herein based on clear differences in antennae and in the structure of female tergite X. This is presently the only described Oriental species with unknown male characters.

### Loculus Group

The diverse members of the Loculus Group (*Bolitogyrus feai*, *B. hainanensis*, *B. himalayicus*, *B. khasiensis*, *B. loculus*, *B. mulayitensis*, *B. nanus*, *B. pecki*, *B. smetanai*, and *B. solodovnikovi*) all share a projected carina at the apex of the median lobe, which appears as a tooth in lateral view and a thickened lip in parameral view. Nearly all species of this group also have a medial group of peg setae that are distinctly larger than those of the lateral group. Basal teeth of the median lobe, when present, are always placed laterally.

#### Bolitogyrus loculus

Taxon classificationAnimaliaColeopteraStaphylinidae

Cai et al., 2015

Fig. 16E–H , 22B (map)

Bolitogyrus loculus Cai et al., 2015: 460.

##### Type locality.

Menglun Nature Reserve, Xishuangbanna, Yunnan, China.

##### Type material.

The type series of this recently described, and well-illustrated species was not examined (Cai et al. 2015).

##### Diagnosis.

This species may be recognized by the following: head entirely dark; pronotum widest in posterior third; elytra partly dark, discal markings of elytra extending onto epipleuron, not forming v-shape, forming broadly pale basal area composed entirely of yellow raised marking (similar to Fig. 6C); paramere strongly constricted apically (Fig. 16E); female tergite VIII with narrow emargination (Fig. 7A).

##### Distribution.

Figure 22B. Known only from the type locality in southern Yunnan, China.

##### Bionomics.

The type series was collected in February at elevations ranging from 560-860 m.

##### Comments.

*Bolitogyrus loculus* is externally similar to *B. flavus*, especially to morphs of the latter with the minimum extent of pale coloration on the elytra (Fig. 6C). However, *B. loculus* can be recognized by the sparsely punctate sutural area of the elytra and the entirely darkened apex of the hind femur.

#### Bolitogyrus hainanensis

Taxon classificationAnimaliaColeopteraStaphylinidae

Cai et al., 2015

Figs 16A–D , 22B (map)

Bolitogyrus hainanensis Cai et al., 2015: 457.

##### Type locality.

Mt. Yingge Ling [base of], Hainan, China.

##### Type material.

The type series of this recently described, and well-illustrated species was not examined (Cai et al. 2015).

##### Diagnosis.

This species may be recognized by the following: head entirely dark; pronotum widest in posterior third; elytra partly dark, discal markings of elytra extending onto epipleuron, not forming v-shape, medial and lateral markings of different sizes and never entirely fused (Fig. 5G); hind femur with dark marking on ventral portion only (as in Fig. 2C; antennomere 7 distinctly transverse (Fig. 6F).

##### Distribution.

Figure 22B. This species may be endemic to Hainan island, China.

##### Bionomics.

Specimens have been collected in November and December at elevations ranging from 450-666 m.

##### Comments.

Except for one, much larger, member of the Carnifex Group, *B. hainanensis* is presently the only species of *Bolitogyrus* on Hainan island, China. *Bolitogyrus hainanensis* is externally similar to *B. vulneratus* and *B. pecki* but differs by the bilobed paramere, presence of a medial group of peg setae and the recurved apex of the median lobe in lateral view.

#### Bolitogyrus solodovnikovi

Taxon classificationAnimaliaColeopteraStaphylinidae

Brunke sp. n.

http://zoobank.org/4ABE6545-BE53-459F-B606-387F6F422AD1

Figs 2E , 5H , 16I–K , 21A (map)

##### Type locality.

Ban Thongvay, Muang Paxon, Bolaven Plateau, Champasak, Laos.

##### Type material.

**Holotype** (♂, ZMUC): LAOS: Champasak prov.: Bolaven Plt., Muang Paxong, Ban Thongvay, 8–16.vi.2008, N15°14.054, E106°31.867, 1200 m [printed] / A. Solodovnikov & J. Pedersen leg. Edge of disturbed primary rainforest near clearing; flight intercept trap. ZMUC collection. [printed] / HOLOTYPE *Bolitogyrus solodovnikovi* Brunke, des. A. Brunke 2017 [red label] / AJB0000427 [identifier label].

**Paratypes** (3 ♀, NMW, ZMUC): Laos: *Champasak*: Bolaven Plateau, between Paksong and Ban Nam Thang, 26-27.V.1996, H. Schillhammer, 1 ♀, AJB0000575; Bolaven Plataeu, Muang Paxong, Ban Thongvay, 1200 m, 15.234 106.531, edge of disturbed primary rainforest near clearing, fight intercept trap, 8–16.VI.2008, A. Solodovnikov and J. Pedersen, AJB0000576; Bolaven Plateau, Muang Paxong, Ban Hoyayteuy, Mt. Phu Din, 1100 m, 15.054, 106.289, disturbed primary rainforest, on tree fungi, 13–14.VI.2008, A. Solodovnikov and J. Pedersen, AJB0000577 1 ♀, ZMUC00046172 1 ♂.

##### Diagnosis.

This species may be recognized by the following: head entirely dark; pronotum widest in posterior third; elytra partly dark, discal markings of elytra extending onto epipleuron, forming v-shape (Fig. 2E, 5H); epipleuron not entirely pale (Fig. 5H); frons with only vague traces of microsculpture.

##### Description.

Measurements ♂ (n = 1): HW/HL 1.35; PW/PL 1.32; EW/ EL 1.24; ESut/PL 0.79; PW/HW 1.05; forebody length 4.6 mm.

Measurements ♀ (n = 3): HW/HL 1.31–1.40; PW/PL 1.30–1.34; EW/ EL 1.18–1.23; ESut/PL 0.80–0.83; PW/HW 1.05–1.08; forebody length 4.8–5.0 mm.

Coloration: head entirely dark; pronotum entirely reddish; scutellum reddish, at least at base, disc of elytra dark with yellow v-shaped, raised marking, base sometimes broadly reddish; epipleuron not entirely pale, with subapical darkened area; abdominal tergites III-V entirely reddish, VI varying from dark with reddish base to entirely reddish; antennomere 1 yellow with darkened apex, 2-5 light brownish-red with darkened apex, 6-10 dark brown, apical segment brownish to dark brown; palpi yellow-brown with apical segment slightly darker; legs yellow, midfemur with ventroapical darkened area, hind femur with apical area of femur darkened.

Head distinctly transverse, dorsal surface with moderately dense, clearly separated asetose punctures, frons with few scattered punctures and distinct Y-shaped impression. Antennomeres 6 quadrate, 7 slightly and 8-10 distinctly transverse, 10 more elongate than 9, 8-10 slightly asymmetrical.

Pronotum slightly transverse to quadrate, about as wide as head, with sparse moderately impressed micropunctation on disc, becoming more distinct on anterior angles. Elytra distinctly transverse, suture distinctly shorter than pronotum at middle.

Abdomen with disc of tergites III-V distinctly, VI narrowly impunctate medially; sternites III-IV with basal line distinctly projected posteriad at middle.

Median lobe in lateral view with narrowed apical portion nearly straight, apex with tooth (Fig. 16J); median lobe in parameral view with expansion in apical two-thirds, with wide basal tooth set on ridge, apical portion evenly converging to acute apex (Fig. 16I); paramere slightly longer than median lobe, elongate spoon-shaped, apex broadly rounded (Fig. 16K); peg setae arranged in thin marginal group, with a fragment of a median group consisting of a few setae basad of marginal group (Fig. 16K); male sternite VIII with distinct but shallow emargination and triangular glabrous area medially; male sternite IX moderately expanded at midlength, with distinct, deep emargination.

Female tergite VIII with median emargination moderately deep and varying from triangular to U-shaped; female tergite X elongate shield-shaped, with moderately projecting and rounded apex, disc distinctly raised, this area longitudinally impressed at middle.

##### Distribution.

Figure 21A. Known only from the Bolaven Plateau in southern Laos.

##### Bionomics.

The type series was collected during May-June at 800-1200 m elevation. Specimens were collected using flight intercept traps and from tree fungi.

##### Etymology.

This species is named in honor of Alexey Solodovnikov (ZMUC). His positive energy and endless support as a PhD supervisor and now, as close colleague and friend, continue to develop the next generation of systematists and push staphylinid research forward.

##### Comments.

*Bolitogyrus solodovnikovi* is externally similar to allopatric *B. feai* and *B. mulayitensis* (both Myanmar) but can be distinguished by the epipleuron, which is only partly pale.

#### Bolitogyrus feai

Taxon classificationAnimaliaColeopteraStaphylinidae

Brunke sp. n.

http://zoobank.org/88CEA0A4-F74B-49D4-A7C0-64843E93529A

Figs 7E , 16L–O , 21A (map)

##### Type locality.

Karen Hills, Kayin State, Myanmar.

##### Type material.

**Holotype** (♂, IRSNB): Carin Cheba, 900–1100m, L. Fea, V XII-88 [printed] / Coll. et det. A. Fauvel, Cyrtothorax vulneratus, R.I.Sc.N.B. 17.479 [printed] / HOLOTYPE *Bolitogyrus feai* Brunke, des. A. Brunke 2017 [red label] / AJB0000426 [identifier label].

##### Diagnosis.

This species may be recognized by the following: head entirely dark; pronotum widest in posterior third; elytra partly dark, discal markings of elytra extending onto epipleuron, forming v-shape (as in Fig. 2E); epipleuron entirely pale (as in Fig. 7C); antennomere 8-9 weakly transverse (Fig. 7E); frons with only vague traces of microsculpture.

##### Description.

Measurements ♂ (n = 1): HW/HL 1.42; PW/PL 1.33; EW/ EL 1.22; ESut/PL 0.86; PW/HW 0.94; forebody length 4.6 mm.

Similar to *B. solodovnikovi* and differing only in the following: epipleuron entirely pale (Fig. 7C); pronotum with broad medial darkening; apical antennomere slightly paler (Fig. 7E); elytral suture relatively longer than pronotum at midline; pronotum slightly narrower than head; abdominal tergite VI entirely dark and punctate medially; antennomeres without distinctly darkened apices; median lobe in lateral view with apical portion more strongly narrowed to apex and slightly sinuate, not distinctly hooked, without basal teeth (Fig. 16M); median lobe in parameral view with shorter, acuminate apical portion (Fig. 16L); paramere about as long as median lobe, less elongate, peg setae arranged in shorter marginal group, median group composed of larger peg setae placed in a broader row basad of marginal group (Fig. 16O); male sternite VIII with shallower emargination. Female unknown.

##### Distribution.

Figure 21A. Known from the Karen Kills (Kayin State) in Myanmar.

##### Bionomics.

The holotype was collected in May at 900–1100 m.

##### Etymology.

This species is named after Leonardo Fea (1852–1903), an Italian explorer and naturalist who collected a rich variety of *Bolitogyrus* specimens from Myanmar.

##### Comments.

*Bolitogyrus feai* is most similar to *B. mulayitensis* from southern Myanmar but can be distinguished by the longer apical antennomeres and the presence of only traces of microsculpture on the frons.

#### Bolitogyrus mulayitensis

Taxon classificationAnimaliaColeopteraStaphylinidae

Brunke sp. n.

http://zoobank.org/8DC5E4D7-0F44-42B0-8AD6-BF171F54FA3C

Fig. 7C, D , 17A–C , 21A (map)

##### Type locality.

Mt. Mulayit, Kayin State, Myanmar.

##### Type material.

**Holotype** (♂, SDEI): Tenasserim, M. Mooleyit [=Mt. Mulayit], 1800–1900m, Fea, Marzo, 1887 [printed] / Cyrtothorax vulneratus Fvl. [written] / Coll. Kraatz [printed] / HOLOTYPE *Bolitogyrus mulayitensis* Brunke, des. A. Brunke 2017 [red label] / AJB0000424 [identifier label].

**Paratype** (1 ♀, IRSNB): same data as holotype with AJB0000425.

##### Diagnosis.

This species may be recognized by the following: head entirely dark; pronotum widest in posterior third; elytra partly dark, discal markings of elytra extending onto epipleuron, forming v-shape (as Figs 2E, 7C); epipleuron entirely pale (Fig. 7C); antennomere 8-9 distinctly transverse (Fig. 7D); frons with distinct microsculpture.

##### Description.

Measurements ♂ (n = 1): HW/HL 1.38; PW/PL 1.42; EW/ EL 1.22; ESut/PL 0.82; PW/HW 1.07; forebody length 4.5 mm.

Measurements ♀ (n = 1): HW/HL 1.37; PW/PL 1.36; EW/ EL 1.20; ESut/PL 0.90; PW/HW 1.03; forebody length 4.5 mm.

Similar to *B. solodovnikovi* and differing only in the following: pronotum with broad medial darkening; epipleuron entirely pale (Fig. 7C); abdominal tergites IV-V with medial darkening, VI entirely dark; apical antennomere distinctly paler than previous, yellow; head with distinct microsculpture on frons; pronotum distinctly (male) to slightly (female) more transverse; elytral suture relatively longer than pronotum at middle in female; abdominal tergite VI not distinctly impunctate at middle; median lobe in lateral view with apical portion triangular and ventral face slightly inflated, apex with minute tooth formed from median carina, basal teeth present and appearing removed from ventral face (Fig. 17B); median lobe in parameral view only weakly expanded at apical two-thirds, with apical portion shorter, with apex acute and rounded, basal teeth appearing at lateral margins (Fig. 17A); paramere far narrower, with long apical portion narrowed to apex, medial group of peg setae present and extended apicad to overlap with marginal group, medial setae distinctly larger than marginal setae (Fig. 17C); female tergite VIII with minute triangular emargination; female tergite X shield-shaped, with broadly rounded but projected apex, disc slightly raised and with slight longitudinal impression.

##### Distribution.

Figure 21A. Known only from Mt. Mulayit, in southern Myanmar.

##### Bionomics.

The type series was collected in March at a relatively high elevation (1800–1900 m).

##### Etymology.

The species is named after Mulayit Taung, a mountain in southern Myanmar. The type series was collected near its summit.

##### Comments.

*Bolitogyrus mulayitensis* is most similar to *B. feai* but can be distinguished by the distinctly transverse apical antennomeres and strong microsculpture on the frons.

#### Bolitogyrus smetanai

Taxon classificationAnimaliaColeopteraStaphylinidae

Brunke sp. n.

http://zoobank.org/471D1B88-D001-4701-B1D6-E629AE3C3C4B

Fig. 2F , 4F , 17D–F , 22B (map)

##### Type locality.

Banks of Khwae Noi River, near Sai Yok, Kanchanaburi, Thailand.

##### Type material.

**Holotype** (♂, cRou): THAILAND r. Kwae Noi, Ban Sai Yok, III.1987, G. de Rougemont [printed] / HOLOTYPE *Bolitogyrus smetanai* Brunke, des. A. Brunke 2017 [red label] / AJB0000436 [identifier label].

**Paratype** (♂, IRSNB): Myanmar: *Kayin State*: Carin Cheba, 900–1100m, L. Fea, V XII-88, AJB0000574.

##### Diagnosis.

This species can be recognized by the following combination of characters: orange frons contrasting with dark head (Fig. 2F); pronotal margin only weakly expanded, no more than three lateral puncture widths wide; apical antennomere not distinctly paler than previous segments (Fig. 2F).

##### Description.

Measurements ♂ (n = 2): HW/HL 1.31–1.34; PW/PL 1.28–1.30; EW/ EL 1.18–1.22; ESut/PL 0.81–0.81; PW/HW 1.02–1.03; forebody length 4.0–4.1 mm.

Coloration: head dark with orange frons; pronotum reddish orange with hourglass-shaped dark marking medially; elytral disc dark brown to dark brownish-red, with orange to yellow marking at middle and along suture, apical angles and humeri paler; scutellum dark; epipleuron pale along its length; abdominal tergites III-VI ranging from almost entirely dark, to reddish with central dark marking, VII entirely dark; antennomere 1 yellow, 2-5 reddish with dark apices, 6-10 dark brown, 11 vaguely paler; palpi yellowish; legs yellowish with small dark marking at ventral apex of femora.

Head distinctly transverse, dorsal surface with moderately dense but clearly separated, asetose punctures, frons with few scattered punctures. Antennomeres 8-10 slightly transverse and asymmetrical.

Pronotum distinctly transverse, center of disc with very few micropunctures, about as wide as head. Elytra moderately transverse, suture shorter than pronotum at middle; elytral disc bearing a pair of small, raised, oval and light yellow markings near the center, and an additional, smaller pair laterad.

Abdomen with disc of tergites III-V distinctly, impunctate at middle.

Median lobe in lateral view evenly narrowed to apex, ventral face flat, with minute pair of median teeth formed from median carina (Fig. 17E); median lobe in parameral view subparallel to rounded, acuminate apical portion, without subapical or basal teeth (Fig. 17D); paramere longer than median lobe, elongate spoon-shaped, peg setae in thin marginal group, basal-most setae removed from margin and disconnected from main group (Fig. 17F); male sternite VII with distinct, shallow emargination, and with triangular glabrous area medially; male sternite VIII with very slight emargination and elongate triangular, flattened and glabrous area medially; male sternite IX distinctly expanded at midlength, with distinct emargination.

Female unknown.

##### Distribution.

Figure 22B. Known from western Thailand and the Karen Hills of Myanmar.

##### Bionomics.

Specimens were collected in March and May at both lowland and montane elevations (900–1100 m). It is unlikely that *B. smetanai* occurs over such a broad range of elevations and it is possibly that either Fea’s material was mislabeled or the specimen from Thailand had washed down with heavy rain from a much higher point.

##### Etymology.

This charming species is named in honor of Aleš Smetana (CNC), a close Canadian colleague and wealth of knowledge concerning the Staphylininae. His taxonomic contributions on the subfamily, including several on *Bolitogyrus*, form the modern treatments of the Nearctic and Oriental faunas.

##### Comments.

*Bolitogyrus smetanai* is easily recognized by its small size, dark apical antennomeres and orange frons.

#### Bolitogyrus khasiensis

Taxon classificationAnimaliaColeopteraStaphylinidae

Brunke sp. n.

http://zoobank.org/71E089D3-885C-460D-9F06-4CC3E9D877C2

Fig. 17G-H , 21C (map)

##### Type locality.

Mawsynram, Meghalaya, Khasi Hills, India.

##### Type material.

**Holotype** (♂, BMNH): NE INDIA, MEGHALAYA, KHASI Hills, MAWSYNRAM, 25°18'N 91°29'E, 800±100m, L. Dembický leg., 5–9.vi.2006, BMNH 2006-48 [printed] / Bolitogyrus, A. Solodovnikov det. 2007 [printed] / HOLOTYPE *Bolitogyrus khasiensis* Brunke, des. A. Brunke 2017 [red label] / AJB0000428 [identifier label].

**Paratype** (♀, BMNH): same as holotype but with AJB0000429.

##### Diagnosis.

This species can be distinguished by the following character states: head entirely dark; elytral disc dark with pale markings not extending halfway to epipleural margin (Fig. 5F), medial marking oval-shaped; pronotal margin at its widest point no more than three lateral puncture widths wide but still distinctly expanded at hind angles; hind tibia entirely pale; disc of head with moderately impressed punctures and distinct frontal impression.

##### Description.

Measurements ♂ (n = 1): HW/HL 1.34; PW/PL 1.26; EW/ EL 1.19; ESut/PL 0.79; PW/HW 1.0; forebody length 4.3 mm.

Measurements ♀ (n = 1): HW/HL 1.35; PW/PL 1.27; EW/ EL 1.19; ESut/PL 0.76; PW/HW 1.09; forebody length 4.3 mm.

Coloration: head, pronotum and abdomen entirely dark; elytra with yellow, raised markings, medial marking thinly connected to smaller lateral marking that does not extend halfway to epipleural margin; antennomere 1 yellow with darkened apex, 2-5 reddish with darkened apices, 6-11 dark brown; palpi yellow with apical segment slightly darkened; legs yellow, mid and hind tibia with darkened ventroapical area.

Head distinctly transverse, dorsal surface with moderately dense, clearly separated asetose punctures, frons with only scattered punctures and distinctly impressed. Antennomere 6 slightly, 7-10 distinctly transverse and 8-10 asymmetrical.

Pronotum distinctly transverse, about as wide as head to slightly wider, with a few shallow micropunctures on disc, becoming more distinct on anterior angles. Elytra slightly transverse, suture shorter than pronotum at middle.

Abdomen with disc of tergites III-VI distinctly impunctate medially.

Median lobe in lateral view strongly constricted in apical portion, with apical tooth formed from median carina, basal teeth absent, ventral face more or less straight (similar to Fig. 17E); median lobe in parameral view subparallel before short, vaguely acuminate and acute apex bearing double-toothed carina or ‘lip’ (Fig. 17H); paramere distinctly longer than median lobe, elongate spoon-shaped, evenly converging to apex (Fig. 17G), in lateral view with apex abruptly deflexed dorsad; peg setae arranged in thin marginal group that is convergent basally, apex without dense field (Fig. 17I); apex of male sternite VIII with slight emargination and triangular glabrous area medially; male sternite IX moderately expanded at midlength, with distinct emargination.

Female with tergite VIII entire; tergite X elongate shield-shaped, with broadly rounded but projected apex, disc distinctly raised and without depressions or strong ridges.

##### Distribution.

Figure 21C. Known only from and probably endemic to the Khasi Hills of Meghalaya, India.

##### Bionomics.

The type series was collected in June at approximately 800 m. The area around the type locality is also considered to be the wettest terrestrial place on earth, receiving record annual rainfall levels.

##### Etymology.

The species epithet refers to the type locality in the Khasi Hills of Meghalaya, India, and recognizes this global biodiversity hotspot.

##### Comments.

*Bolitogyrus khasiensis* is most similar externally to northeastern Indian species *B. himalayicus*, *B. nanus* and *B. concavus* but can be distinguished by lateral discal elytral marking, which does not extend halfway to the epipleural margin. It shares this feature with *B. lasti* and *B. ornatipennis* but has an oval-shaped medial discal elytral marking and distinct expansion of the pronotal margin.

#### Bolitogyrus himalayicus

Taxon classificationAnimaliaColeopteraStaphylinidae

Brunke sp. n.

http://zoobank.org/0F325FEC-2A81-40DC-A2E3-4AD2468501ED

Figs 17I–J , 20C (map)

##### Type locality.

Sevoke, Darjeerling, West Bengal, India.

##### Type material.

**Holotype** (♂, MHNG): India, W. Bengal, Darjeerling dist, Sevoke, 200 m, 7.X.78, Besuchet Löbl / Cyrtothorax vulneratus Smetana det. 1986 [written] / AJB0000578 [identifier label] / HOLOTYPE *Bolitogyrus himalayicus* Brunke, des. A. Brunke 2017 [red label].

##### Diagnosis.

This species can be distinguished by the following character states: head entirely dark; elytral disc dark with pale markings extending halfway to epipleural margin (Fig. 5G); pronotal margin at its widest point no more than three lateral puncture widths wide but still distinctly expanded at hind angles; forebody less than four millimeters; apex of median lobe with single-toothed carina or ‘lip’ (Fig. 17J); paramere with dense apical cluster of peg setae (Fig. 17I).

##### Description.

Measurements ♂ (n = 1): HW/HL 1.36; PW/PL 1.34; EW/ EL 1.20; ESut/PL 0.83; PW/HW 1.07; forebody length 3.7 mm.

Extremely similar to *B. khasiensis* and differing only in the following: medial and lateral elytral markings more broadly connected, pale area of epipleuron not restricted to humeral spot, pale in entire basal half; pronotum slightly more transverse; elytral suture relatively longer than pronotum at middle; forebody distinctly shorter and thinner; median lobe in parameral view with apex distinctly acuminate and acute, apical portion with single-toothed carina or ‘lip’ (Fig. 17J); paramere with peg setae closer to margins, removed from margins near base of rows, apex with dense field of peg setae (Fig. 17I). Female unknown.

##### Distribution.

Figure 20C. Known from the Himalaya of West Bengal, India.

##### Bionomics.

The holotype was collected in October at 200 m and was sifted from leaves in a forest ravine (Smetana 1988).

##### Etymology.

The species epithet refers to its distribution in the Himalayan mountains. *Bolitogyrus himalayicus* is the only described species known to occur in this region.

##### Comments.

This is the species Smetana (1988) figured as *B. vulneratus* (fig. 270-274). *Bolitogyrus himalayicus* is extremely similar to allopatric *B. nanus* from the Khasi Hills of Meghalaya but can be distinguished by the single-toothed apex of the median lobe and dense field of peg setae on the apex of the paramere.

#### Bolitogyrus nanus

Taxon classificationAnimaliaColeopteraStaphylinidae

Brunke sp. n.

http://zoobank.org/BF37C021-CD3F-40E5-8AC1-88F6F4AB6BC3

Fig. 17K–L , 20C (map)

##### Type locality.

Mawsynram-Balat Road, Khasi Hills, Meghalaya, India.

##### Type material.

**Holotype** (♂, MHNG): INDIA, Meghalaya, Khasi Hills, 1000m, Mawsynram-Balat, Besuchet-Löbl, 27.X.78, [1978] [printed] / Cyrtothorax spec. det. M. Uhlig 1982 [written] / Cyrtothorax vulneratus Smetana det. 1986 [written] / HOLOTYPE *Bolitogyrus nanus* Brunke, des. A. Brunke 2017 [red label] / AJB0000431 [identifier label].

##### Diagnosis.

This species can be distinguished by the following character states: head entirely dark; elytral disc dark with pale markings extending halfway to epipleural margin (Fig. 5G); pronotal margin at its widest point no more than three lateral puncture widths wide but still distinctly expanded at hind angles; forebody less than four millimeters; apex of median lobe with double-toothed carina or ‘lip’ (Fig. 17L); paramere with only simple rows of peg setae and without dense field (Fig. 17K).

##### Description.

Measurements ♂ (n = 1): HW/HL 1.40; PW/PL 1.29; EW/ EL 1.23; ESut/PL 0.84; PW/HW 1.01; forebody length 3.5 mm.

Extremely similar to *B. khasiensis* and differing only in the following: medial and lateral elytral markings more broadly connected, pale area of epipleuron not restricted to humeral spot, pale in up to entire basal half; pronotum slightly more transverse; elytral suture relatively longer than pronotum at middle; forebody distinctly shorter and thinner; median lobe in parameral view with apex distinctly acuminate and acute, apex with folded, double-tooted carina (Fig. 17L); paramere with sparse and simple rows of peg setae placed close to margin, removed from margin only at base of rows (Fig. 17K). Female unknown.

##### Distribution.

Figure 20C. Known only from the Khasi Hills of Meghalaya.

##### Bionomics.

The holotype was collected in October at 1000 m.

##### Etymology.

The species epithet refers to the fact that this species is the smallest known in the Oriental fauna at 3.5 millimeters in forebody length.

##### Comments.

*Bolitogyrus nanus* is extremely similar to allopatric *B. himalayicus* from West Bengal but is slightly smaller and the apex of the median lobe bears a double-toothed carina and the paramere lacks the dense apical field of peg setae.

#### Bolitogyrus pecki

Taxon classificationAnimaliaColeopteraStaphylinidae

Brunke sp. n.

http://zoobank.org/6B9D4CAC-9CAA-4750-9A03-9D2934768B53

(Fig. 17M-O , 22B (map))

##### Type locality.

BaBe National Park, Bac Kan Province, Vietnam.

##### Type material.

**Holotype** (♂, CNC): VIETNAM: Cao Bang [Bac Kan] Prov., BaBe Nat. Park, 7–11.V.97, 180 m / forest FITs, S. Peck, 97-2 / HOLOTYPE *Bolitogyrus pecki* Brunke, des. A. Brunke 2017 [red label] / CNC655574 [identifier label]

##### Diagnosis.

This species can be distinguished by the following character states: head entirely dark; elytral disc dark with pale markings extending halfway to epipleural margin (Fig. 5G), medial and lateral discal spots distinguishable and not forming distinct chevron or v-shaped marking or broad pale area; pronotal margin at its widest point no more than three lateral puncture widths wide but still distinctly expanded at hind angles; apex of hind femur with only vague dark marking on ventral margin; antennomere 7 distinctly transverse; paramere with distinct rows of peg setae (Fig. 17O).

##### Description.

Measurements ♂ (n = 1): HW/HL 1.40; PW/PL 1.31; EW/ EL 1.22; ESut/PL 0.83; PW/HW 1.06; forebody length 4.5 mm.

Similar to *B. khasiensis* and differing only in the following: medial and lateral elytral markings much more broadly connected, pale area of epipleuron not restricted to humeral spot, pale in up to entire basal half; abdomen slightly paler, dark reddish brown toward the base; head and pronotum more transverse; elytral suture relatively longer than pronotum at middle; forebody distinctly broader, appearing more robust; median lobe with laterally placed basal teeth, in parameral view with apex bearing folded, single-tooted carina (Fig. 17M); median lobe in lateral view only simple apical tooth, with basal teeth appearing removed from lateral margin (Fig. 17N); paramere with slender apical portion, with sparse marginal and medial rows of peg setae, apex with dense field placed close to margin, apical setae extremely small, almost unobservable at lower magnifications (Fig. 17O). Female unknown.

##### Distribution.

Figure 22B. Known only from northern Vietnam.

##### Bionomics.

The holotype was collected in May using an FIT in lowland forest (180 m).

##### Etymology.

This species is named in honor of Stewart Peck (Carleton University, Ottawa, Canada), the collector of its holotype and many other rare taxa belonging to Staphylinini.

##### Comments.

*Bolitogyrus pecki* is similar to the allopatric *B. hainanensis* from Hainan, China but can easily be distinguished by median lobe in lateral view, which is not recurved and the peg setae of the paramere, which are arranged in simple marginal and medial rows.

### Undescribed species of the Loculus Group

A single female specimen from Kachin State, Myanmar (NHMW) was studied that is similar to *B. smetanai* but lacks an orange frons and likely represents an undescribed species.

### Species *incertae sedis*

#### Bolitogyrus elegans

Taxon classificationAnimaliaColeopteraStaphylinidae

(Cameron, 1937)

Figs 2G , 4D , 4E , 18A-C , 20A (map)

Cyrtothorax elegans Cameron, 1937: 28.

##### Type locality.

Baturaden (“Batoerraden”), Mt. Slamet, Central Java.

##### Type material.

*Cyrtothorax elegans* Cameron, 1937.

**Syntypes** (2♂, 4♀, BMNH, FMNH): Batoerraden [=Baturaden], G. Slamat [=Slamet], Java, F.C. Drescher, VII.1928 [printed] / M. Cameron., Bequest., B.M. 1955-147 [printed] / SYNTYPE *Cyrtothorax elegans* Cameron, 1937 det. A. Brunke 2017 [red label] / AJB0000407 [identifier label].; same except: AJB0000509; same except Chicago Nat. Hist. Mus. (ex. M. Cameron Colln. by exchange with Brit. Mus. Nat. Hist.) [printed] / AJB0000508; same except, IX-1932, AJB0000510, AJB0000511; same except, 22-III-1930, AJB0000512.

##### Diagnosis.

This species can be recognized by the following combination of characters: orange frons contrasting with dark head (Fig. 2G); pronotal margin only weakly expanded, no more than three lateral puncture widths wide (Fig. 4D); apical antennomere distinctly paler than previous segments (Fig. 2G).

##### Redescription.

Measurements ♂ (n = 2): HW/HL 1.38–1.40; PW/PL 1.10–1.13; EW/ EL 1.16–1.21; ESut/PL 0.74–0.76; PW/HW 0.97–1.01; forebody length 4.1–4.4 mm.

Measurements ♀ (n = 4): HW/HL 1.35–1.38; PW/PL 0.99–1.03; EW/ EL 1.22–1.23; ESut/PL 0.75–0.79; PW/HW 0.99–1.03; forebody length 4.2–4.7 mm.

Coloration: head dark with orange frons; pronotum varying from entirely reddish-orange, to with median and pair of darkened areas; elytra dark with humeri, apical angles and v-shaped , raised discal marking light yellow, base and area along suture orange, these pale areas often connecting to leave only outer apical areas dark; scutellum dark to reddish with dark border; antennomere 1 yellow with darkened apex, 2-5 light brownish-red, 6-10 dark brown, apical segment distinctly paler, light brown to yellow; palpi yellow-brown; legs yellow, on ventral face, very apex of femur darkened.

Head distinctly transverse, dorsal surface with moderately dense, clearly separated asetose punctures, frons with very few scattered punctures and shallow Y-shaped impression. Antennomeres 6 slightly, 7-10 distinctly transverse, 10 more elongate than 9, 7-10 slightly asymmetrical.

Pronotum slightly transverse to quadrate, about as wide as head, with scattered shallow micropunctures on disc, becoming more distinct on anterior angles. Elytra slightly transverse, suture distinctly shorter than pronotum at middle, punctation relatively sparse, with most punctures separated by a distance not less than their diameter.

Abdomen with disc of tergites III-VI distinctly impunctate medially, basal impressions with elongate punctures; sternites III-IV with basal line distinctly projected posteriad at middle.

Median lobe in lateral view with apical fourth more strongly narrowed to apex, which is deflexed ventrad (Fig. 18B); median lobe in parameral view with basal teeth appearing medially, apical portion elongate triangular and slightly to strongly acuminate (Fig. 18A); paramere distinctly longer than median lobe, widest at about midlength, with long apical portion distinctly curved dorsad over apex of median lobe in lateral view (Fig. 18B-C); peg setae arranged in single row of variable density, basal-most setae larger and sometimes duplicated, rows removed from margin and at most only weakly converging (Fig. 18C); male sternite VIII with shallow emargination and triangular glabrous area medially; male sternite IX moderately expanded at midlength, with distinct, deep emargination.

Female tergite VIII with median emargination varying from small and triangular to moderately deep and elongate triangular; female tergite X elongate, with acute, acuminate apex; disc only weakly raised in apical half, not separated by ridges.

##### Distribution.

Figure 20A. Known only from the type locality on Mt. Slamet, Java.

##### Bionomics.

Specimens were collected in March and July.

##### Comments.

*Bolitogyrus elegans* is externally most similar to allopatric *B. smetanai* (Thailand, Myanmar) but is easily distinguished by the pale apical antennomere and the more sparsely punctate elytra.

#### Bolitogyrus ornatipennis

Taxon classificationAnimaliaColeopteraStaphylinidae

(Wendeler, 1927) comb. n.

Fig. 5F , 18D-F , 20A (map)

Quedius (Raphirus) ornatipennis Wendeler, 1927: 9.
Cyrtothorax octomaculatus Cameron, 1937: 28 **syn. n.**

##### Type locality.

Mount Kendang, West Java.

##### Type material.

*Quedius ornatipennis* Wendeler, 1927.

**Holotype** (♀, ZMHB): Kendeng-Gebirge [=Mt. Kendang], O. Java, A. Heyne, Berlin Wilm, I [printed] / *Cyrtothorax* [sic] *ornatipennis* n.sp. Wendeler det. [handwritten] / Holotypus [red label] / ♀ [printed] / Bolitogyrus ms name Det. Chatzimanolis 2010 [printed] / HOLOTYPUS Quedius (Raphirus) ornatipennis Wendeler, 1927, labelled by MNHUB 2012 [red label] /AJB0000408 [identifier label].

##### Type material.

*Cyrtothorax octomaculatus* Cameron, 1937 syn. n.

**Syntypes** (1♂, 3♀, BMNH, FMNH): F.C. Drescher, G. Tangkoeban Prahoe [=Tangkuban Perahu], 4000-5000, Voet, Preanger, Java, 19-31.I.1933 [printed w. written date] / M. Cameron. Bequest., B.M. 1955-147 [printed] / SYNTYPE *Cyrtothorax octomaculatus* Cameron, det. A. Brunke 2017 [red label] / *Bolitogyrus ornatipennis* (Wendeler) det. A. Brunke 2017 [printed] / AJB0000409 [identifier label]; same except, VIII-1933 / Chicago Nat. Hist. Mus. (ex. M. Cameron Colln. by exchange with Brit. Mus. Nat. Hist.) [printed] / AJB0000513; same except, 20-VII-1933 / AJB0000514; same except, 2-VIII-1933 / AJB0000515.

##### Other material.

**INDONESIA**: Java: *West Java*: ‘Tjobidai’ [=Cibodas, botanical garden], J. Skovgaard, 1 ♂, AJB0000516 (ZMUC); Gede-Pangrango Nat. P., Selabintana gate to Sawer Waterfall, 1000-1200 m, 23.VIII.1994, Schuh, 1 ♀, AJB0000517 (NMW); same except, way to Cibeureum waterfall, 1500-1620 m, 2-3.VIII.1994, R. Schuh, 1 ♀, AJB0000519 (NMW); Tangkuban Perahu, VI.1933, v. Doesburg, 1 ♂, AJB0000518 (NMW); Rancas Upas, 10 km S Ciwidey, ‘ca. 1300 m’ [actually ca. 1700 m], 9.VIII.1994, leg. Schuh, 1 ♂, AJB0000520 (NMW).

##### Diagnosis.

This species can be distinguished by the following character states: head entirely dark; elytral disc dark with pale markings not extending onto epipleuron (Fig. 5F); pronotal margin at its widest point no more than three lateral puncture widths wide but still distinctly expanded at hind angles (Fig. 5F); hind tibia at least partly darker than pale region of femur; head with discal punctures and frontal impression extremely shallow.

##### Redescription.

Measurements ♂ (n = 4): HW/HL 1.33–1.38; PW/PL 1.10–1.20; EW/ EL 1.14–1.19; ESut/PL 0.84–0.86; PW/HW 0.95–0.99; forebody length 3.8–4.4 mm.

Measurements ♀ (n = 5): HW/HL 1.32–1.40; PW/PL 1.13–1.23; EW/ EL 1.15–1.19; ESut/PL 0.82–0.90; PW/HW 0.94–1.04; forebody length 4.4–4.6 mm.

Similar to *B. elegans* and differing only in the following: head and pronotum entirely dark; elytral dark, v-shaped marking often broken into two markings, outer marking usually elongate and inner marking loosely triangular (Fig. 5F); leg color variable, coxae almost entirely pale to entirely dark, femora yellow with dark apical band or with only small ventroapical spot, tibia entirely dark to light brownish in faded specimens; antennomeres 6-10 more transverse, 10 not more elongate than 9; disc of head with punctures smaller and far more shallow; pronotum more variable in shape but slightly more transverse, elytral suture longer relative to pronotum at middle; basal impressions of abdomen with elongate to regular punctures, depending on specimen; paramere slightly longer than median lobe, with long apical portion not projected over apex of median lobe (Fig. 18E); peg setae rows strongly convergent (Fig. 18F); female tergite X with longer apical projection.

##### Distribution.

Figure 20A. Known from the mountains encircling the Bandung City area of West Java.

##### Bionomics.

Specimens have been collected in January and June-August, at elevations ranging from 1000-1700 m.

##### Comments.

*Bolitogyrus ornatipennis* is externally most similar to allopatric *B. lasti* (southern India) but can be distinguished by the more triangular median elytral marking and the distinct expansion of the pronotal margin.

#### Bolitogyrus doesburgi

Taxon classificationAnimaliaColeopteraStaphylinidae

(Scheerpeltz, 1974)

Figs 18G–I , 20A (map)

Cyrtothorax doesburgi Scheerpeltz, 1974: 187.

##### Type locality.

Mt. Muria (“Gn. Moeria”), Central Java.

##### Type material.

*Cyrtothorax doesburgi* Scheerpeltz, 1974.

**Holotype** (♂, NMW): P.H. v. Doesburg, Java: Gg Moeria [Muria], Tjolo, 700–1000 m, 12-II-1913 [printed] / Cyrtothorax doesburgi Scheerpeltz [handwritten] / Doesburgi Scheerpeltz [handwritten] / TYPUS Cyrtothorax doesburgi O. Scheerpeltz [red label] / ALLOTYPUS [red label] / ♀ [printed] / coll. Scheerpeltz [blue printed label] / AJB0000393 [identifier label].

Scheerpeltz (1974) stated that his specimen was a female, when actually it is a male.

##### Diagnosis.

*Bolitogyrus doesburgi* is easily recognized by the uniformly reddish elytra and the minutely expanded pronotal margin.

##### Redescription.

Measurements ♂ (n = 1): HW/HL 1.32; PW/PL 1.30; EW/ EL 1.19; ESut/PL 0.89; PW/HW 0.98; forebody length 6.0 mm.

Coloration: body dark; elytra reddish; abdominal tergite III mostly dark with lateroapical areas reddish, IV with middle dark and remaining area reddish, V entirely reddish, VI-VII dark with base and apex reddish, VIII dark; antennomere 1 yellow, II-V reddish, 6-10 dark brown, 11 pale yellow; palpi yellowish, apical segment darkened; legs yellowish, femora with dorsal surface darkened.

Head distinctly transverse; dorsal surface with moderately dense, clearly separated asetose punctures, frons with coarse depressions in addition to usual frontal impressions and with scattered punctures, frons bearing a pair of setose punctures between anterior frontal punctures. Antennomeres 7-10 transverse and asymmetrical.

Pronotum distinctly transverse, convex and with micropunctures scattered on disc, becoming rather dense on anterior angles. Elytra slightly transverse, slightly shorter than pronotum at middle, in addition to usual macrosetal rows on disc, scattered punctures bearing setae, nearly all punctures setose on epipleuron of elytron.

Abdomen with disc of tergites III-V distinctly impunctate; sternites III-IV with basal line distinctly projected posteriad at middle.

Median lobe in lateral view gradually narrowed apicad to level of single median, apical tooth formed from projection of apical carina, apex of median lobe slightly constricted apicad of tooth, median lobe with pair of basal teeth at about midlength (Fig. 18G); median lobe in parameral view elongate, asymmetrically sinuate in apical half (Fig. 18H); paramere slightly longer than median lobe, apical half leaf-shaped and slightly asymmetrical, peg setae arranged in thin marginal group and sparse medial group, marginal group broadens at apex into a pair of triangular shapes, median group consisting of a paired row extending basad of marginal group (Fig. 18I); male sternite VIII with shallow emargination and triangular glabrous area medially; male sternite IX moderately expanded at midlength, with distinct emargination.

Female unknown.

##### Distribution.

Figure 20A. Known only from the type locality on Mt. Muria, Central Java.

##### Bionomics.

The holotype was collected in February at 700-1000 m.

##### Comments.

*Bolitogyrus doesburgi* is the only Oriental species of the genus with an asymmetrical aedeagus.

#### Bolitogyrus signatus

Taxon classificationAnimaliaColeopteraStaphylinidae

(Cameron, 1932)

Figs 2H–I , 18J–L , 20D (map)

Cyrtothorax signatus Cameron, 1932: 278.

##### Type locality.

Dikoya, Central Province, Sri Lanka.

##### Type material.

*Cyrtothorax signatus* Cameron, 1932.

**Holotype** (♂, BMNH): Type [circle label with red border] / Dikoya, 3,800–4,200 ft., 6.XII.81–16.I.82 [printed] / Ceylon, G. Lewis, 1910-320. [printed] / Cyrtothorax signatus Cam TYPE [written] / HOLOTYPE *Cyrtothorax signatus* Cameron, det. A. Brunke 2017 [red label] / AJB0000438 [identifier label].

##### Other material.

**SRI LANKA**: *Sabaragamuwa*: ‘Sinharaja jungle’, in malaise trap, 10.IX.1979, M Kosztarab, T. Wijesinhe and L. Jayawickrema, 1 ♀, AJB0000579 (USNM).

##### Diagnosis.

*Bolitogyrus signatus* is easily recognized by the pronotum, which is widest at the anterior angles (Fig. 2H-I). This species (especially the male) bears a remarkable resemblance to the Neotropical species of the genus.

##### Redescription.

Measurements ♂ (n = 1): HW/HL 1.63; PW/PL 1.66; EW/ EL 1.23; ESut/PL 0.88; PW/HW 1.09; forebody length 5.1 mm.

Measurements ♀ (n = 1): HW/HL 1.55; PW/PL 1.21; EW/ EL 1.24; ESut/PL 0.71; PW/HW 0.97; forebody length 4.7 mm.

Coloration: body dark; head, pronotum and abdomen entirely dark, elytra dark, each with yellow v-shaped marking; antennomere 1 yellow with darkened apex, 2-5 reddish-orange with darkened apices, 6-10 dark brown, 11 pale yellow; palpi yellowish with apical segment darkened; forecoxae yellow with basal fifth dark brown in both sexes, legs yellow, forefemur with dorsal surface dark brown, lateral face and dorsal surface of mid and hind tibia with subapical dark marking, tibia with lateral face darker.

Head strongly transverse, more so in male; dorsal surface including frons glossy with sparse, small and poorly impressed punctures; in male, lateral part of head beneath eye expanded ventrad. Antennomeres 8-10 distinctly transverse but not asymmetrical.

Pronotum transverse and widest at anterior angles, strikingly more strongly transverse in males than females, with lateral portions explanate in males, protuberance moderate (male) or distinct (female); medial part of disc almost entirely without micropunctures, anterior angles with many impressed micropunctures. Elytra slightly transverse, suture distinctly to slightly shorter than pronotum at middle, nearly all punctures of epipleuron setose.

Abdomen with disc of tergites III-VI distinctly impunctate.

Median lobe in lateral view evenly converging to apex, ventral face nearly straight except for very apex flexed ventrad, without teeth (Fig. 18K); median lobe in parameral view slightly dilated to apical fourth, spoon-shaped with slightly acuminate apex (Fig. 18J); paramere distinctly longer than median lobe, dilated about midlength and weakly narrowed to elongate, parallel apical portion with truncate apex (Fig. 18L); paramere entire but with median suture to almost midlength, peg setae with thin marginal group, peg setae also scattered to each side of the midline (Fig. 18L); male sternite VIII with shallow but distinct emargination and triangular glabrous area medially; male sternite IX moderately expanded at midlength, with distinct emargination, partly fused to laterotergal sclerites at midlength.

Female with tergite VIII bearing large, circular emargination (Fig. 2I), tergite X shield-shaped with raised area elongate trapezoidal and without depression, apex truncate with acutely projected middle; gonocoxae with bases fused medially.

##### Distribution.

Figure 20D. Likely endemic to Sri Lanka.

##### Bionomics.

Specimens were collected in September and December-January at 1160–1280 m. One specimen was collected by a malaise trap.

##### Comments.

*Bolitogyrus signatus* is the only Oriental species of the genus to exhibit strong sexual dimorphism, as in some Neotropical species. The sclerites of the male and female genital segments are uniquely fused in this species.

### Undescribed species near incertae sedis taxa

Two female specimens from Java are similar to *B. doesburgi* but may each represent a different species. One specimen was collected in lowland forest (locality illegible, 100 m, RMNH) and the other from the Pangalengan area of West Java (1200 m, BMNH).

**Figure 8.**
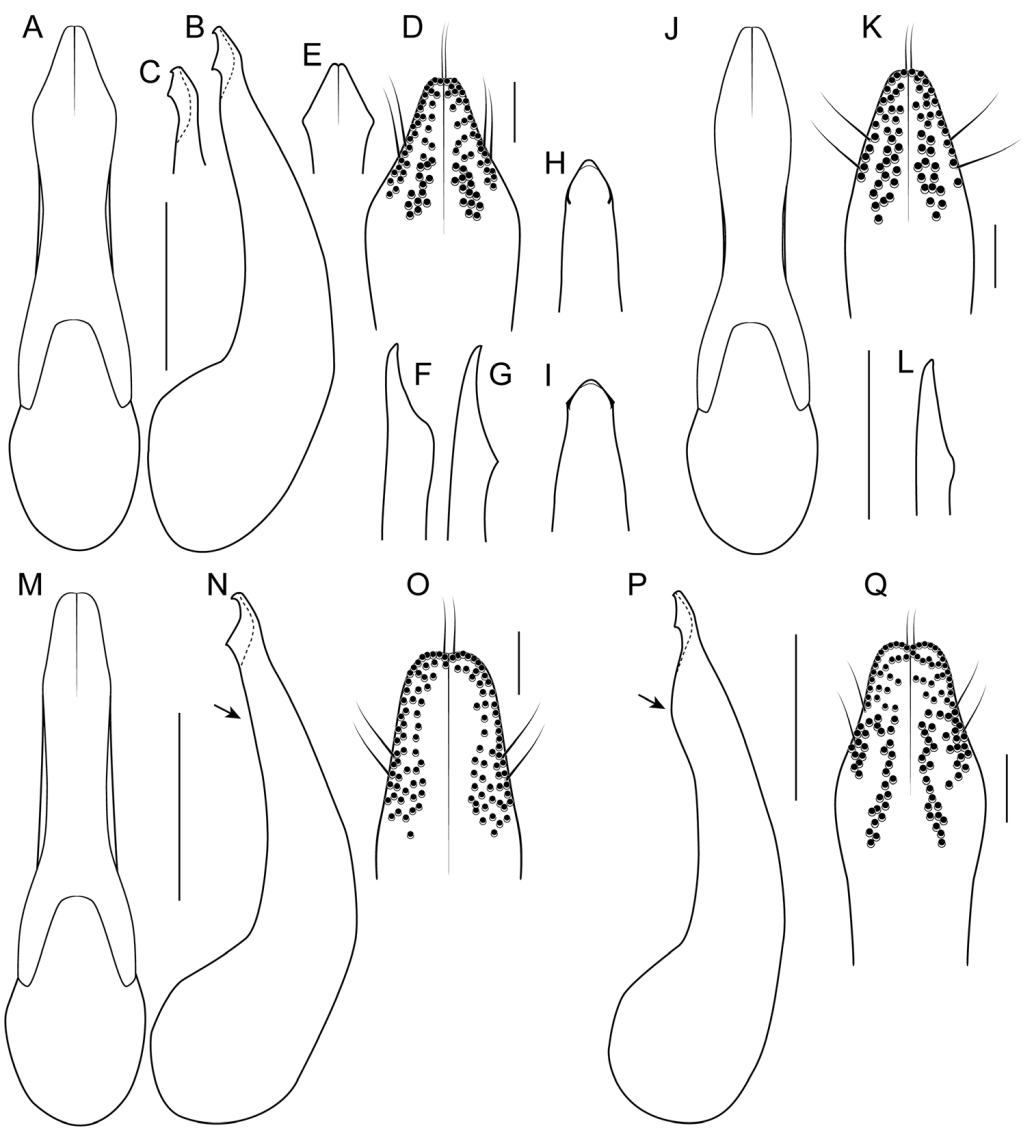
*Bolitogyrus electus* Smetana & Zheng (**A, B, D, F, H**), *B. uncus* Cai et al. (**C, E, G, I**), *B. confusus* Brunke (**J–L**), *B. huanghaoi* Hu et al. (**M–O**) and *B. nigropolitus* Smetana (**P, Q**). Aedeagus in parameral view (**A, J, M**), median lobe in lateral view (**B, C, N, P**), peg setae of paramere (**D, K, O, Q**), apex of paramere in lateral (**F, G, L**) and parameral view (**E**), apex of median lobe in parameral view (**H, I**). Figure 8E modified from Cai et al. (2015). Scale bars: 0.5 mm (**A–C, E–J**, **L–N, P**), 0.1 mm (**D, K, O, Q**).

**Figure 9.**
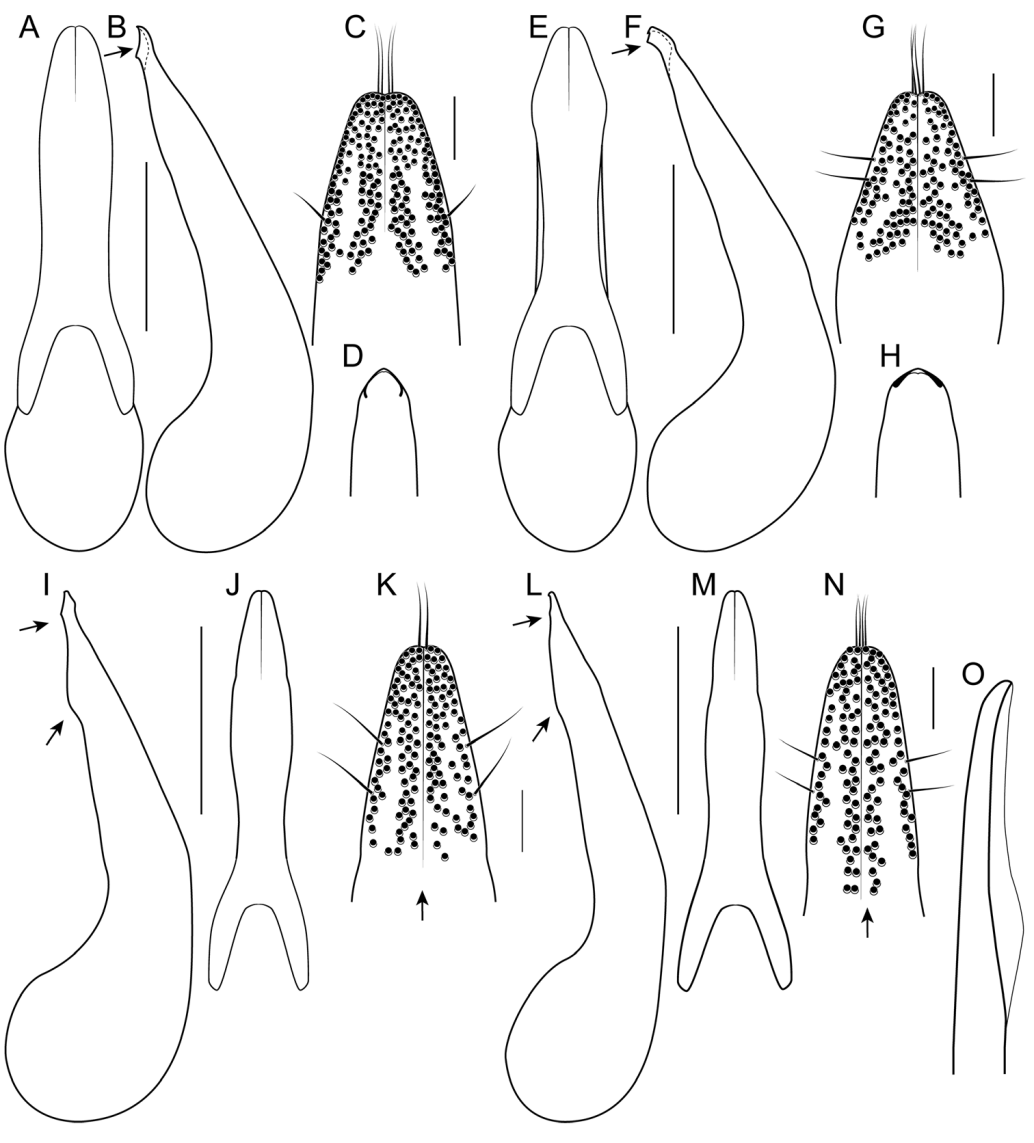
*Bolitogyrus cyanipennis* (Zheng) (**A–D**), *B. kitawakii* Smetana & Zheng (**E–H**), *B. nigerrimus* Yuan et al. (**I–K**) and *B. metallicus* Cai et al. (**L–O**). Aedeagus in parameral view (**A, E**), median lobe in lateral view (**B, F, I, L**), peg setae of paramere (**C, G, K, N**), apex of median lobe in parameral view (**D, H**), paramere (**J, M**), apical half of paramere in lateral view showing median ridge (**O**). Figures **A–D**, **M–O** modified from Cai et al. (2015). Scale bars: 0.5 mm (**A, B, D–F, H–J, L, M**), 0.1 mm (**C, G, K, N**).

**Figure 10.**
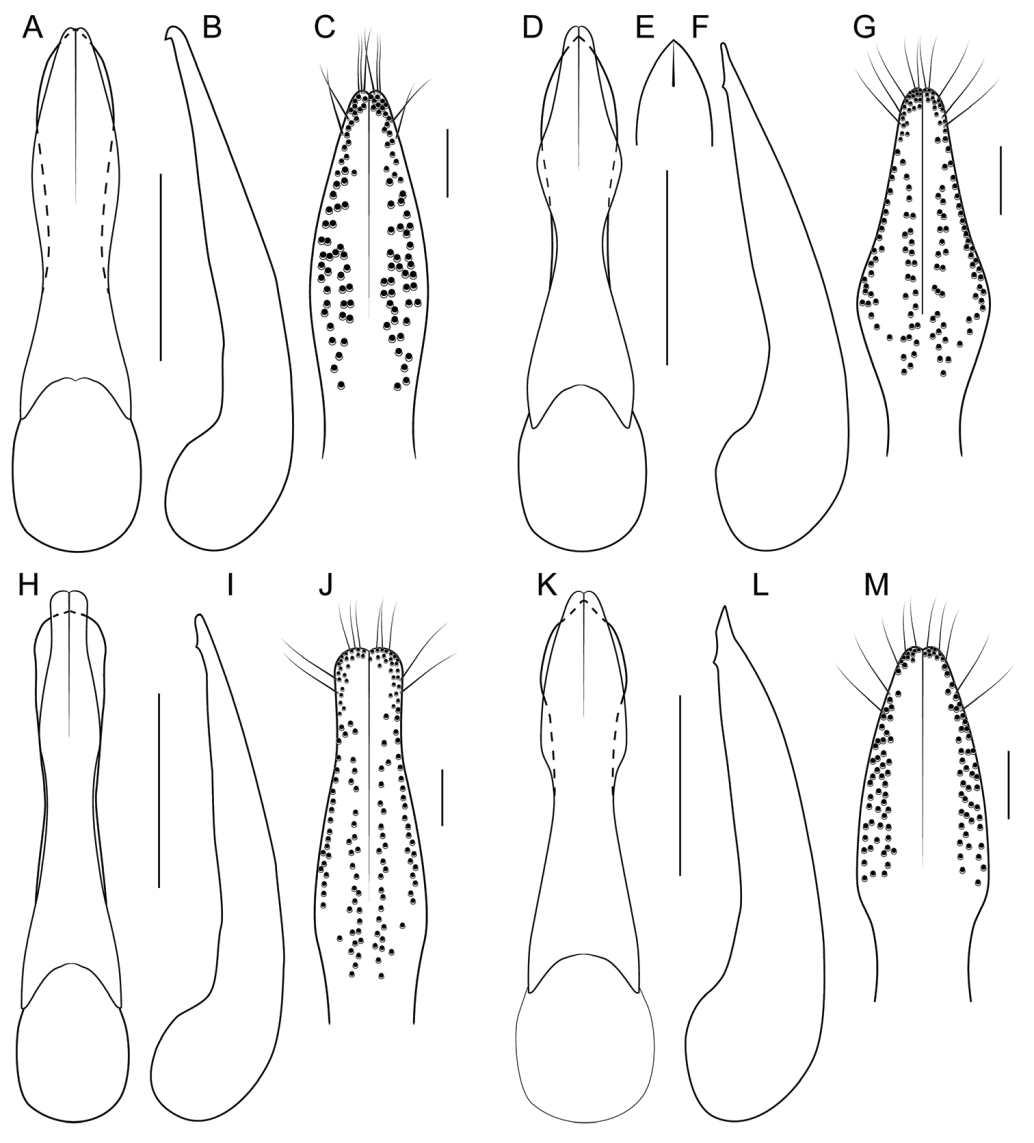
*Bolitogyrus caesareus* (Bernhauer) (**A–C**), *B. proximus* (Cameron) (**D–G**), *B. temburong* (Brunke) (**H–J**) and *B. rufipennis* (Cameron) (**K–M**). Aedeagus in parameral view (**A, D, H, K**), median lobe in lateral view (**B, F, I, L**), peg setae of paramere (**C, G, J, M**). Scale bars: 0.5 mm (**A–B, D–F, H–I, K–L**), 0.1 mm (**C, G, J, M**).

**Figure 11.**
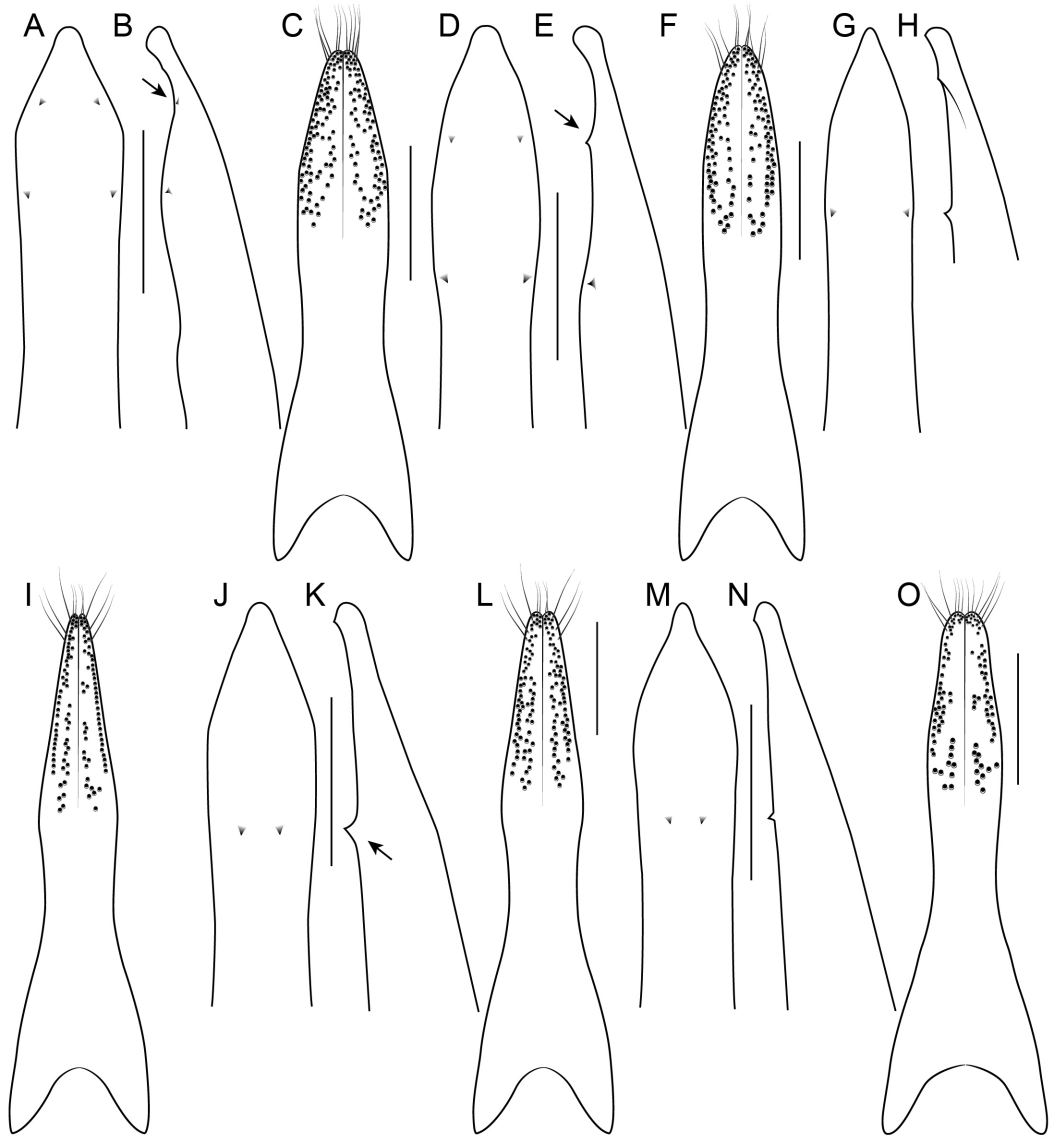
*Bolitogyrus carnifex* (Fauvel) (**A–C**), *B. pederseni* Brunke (**D–F**), *B. vietnamensis* (Scheerpeltz) (**G–I**), *B. elegantulus* Yuan et al. (**J–L**) and *B. phukhieo* Brunke (**M–O**). Median lobe in parameral view (**A, D, G, J, M**), median lobe in lateral view (**B, E, H, K, N**), peg setae of paramere (**C, F, I, L, O**). Scale bars: 0.5 mm.

**Figure 12.**
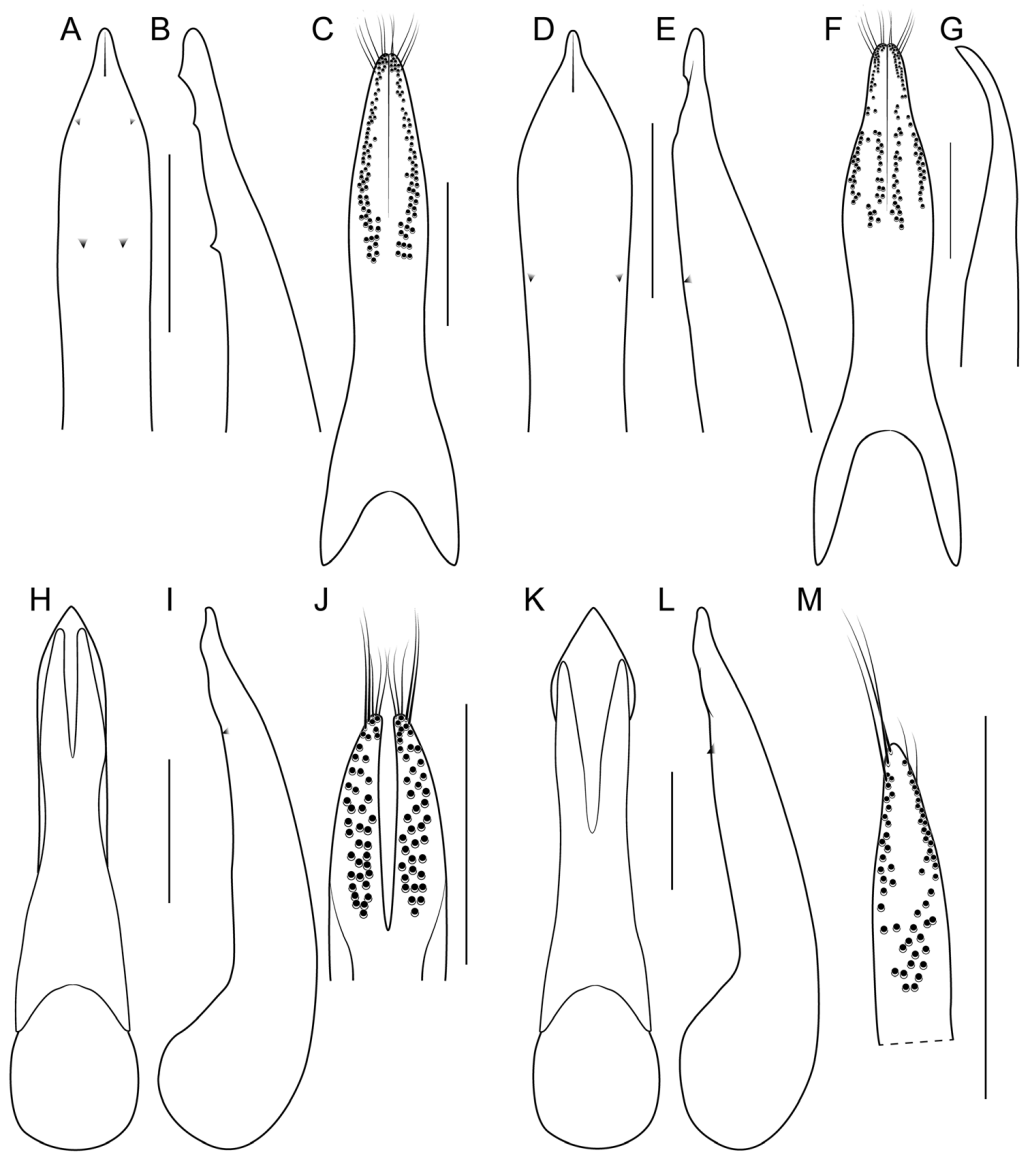
*Bolitogyrus magnimaculosus* Cai et al. (**A–C**), *B. nokrek* Brunke (**D–G**), *B. lasti* Rougemont (**H–J**) and *B. tigris* Brunke (**K–M**). Aedeagus in parameral view (**H, K**), median lobe in parameral view (**A, D**), median lobe in lateral view (**B, E, I, L**), peg setae of paramere (**C, F, J, M**), paramere in lateral view (**G**). Figures **A–C** modified from Cai et al. (2015). Scale bars: 0.5 mm.

**Figure 13.**
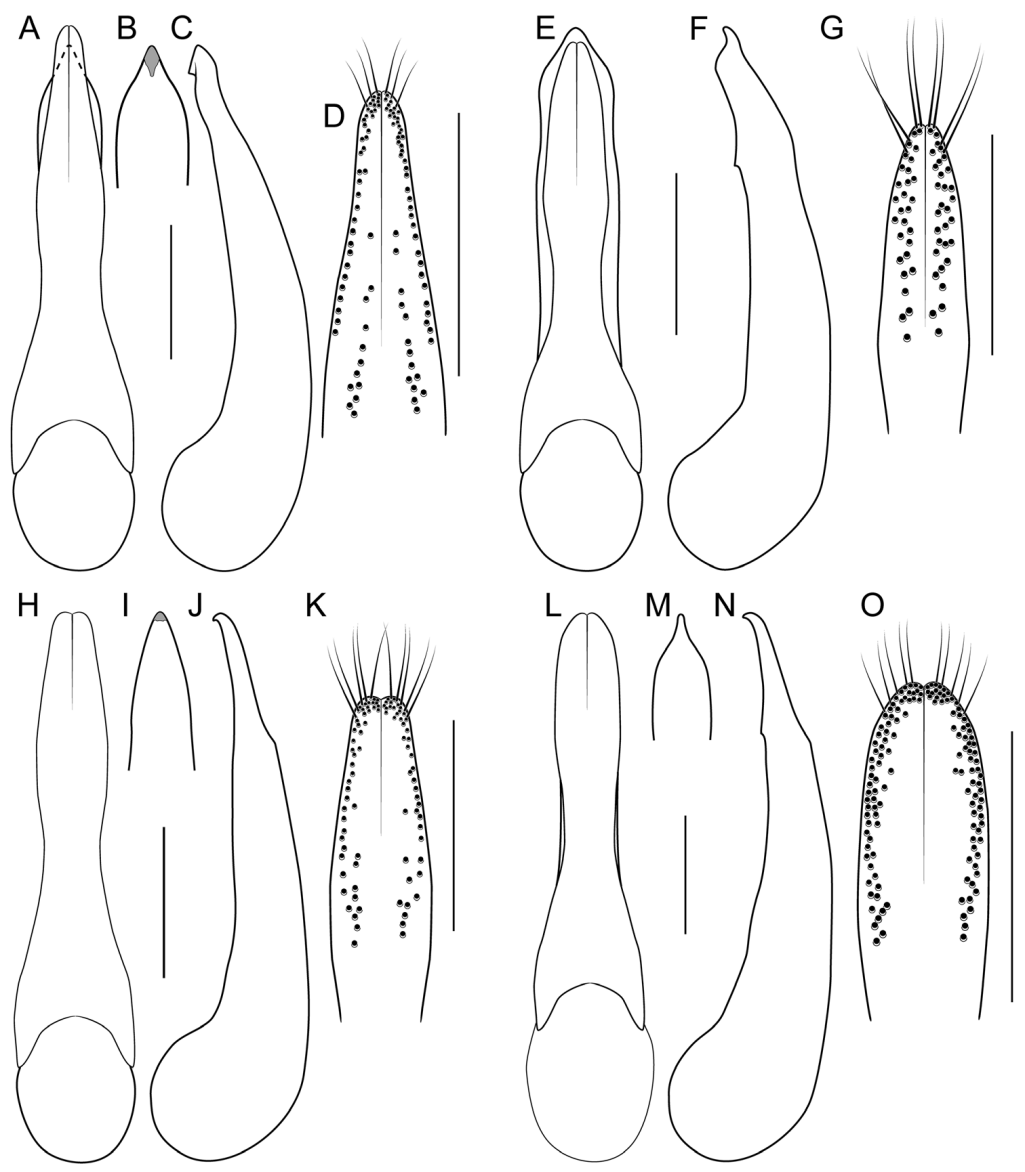
*Bolitogyrus luteus* Brunke (**A–D**), *B. sepilok* Brunke (**E–G**), *B. vulneratus* (Fauvel) (**H–K**) and *B. flavus* Yuan et al. (**L–O**). Aedeagus in parameral view (**A, E, H, L**), apex of median lobe in parameral view (**B, I, M**), median lobe in lateral view (**C, F, J, N**), peg setae of paramere (**D, G, K, O**). Scale bars: 0.5 mm.

**Figure 14.**
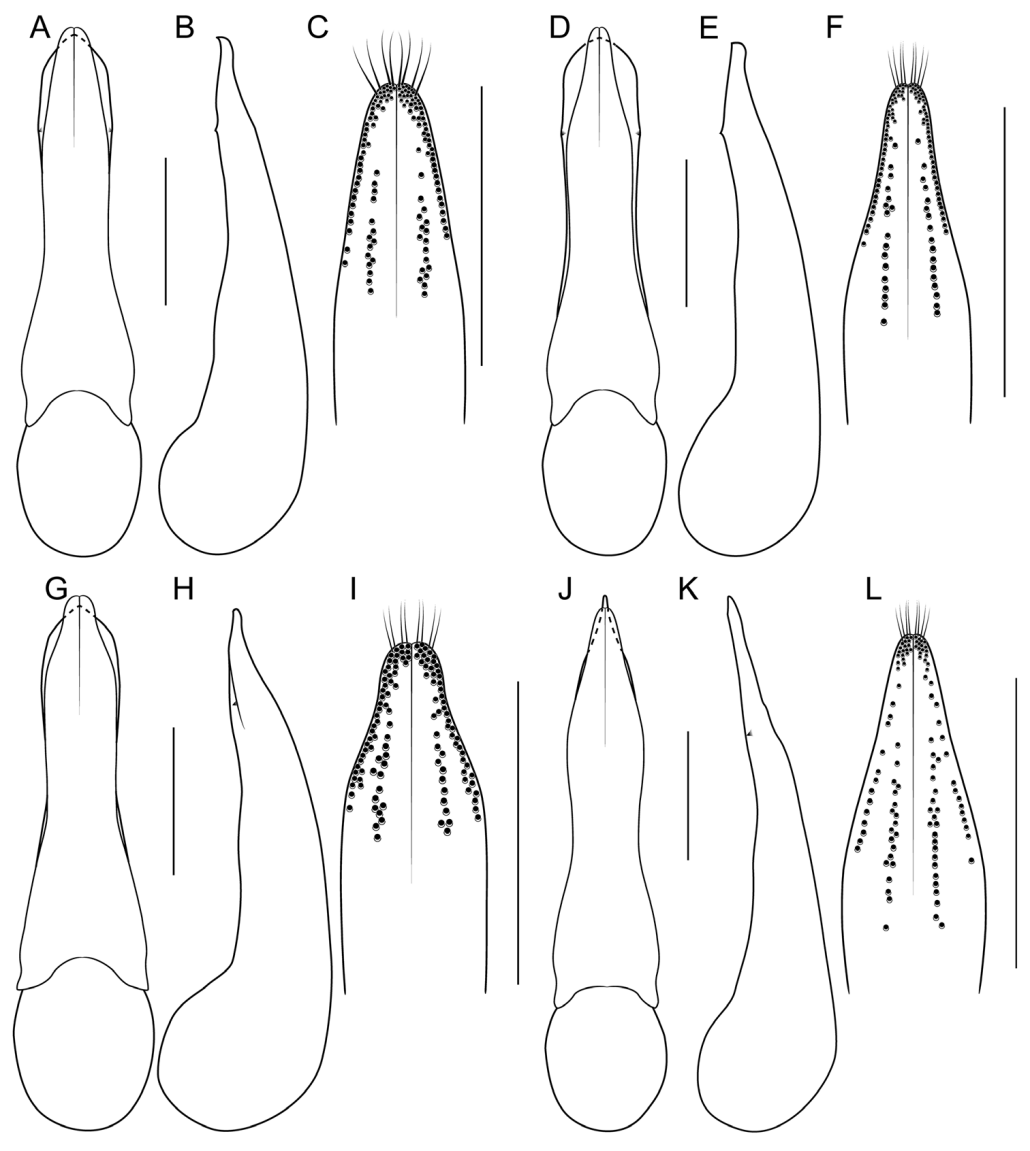
*Bolitogyrus pictus* Smetana & Zheng (**A–C**), *B. schillhammeri* Brunke (**D–F**), *B. rougemonti* Brunke (**G–I**) and *B. profundus* Cai et al. (**J–L**). Aedeagus in parameral view (**A, D, G, J**), median lobe in lateral view (**B, E, H, K**), peg setae of paramere (**C, F, I, L**). Scale bars: 0.5 mm.

**Figure 15.**
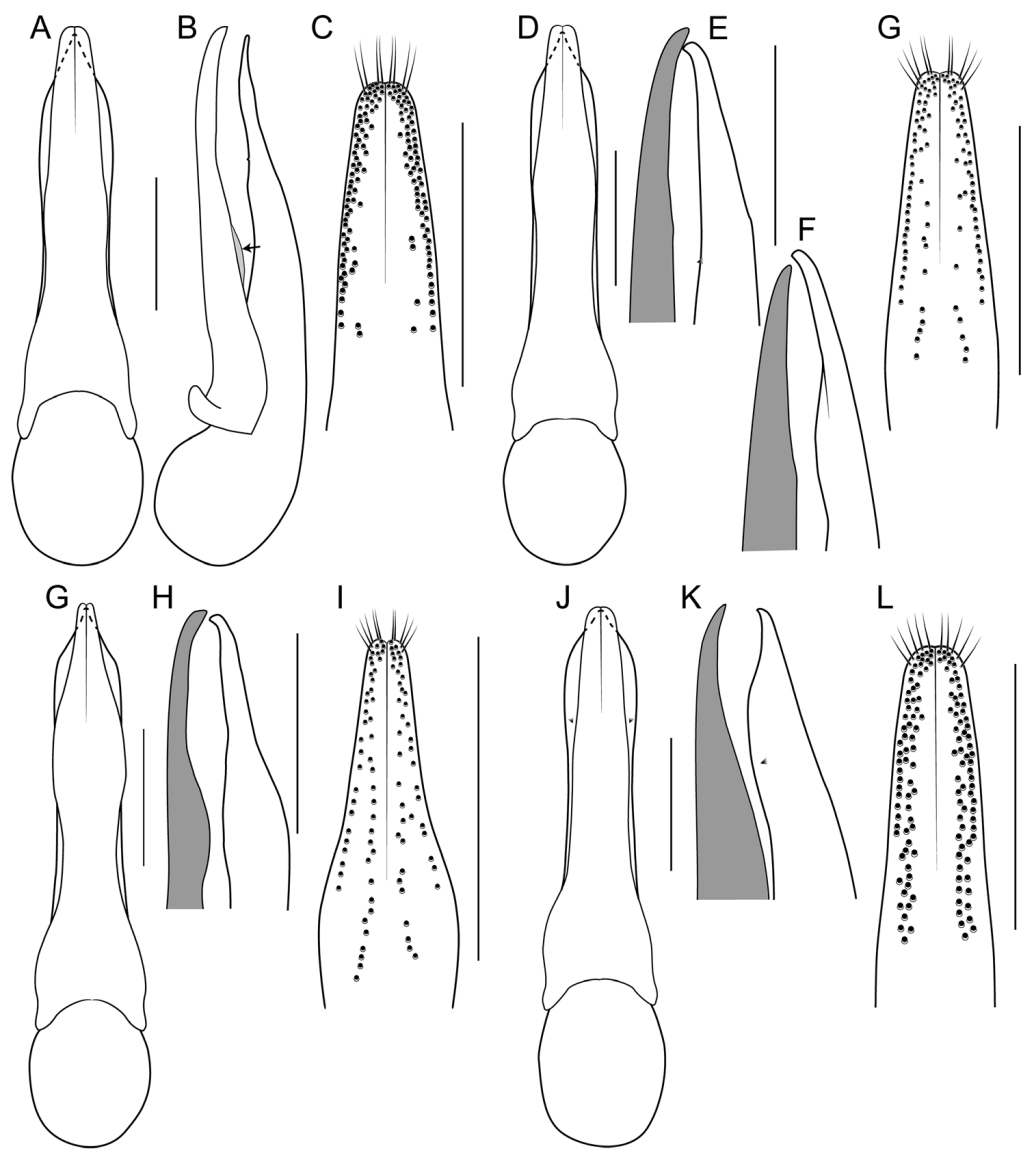
*Bolitogyrus concavus* Brunke (**A–C**), *B. rufomaculatus* (Shibata) (**D, E, G**), *B. depressus* Cai et al. (**F**), *B. taiwanensis* (Hayashi) (**G–I**) and *B. tumidus* Brunke (**J–L**). Aedeagus in parameral view (**A, D, G, J**), aedeagus in lateral view showing expansion of the paramere (arrow) (**B**), apex of aedeagus in lateral view (**E, F, H, K**), peg setae of paramere (**C, G, I, L**). Scale bar: 0.5 mm.

**Figure 16.**
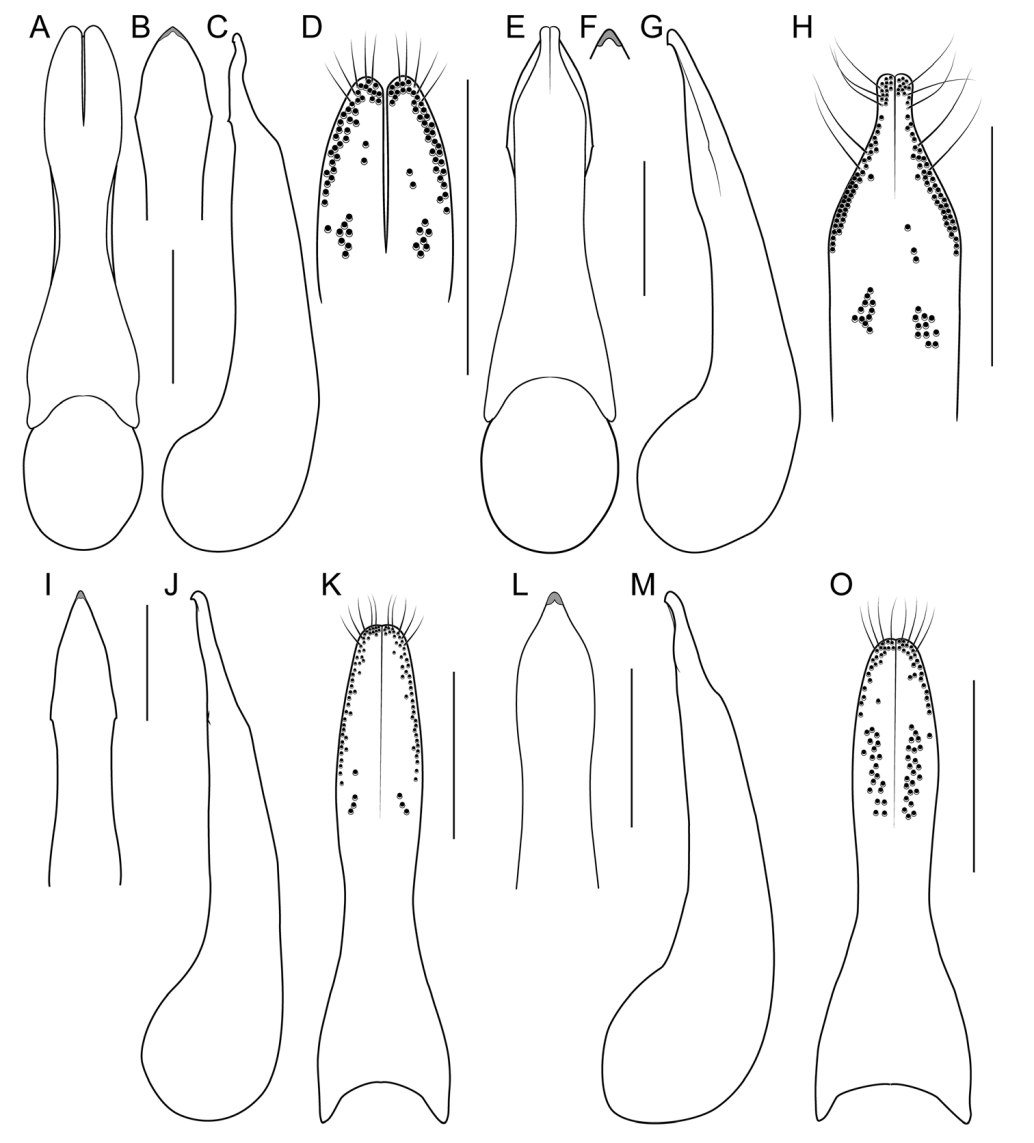
*Bolitogyrus hainanensis* Cai et al. (**A–D**), *B. loculus* Cai et al. (**E–H**), *B. solodovnikovi* Brunke (**I–K**) and *B. feai* Brunke (**L–O**). Aedeagus in parameral view (**A, E**), apex of median lobe in parameral view (**B, F, I, L**), median lobe in lateral view (**C, G, J, M**), peg setae of paramere (**D, H, K, O**). Figures 16A, B, D, E, F, H modified from Cai et al. (2015). Scale bars: 0.5 mm.

**Figure 17.**
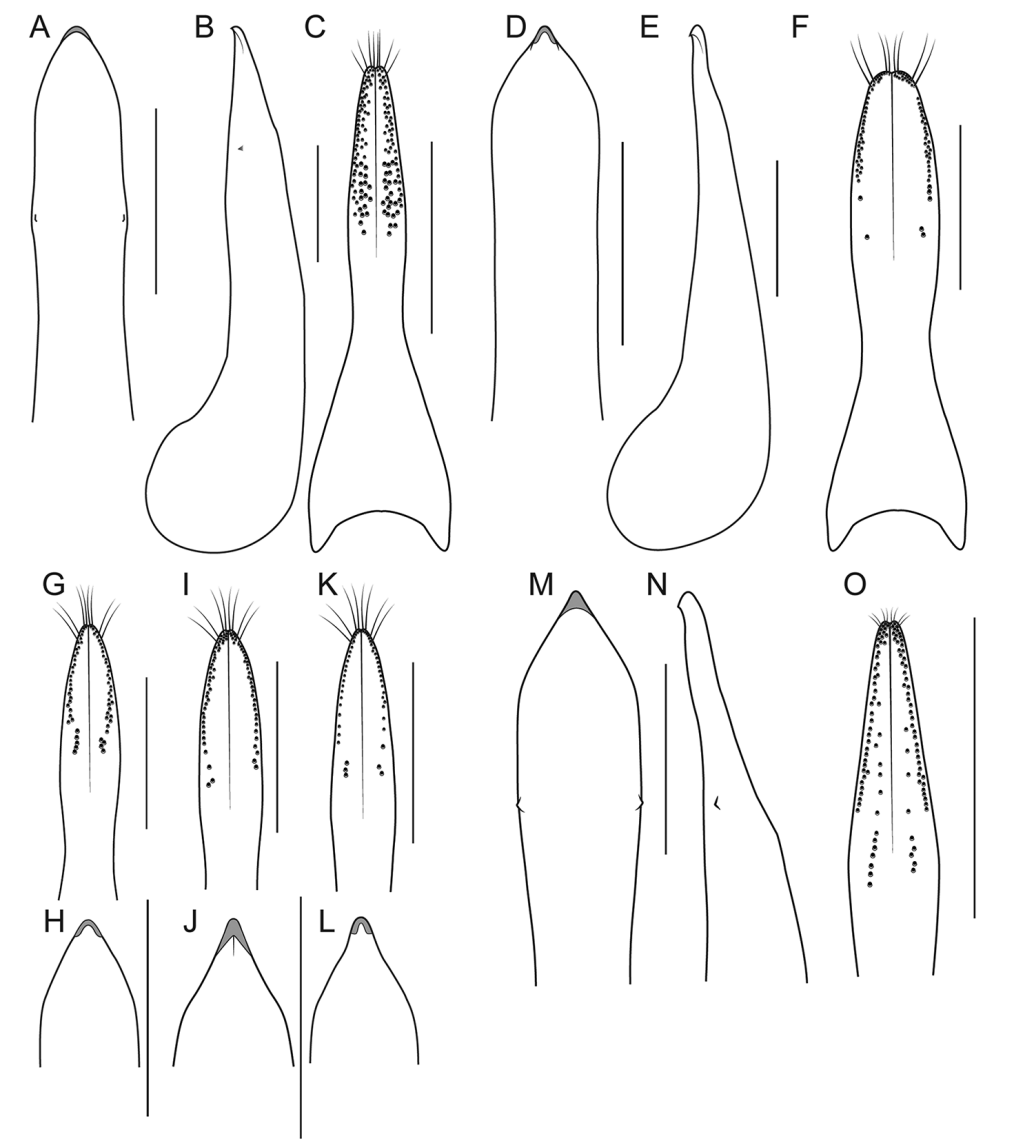
*Bolitogyrus mulayitensis* Brunke (**A–C**), *B. smetanai* Brunke (**D–F**), *B. khasiensis* Brunke (**G–H**), *B. himalayicus* Brunke (**I–J**), *B. nanus* Brunke (**K–L**), and *B. pecki* Brunke (**M–O**). Median lobe in parameral view (**A, D, H, J, L, M**), median lobe in lateral view (**B, E, N**), peg setae of paramere (**C, F, G, I, K, O**). Scale bars: 0.5 mm.

**Figure 18.**
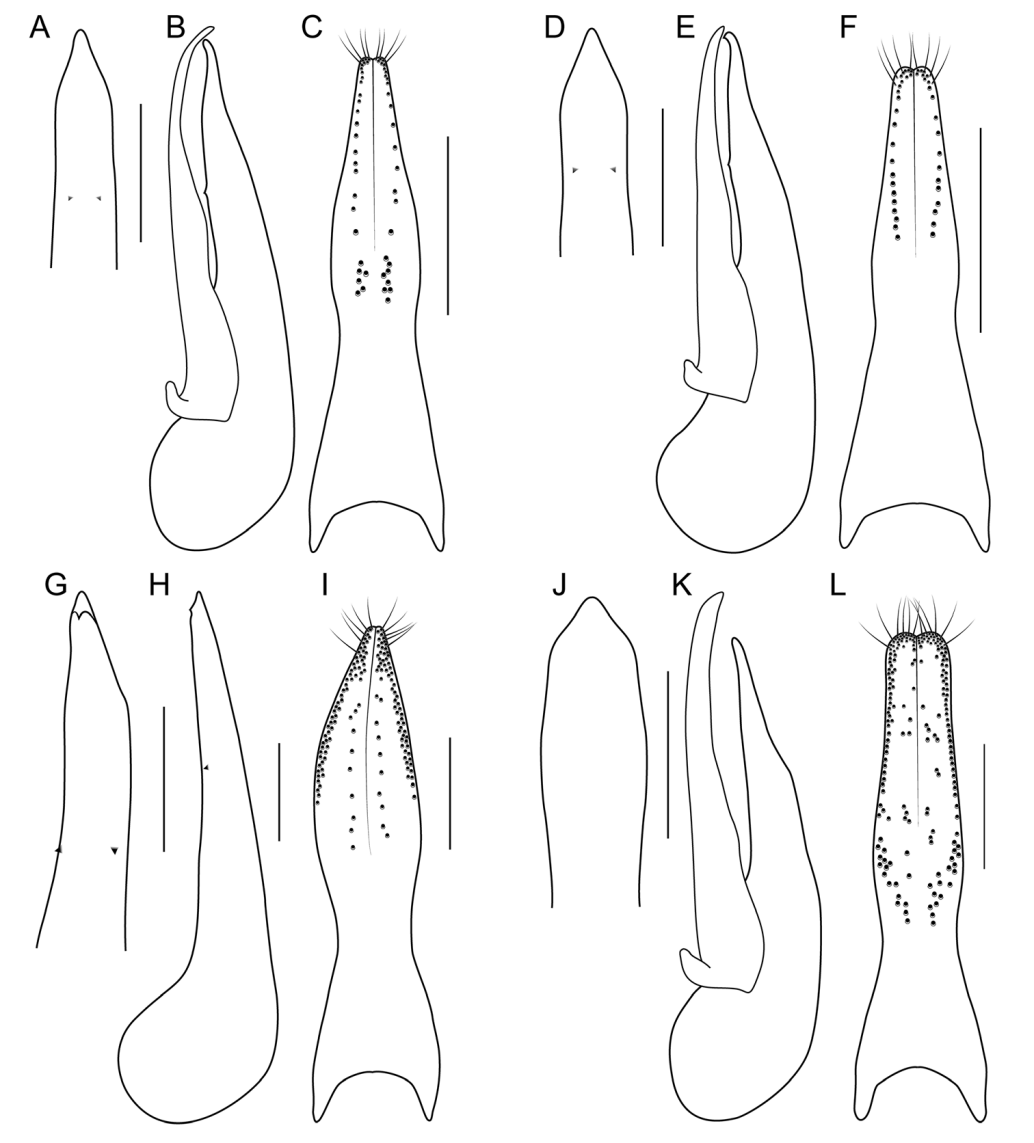
*Bolitogyrus elegans* (Cameron) (**A–C**), *B. ornatipennis* (Wendeler) (**D–F**), *B. doesburgi* (Scheerpeltz) (**G–I**) and *B. signatus* (Cameron) (**J–L**). Median lobe in parameral view (**A, D, G, J**), median lobe in lateral view (**H**), aedeagus in lateral view (**B, E, K**), peg setae of paramere (**C, F, I, L**). Scale bars: 0.5 mm.

**Figure 19.**
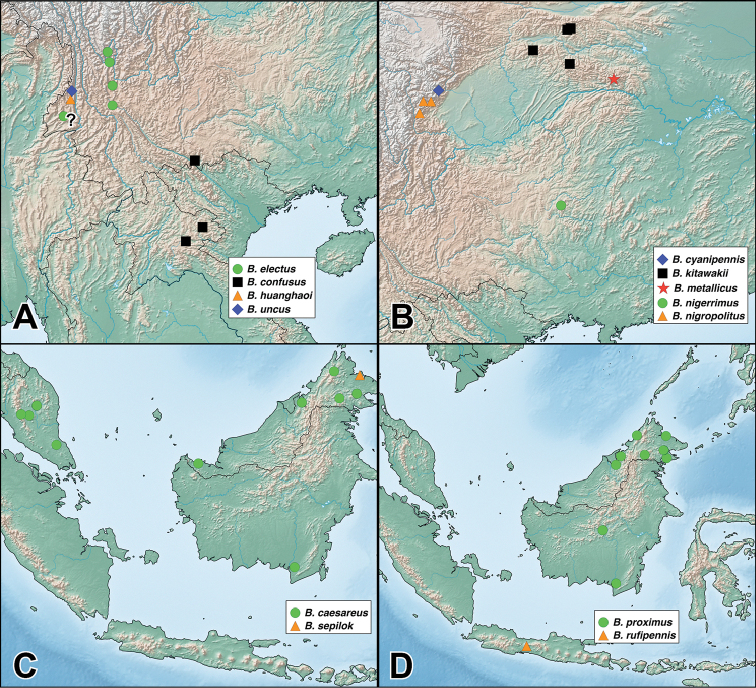
Distribution of: *Bolitogyrus electus* Smetana & Zheng, *B. confusus* Brunke, *B. huanghaoi* Hu et al., and *B. uncus* Cai et al. (**A**); *B. cyanipennis* (Zheng), *B. kitawakii* Smetana & Zheng, *B. metallicus* Cai et al., *B. nigerrimus* Yuan et al., and *B. nigropolitus* Smetana (**B**); *B. caesareus* (Bernhauer) and *B. sepilok* Brunke (**C**); *B. proximus* (Cameron) and *B. rufipennis* (Cameron) (**D**).

**Figure 20.**
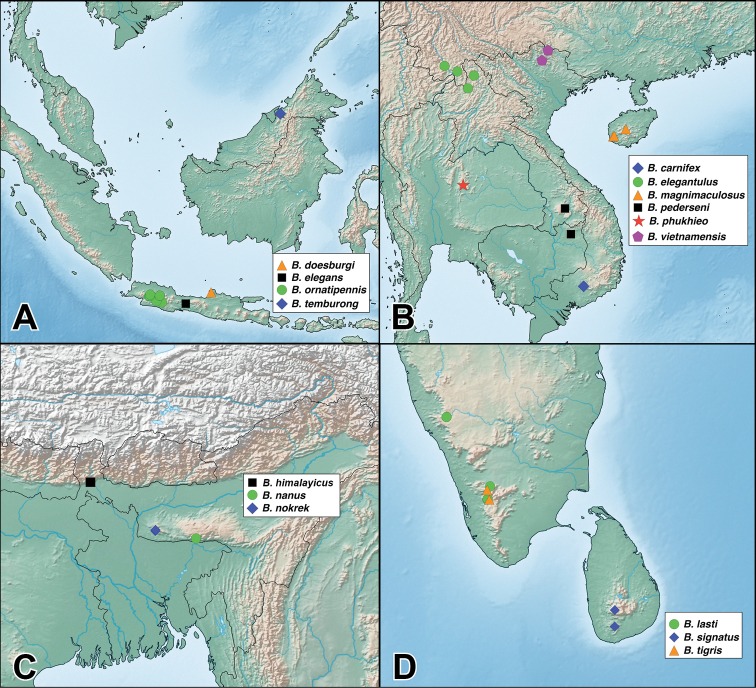
Distribution of: *Bolitogyrus doesburgi* (Scheerpeltz), *B. elegans* (Cameron), *B. ornatipennis* (Wendeler) and *B. temburong* Brunke (**A**); *B. carnifex* (Fauvel), *B. elegantulus* Yuan et al., *B. magnimaculosus* Cai et al., *B. pederseni* Brunke, *B. phukhieo* Brunke and *B. vietnamensis* (Scheerpeltz) (**B**); *B. himalayicus* Brunke, *B. nanus* Brunke and *B. nokrek* Brunke (**C**); *B. lasti* Rougemont, *B. signatus* (Cameron) and *B. tigris* Brunke (**D**).

**Figure 21.**
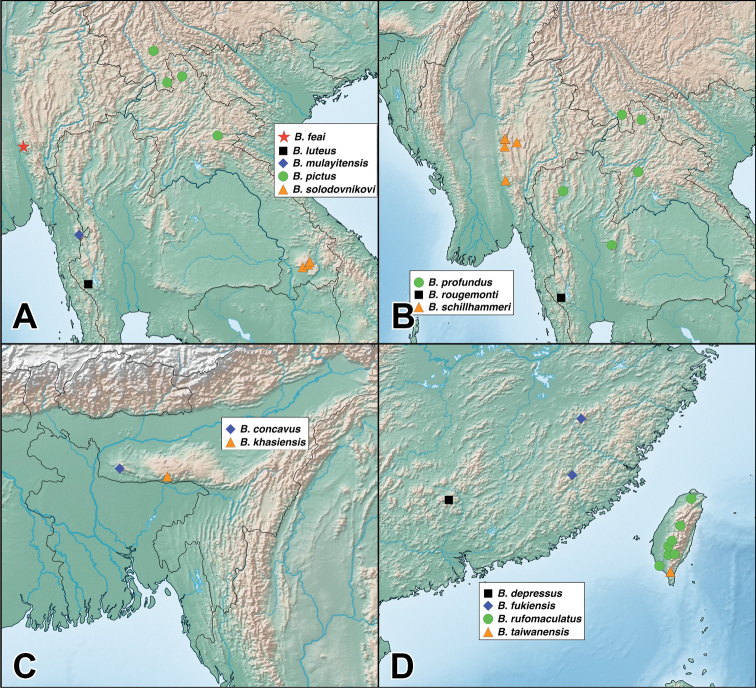
Distribution of: *Bolitogyrus feai* Brunke, *B. luteus* Brunke, *B. mulayitensis* Brunke, *B. pictus* Smetana & Zheng, and *B. solodovnikovi* Brunke (**A**); *B. profundus* Cai et al., *B. rougemonti* Brunke, and *B. schillhammeri* Brunke (**B**); *B. concavus* Brunke and *B. khasiensis* Brunke (**C**); *B. depressus* Cai et al., *B. fukiensis* (Scheerpeltz), *B. rufomaculatus* (Shibata) and *B. taiwanensis* (Hayashi) (**D**).

**Figure 22.**
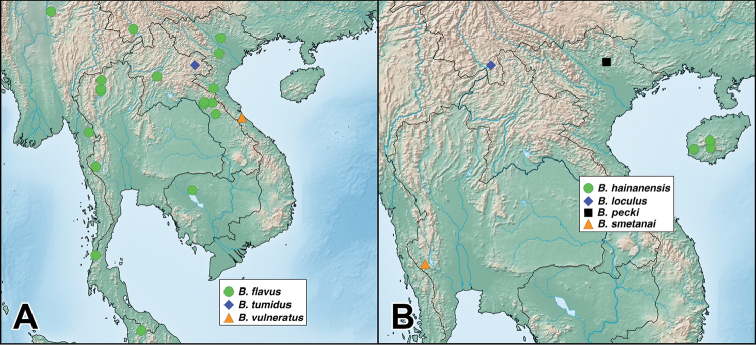
Distribution of: *Bolitogyrus flavus* Yuan et al., *B. tumidus* Brunke, and *B. vulneratus* (Fauvel) (**A**); *B. hainanensis* Cai et al., *B. loculus* Cai et al., *B. pecki* Brunke and *B. smetanai* Brunke (**B**).

## Supplementary Material

XML Treatment for Bolitogyrus electus

XML Treatment for Bolitogyrus uncus

XML Treatment for Bolitogyrus confusus

XML Treatment for Bolitogyrus huanghaoi

XML Treatment for Bolitogyrus nigropolitus

XML Treatment for Bolitogyrus metallicus

XML Treatment for Bolitogyrus nigerrimus

XML Treatment for Bolitogyrus cyanipennis

XML Treatment for Bolitogyrus kitawakii

XML Treatment for Bolitogyrus caesareus

XML Treatment for Bolitogyrus proximus

XML Treatment for Bolitogyrus temburong

XML Treatment for Bolitogyrus rufipennis

XML Treatment for Bolitogyrus carnifex

XML Treatment for Bolitogyrus pederseni

XML Treatment for Bolitogyrus vietnamensis

XML Treatment for Bolitogyrus elegantulus

XML Treatment for Bolitogyrus phukhieo

XML Treatment for Bolitogyrus magnimaculosus

XML Treatment for Bolitogyrus nokrek

XML Treatment for Bolitogyrus lasti

XML Treatment for Bolitogyrus tigris

XML Treatment for Bolitogyrus luteus

XML Treatment for Bolitogyrus sepilok

XML Treatment for Bolitogyrus pictus

XML Treatment for Bolitogyrus schillhammeri

XML Treatment for Bolitogyrus rougemonti

XML Treatment for Bolitogyrus profundus

XML Treatment for Bolitogyrus concavus

XML Treatment for Bolitogyrus vulneratus

XML Treatment for Bolitogyrus flavus

XML Treatment for Bolitogyrus rufomaculatus

XML Treatment for Bolitogyrus depressus

XML Treatment for Bolitogyrus tumidus

XML Treatment for Bolitogyrus taiwanensis

XML Treatment for Bolitogyrus fukiensis

XML Treatment for Bolitogyrus loculus

XML Treatment for Bolitogyrus hainanensis

XML Treatment for Bolitogyrus solodovnikovi

XML Treatment for Bolitogyrus feai

XML Treatment for Bolitogyrus mulayitensis

XML Treatment for Bolitogyrus smetanai

XML Treatment for Bolitogyrus khasiensis

XML Treatment for Bolitogyrus himalayicus

XML Treatment for Bolitogyrus nanus

XML Treatment for Bolitogyrus pecki

XML Treatment for Bolitogyrus elegans

XML Treatment for Bolitogyrus ornatipennis

XML Treatment for Bolitogyrus doesburgi

XML Treatment for Bolitogyrus signatus
